# Ca^2+^-saturated calmodulin binds tightly to the N-terminal domain of A-type fibroblast growth factor homologous factors

**DOI:** 10.1016/j.jbc.2021.100458

**Published:** 2021-02-24

**Authors:** Ryan Mahling, Cade R. Rahlf, Samuel C. Hansen, Matthew R. Hayden, Madeline A. Shea

**Affiliations:** Department of Biochemistry, Carver College of Medicine, University of Iowa, Iowa City, Iowa, USA

**Keywords:** molecular recognition, Ca^2+^-dependent interaction, voltage-gated sodium channel, protein–protein interaction, NMR, FRET, FHF, biosensor, thermodynamics, allostery, affinity, CaM, calmodulin, CaMBD, CaM-binding domain, CTD, C-terminal domain, FGF, fibroblast growth factor homologous factor, Nav, voltage-gated sodium channel, NTD, N-terminal domain

## Abstract

Voltage-gated sodium channels (Na_v_s) are tightly regulated by multiple conserved auxiliary proteins, including the four fibroblast growth factor homologous factors (FGFs), which bind the Na_v_ EF-hand like domain (EFL), and calmodulin (CaM), a multifunctional messenger protein that binds the Na_V_ IQ motif. The EFL domain and IQ motif are contiguous regions of Na_V_ cytosolic C-terminal domains (CTD), placing CaM and FGF in close proximity. However, whether the FGFs and CaM act independently, directly associate, or operate through allosteric interactions to regulate channel function is unknown. Titrations monitored by steady-state fluorescence spectroscopy, structural studies with solution NMR, and computational modeling demonstrated for the first time that both domains of (Ca^2+^)_4_-CaM (but not apo CaM) directly bind two sites in the N-terminal domain (NTD) of A-type FGF splice variants (FGF11A, FGF12A, FGF13A, and FGF14A) with high affinity. The weaker of the (Ca^2+^)_4_-CaM-binding sites was known *via* electrophysiology to have a role in long-term inactivation of the channel but not known to bind CaM. FGF12A binding to a complex of CaM associated with a fragment of the Na_V_1.2 CTD increased the Ca^2+^-binding affinity of both CaM domains, consistent with (Ca^2+^)_4_-CaM interacting preferentially with its higher-affinity site in the FGF12A NTD. Thus, A-type FGFs can compete with Na_V_ IQ motifs for (Ca^2+^)_4_-CaM. During spikes in the cytosolic Ca^2+^ concentration that accompany an action potential, CaM may translocate from the Na_V_ IQ motif to the FGF NTD, or the A-type FGF NTD may recruit a second molecule of CaM to the channel.

The human voltage-gated sodium channels (Na_V_) are a family of nine proteins (Na_V_1.1–Na_V_1.9) that are responsible for the generation and propagation of action potentials in excitable tissues throughout the human body. The functional core of each Na_V_ is a single transmembrane pore-forming α-subunit that interacts with one or more auxiliary β-subunits (Fig. 1, *A* and *B*) (1). The physiological function of Na_V_s requires the α-subunit to rapidly transition among closed, opened, and inactivated states. The importance of rapidly cycling among these functional states is highlighted by the identification of disease-causing mutations throughout the sequences of the Na_V_ isoforms that disrupt this process to cause debilitating conditions including epileptic disorders (2, 3, 4, 5, 6), cardiomyopathies (7, 8, 9, 10), and chronic pain (11, 12, 13).

**Figure 1:**
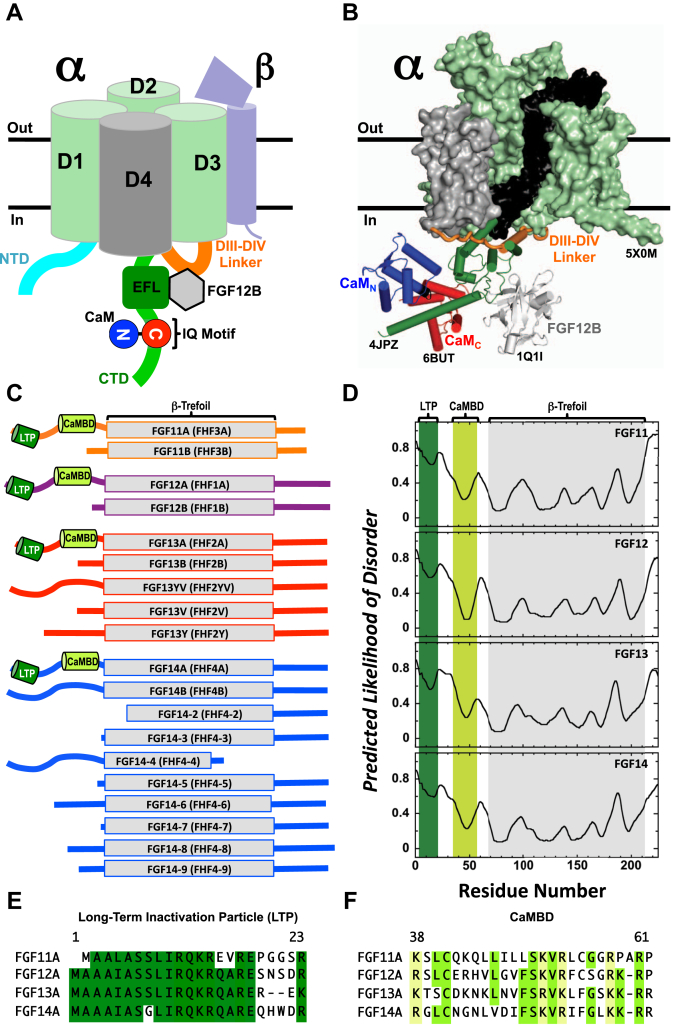
**Architecture of Na**_**V**_**, CaM, and FGFs.***A*, schematic of human Na_V_. The α-subunit N-terminal domain (NTD, *cyan*), transmembrane domains DI-DIII (*pale green*) and DIV (*gray*), the linker connecting DIII to DIV (*orange*), and C-terminal domain (CTD, *green*) with 4-helix bundle EFL (*dark green*) that binds FGFs (*light gray*), and IQ motif that binds CaM (CaM_N_/*blue*, CaM_C_/*red*) are shown. Auxiliary β-subunit that contains a transmembrane helix and extracellular domain is *lavender*. *B*, model of apo CaM and FGF12B bound to Na_V_. Model is comprised of Na_V_PAS (5X0M, DI-DIII/*pale green surface*, DIV voltage-sensing domain/*gray surface*, DIV pore domain/*black surface*, DIII-DIV linker/*orange cartoon*), the Na_V_1.5_CTD_ (4DCK, *forest green*), the apo CaM+Na_V_1.2_IQp_ ensemble (6BUT, CaM_N_/*blue*, CaM_C_/*red*), and FGF12B (1Q1U, *gray*). The Na_V_1.5_CTD_ was aligned to Na_V_PAS EFL (a.a. 1426–1521), the apo CaM+Na_V_1.2_IQp_ ensemble was aligned with 4DCK *via* CaM a.a. 101–112 and 117–128, and FGF12B was aligned with 4DCK using FGF13B a.a. 11–158. For simplicity Na_V_1.2_IQp_ in 6BUT and CaM FGF13B in 4DCK are not shown. *C*, schematics of FGF11 (*orange*), FGF12A (*purple*), FGF13A (*red*), and FGF14 (*blue*) splice variants. The β-trefoil core is shown as a *rectangle*, and the long-term inactivation particle (LTP, *green*) and putative CaM-binding domain (CaMBD, *limon*) are shown as cylinders. *D*, predicted likelihood of disorder of A-type FGF isoform sequences. The minima shaded *green* and *limon* correspond to the LTP and CaMBD sequences, respectively. The folded β-trefoil core is shaded *gray*. *E* and *F*, sequence alignments of the A-type FGF LTP (*E*) and CaMBD (*F*). Positions that are conserved in at least three of the four FGF isoforms are shaded (LTP/*gree*n, CaMBD/*limon*). Positions in the CaMBD (*F*) that contain a basic K or R in all four isoforms are shaded in *pale yellow*. Alignments were made with COBALT (108).

The transition of an Na_V_ α-subunit among its functional states is tightly regulated by a network of protein–protein interactions. These include intramolecular interactions among cytosolic regions of the α-subunit including the inactivation gate and intermolecular interactions between the cytosolic N-terminal (NTD) and C-terminal domains (CTD) of the channel and several auxiliary proteins (14, 15, 16). The Na_V_ CTD interacts with multiple auxiliary proteins including fibroblast growth factor homologous factors (FGFs or FHFs) that bind to an acidic EF-hand-like domain (EFL) (17, 18, 19, 20, 21) and the ubiquitously expressed and essential Ca^2+^ sensor calmodulin (CaM) that binds to a highly conserved basic IQ motif (IQxxx[R,K]Gxxx[R,K]) (Fig. 1*B*) (22, 23, 24, 25, 26, 27, 28).

CaM is composed of two four-helix bundle domains (CaM_N_ and CaM_C_). They are connected by a flexible linker that allows the domains to move independently in solution. Both CaM_N_ and CaM_C_ contain a pair of EF-hands that bind Ca^2+^ cooperatively. In free CaM, CaM_C_ has an affinity for Ca^2+^ that is approximately tenfold higher than that of CaM_N_ (29, 30, 31) resulting in sequential occupancy of the domains. In eukaryotes, CaM regulates many proteins in a Ca^2+^-dependent manner (32, 33, 34, 35, 36). Binding these targets selectively increases or decreases the Ca^2+^-binding affinity of one or both domains of CaM, making CaM an effective Ca^2+^ sensor over a wide range (10^3^) of Ca^2+^ concentrations (37).

Ca^2+^-depleted (apo) CaM and (Ca^2+^)_4_-CaM bind tightly to many IQ motifs. These basic amphipathic α-helix (BAA) CaM binding domains (CaMBDs) are found in all human Na_V_ isoforms, and CaM–Na_V_ interactions have been especially well studied in Na_V_1.2 and Na_V_1.5 (23, 24, 25, 26, 27). Despite both apo and (Ca^2+^)_4_-CaM having a high affinity for these IQ motifs, how CaM acts as a Ca^2+^ sensor to modulate Na_V_ function is poorly understood. Ca^2+^ binding to CaM_C_ induces a ∼180° rotation of CaM_C_ on the Na_V_1.2 IQ motif (25). This rotation may require transient release and reassociation of CaM_C_ with the IQ motif, which could also allow CaM to translocate to a different high-affinity CaMBD.

The four FGF isoforms (FGF11–FGF14) that bind the EFL domain of Na_V_s are a subgroup of the fibroblast growth factor superfamily (38). Crystallographic structures (39, 40) showed that they contain a well-folded β-trefoil core that is nearly identical to that of canonical fibroblast growth factors (41). However, unlike most members of the FGF family, these FGFs are not secreted (38, 42). Rather, they remain in the cytosol and have been implicated in trafficking and modulating Na_V_ channel properties including persistent current (17, 19, 21, 43, 44).

Multiple splice variants have been identified for each of the four FGF isoforms (Fig. 1*C*). These arise primarily from differential splicing of the first exon and result in sequences that vary in the length and composition of the NTD (42, 45). The effect of FGFs on Na_V_ function depends on the splice variant bound (21, 43, 46, 47). The B-type splice variants typically have a shorter NTD and are associated with changing current density. The A-type splice variants typically have a longer NTD and are specifically associated with an increased rate of inactivation and long-term inactivation of the Na_V_ α-subunit, which has been proposed to result from an interaction between a region in the NTD and the channel (43). The differences suggest important roles for the distinct NTD sequence of each FGF splice variant.

Colocalization experiments have found that CaM and the FGFs interact with multiple Na_V_ isoforms within cells (47, 48, 49, 50). Proteomics studies have shown that CaM and FGF12 interact with Na_V_1.2 in neurons (16). Crystallographic structures of a B-type FGF (FGF12B and FGF13U) and CaM bound to Na_V_ CTD fragments that contain both the EFL and IQ motif (26, 51) showed that CaM and FGFs are bound near each other. Recently, an indirect allosteric interaction has been proposed to occur between CaM and FGF12B on Na_V_1.4 (52). However, there has been no evidence supporting CaM directly binding any splice variant of an FGF isoform.

Here we report for the first time that CaM binds the NTD of each A-type FGF (FGF11A, FGF12A, FGF13A, and FGF14A) in a Ca^2+^-dependent manner. Using steady-state fluorescence spectroscopy, copurification, and solution NMR, we show that CaM binds two sequences in the NTD of each A-type FGF with high affinity and that both domains of (Ca^2+^)_4_-CaM mediate this interaction. Focusing on FGF12 because of the cellular and structural studies cited above, we demonstrate that binding of full-length FGF12A to a fragment of the Na_V_1.2 CTD (Na_V_1.2_CTD_, residues 1777–1937) containing the EFL and IQ motif increases the Ca^2+^ affinity of both CaM domains. Because the IQ motif is known to lower Ca^2+^ affinity of CaM_C_, this new finding is consistent with (Ca^2+^)_4_-CaM interacting favorably with the NTD of FGF12A in this ternary complex. These results support a model in which the A-type FGFs compete with Na_V_ IQ motifs for (Ca^2+^)_4_-CaM and suggest that, during spikes in the local cytosolic Ca^2+^ concentration, CaM may translocate from an Na_V_ IQ motif to an A-type FGF NTD or that the NTD may recruit an additional CaM molecule to the ternary complex.

## Results

### Potential CaM-binding sites in NTDs of A-type FGFs

CaM is known to bind tightly to sequences that are intrinsically disordered but that adopt helical geometry when bound by CaM. The NTD sequences of A-type splice variants of intracellular FGFs are thought to be disordered. However, an analysis of the FGF11A, FGF12A, FGF13A, and FGF14A sequences with the Protein Disorder Prediction System (53) showed two minima in the NTD of each FGF isoform (Fig. 1, *C* and *D*) suggesting that two segments are capable of adopting ordered secondary structure and might be CaM-binding sites.

One sequence is near the N terminus of the NTD (referred to as the long-term inactivation particle or LTP). It is highly conserved among the four human FGF isoforms (Fig. 1*E*) and across species (Fig. S1, *A–D*, Table S1–S4) and was shown to contribute functionally to long-term inactivation of Na_V_s mediated by A-type FGFs (43). The other sequence is C terminal to the LTP (referred to hereafter as the CaM-binding domain (CaMBD)). Although the CaMBD has a more variable sequence among the four human FGF isoforms (Fig. 1*F*), the CaMBD sequence of each isoform is highly conserved across species (Fig. S1, *E–H*, Table S5–S8). Currently it has no known function.

To explore whether the FGF LTP and CaMBD regions might function as CaM-binding sites, α-helical models were made with PyMOL and helical wheels were generated based on the sequence of the FGF12A LTP and CaMBD (Fig. S2, *A–F*). The sequences each contained an aliphatic patch bracketed by basic residues consistent with other BAA motifs that are known to bind tightly to CaM.

To understand whether these putative sites in the FGF NTD would be accessible to CaM, it would be helpful to have an experimentally determined structure; however, none are available for any of the full-length A-type FGFs. Therefore, structural models of full-length FGF12A were generated with Robetta (54). Each model in the ensemble had a well-folded β-trefoil core that was nearly identical to that of a crystallographically determined structure of FGF12B (Fig. S3, *A* and *B*) (39). New insights came from modeling of the FGF12A NTD that included the LTP and CaMBD. Both potential CaM-binding sites were predicted to adopt α-helical secondary structure (Fig. S3*B*). They were connected by a disordered linker (aa 18–39), and the CaMBD region was connected to the β-trefoil core by another disordered linker (aa 52–69). These linkers would allow the LTP and CaMBD to sample many orientations relative to each other and relative to the β-trefoil core as shown in the set of five lowest-energy (most favorable) conformations (Fig. S3, *C–H*). The predicted secondary structure and BAA motif sequences of the FGF12A LTP and CaMBD suggested that both were strong candidates for CaM binding.

### (Ca^2+^)_4_-saturated CaM tightly binds two sites in the A-type FGFs

To determine whether CaM binds the FGF LTP or CaMBD, apo or (Ca^2+^)_4_-CaM was added to biosensors in which the sequence of the LTP of FGF12A (FGF12A_LTP_, residues 1–23) or CaMBD of FGF11A (FGF11A_CaMBD_, residues 36–62), FGF12A (FGF12A_CaMBD_, residues 38–64), FGF13A (FGF13A_CaMBD_, residues 35–60), and FGF14A (FGF14A_CaMBD_, residues 37–63) was inserted between YFP and CFP (see Experimental procedures).

**Figure 2:**
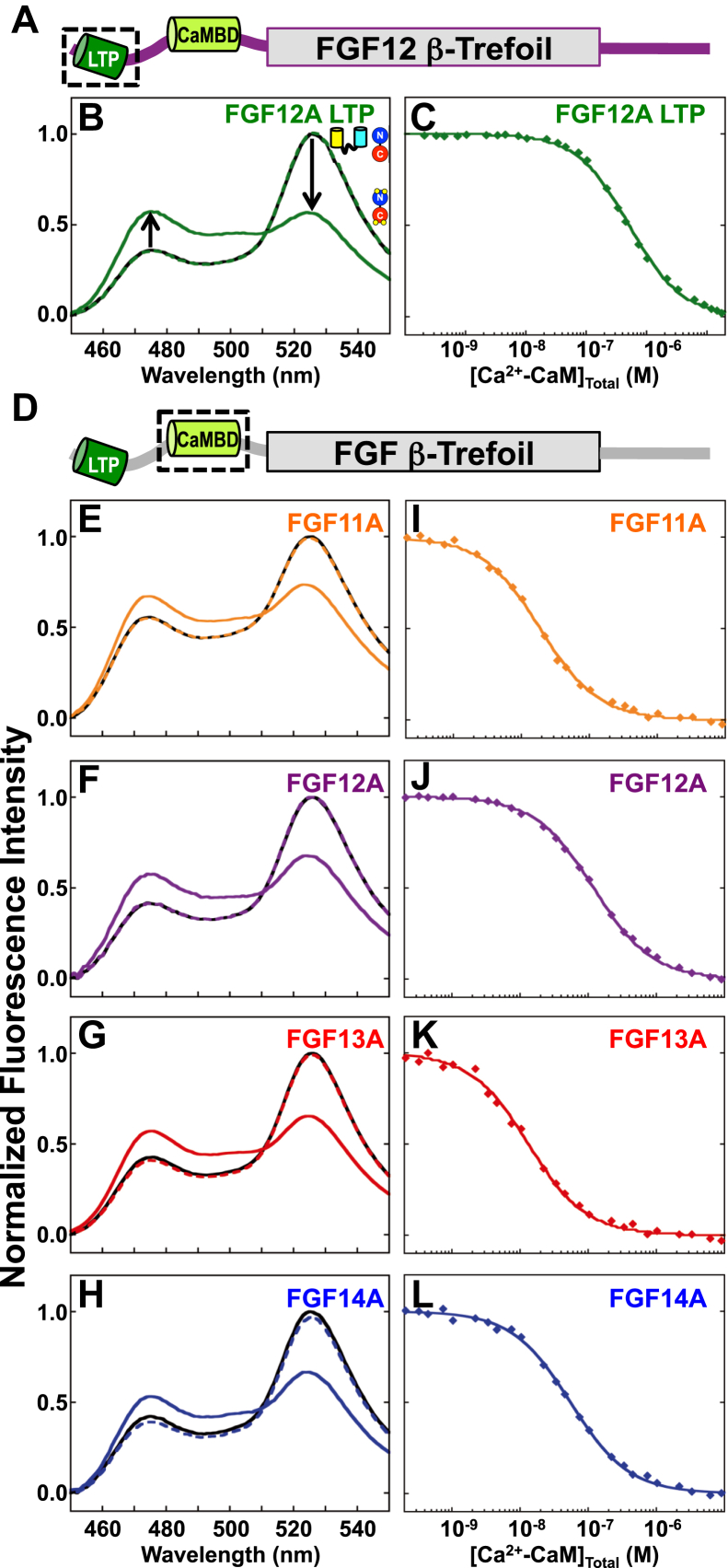
**(Ca**^**2+**^**)**_**4**_**-CaM binds the FGF12A**_**LTP**_**and FGF CaMBD biosensors.***A*, schematic of FGF12A. Folded β-trefoil core is shown as a *rectangle*, and LTP (*green*) and CaMBD (*limon*) are shown as *cylinders*. The *black box* indicates the position of the FGF12A LTP. *B*, steady-state emission spectra of the FGF12A_LTP_ biosensor alone (*solid black*), in the presence of apo (*dashed green*) and (Ca^2+^)_4_-CaM (*solid green*). *C*, equilibrium titration of FGF12A_LTP_ biosensor with (Ca^2+^)_4_-CaM (*green*). *D*, schematic of an A-type FGF. Folded β-trefoil core is shown as a *rectangle*, and LTP (*green*) and CaMBD (*limon green*) are shown as *cylinders*. The *black box* indicates the position of the A-type FGF CaMBD. *E–H*, steady-state fluorescence spectra of FGF11A_CaMBD_ (*E*, *orange*), FGF12A_CaMBD_ (*F*, *purple*), FGF13A_CaMBD_ (*G*, *red*), FGF14A_CaMBD_ (*H*, *blue*) biosensors alone (all shown in *solid black*), and in the presence of apo (*dashed/colored*) and (Ca^2+^)_4_-CaM (*solid/colored*). *I–L*, equilibrium titrations of FGF11A_CaMBD_ (*I*), FGF12A_CaMBD_ (*J*), FGF13A_CaMBD_ (*K*), and FGF14A_CaMBD_ (*L*) biosensors with (Ca^2+^)_4_-CaM. FGF isoforms are colored as in panels *E–H*.

Addition of excess apo CaM to the FGF12A_LTP_ biosensor (200:1 [CaM]:[Biosensor]) caused negligible (∼0%) changes in the emission spectrum (Fig. 2, *A* and *B*). However, after addition of saturating Ca^2+^, reciprocal changes were observed in the intensities of YFP (reduced by ∼50%) and CFP (increased by ∼52%) (Fig. 2*B*). This indicated that (Ca^2+^)_4_-CaM, but not apo CaM, bound to FGF12A_LTP_. Equilibrium titrations of the FGF12A_LTP_ biosensor with (Ca^2+^)_4_-CaM showed that the K_d_ was 576 nM (Fig. 2*C*, Table 1).

**Table 1:** FGF biosensor affinities for WT CaM

FGF_LTP_	ΔG^a^	K_d_^b^	N
FGF12A_LTP_	−8.42 ± 0.24	576 nM	11


FGF_CaMBD_	ΔG^a^	K_d_^b^	
FGF11A_CaMBD_	−10.43 ± 0.18	19 nM	7
FGF12A_CaMBD_	−9.40 ± 0.21	107 nM	9
FGF13A_CaMBD_	−10.63 ± 0.02	13 nM	6
FGF14A_CaMBD_	−9.87 ± 0.18	51 nM	7

a ΔG in kcal/mol. Average and standard deviation based on N determinations of biosensors prepared from at least two independent cultures.

b K_d_, equilibrium dissociation constant, calculated from average value of ΔG reported in this table. Solution Conditions: 50 mM HEPES, 100 mM KCl, 50 μM EGTA, 5 mM NTA, 1 mM MgCl_2,_ 1.5 μM BSA, 500 μM DTT, 1 mM CaCl_2_; pH 7.4, 22 °C.

As observed for FGF12A_LTP_, addition of excess apo CaM to the FGF11A_CaMBD_, FGF12A_CaMBD_, FGF13A_CaMBD_, or FGF14A_CaMBD_ biosensor (200:1 [CaM]:[Biosensor]) resulted in negligible spectral changes, while (Ca^2+^)_4_-CaM induced robust reciprocal changes in the intensities of YFP (-21%–33%) and CFP (18%–32%) (Fig. 2, *D*–*H*). Equilibrium titrations with (Ca^2+^)_4_-CaM showed that CaM bound the CaMBD of each FGF isoform with a different affinity (Fig. 2, *I*–*L*, Table 1), with FGF13A being most favorable (K_d_ = 13 nM) and FGF12A least favorable (K_d_ = 107 nM). The small difference in the affinity of (Ca^2+^)_4_-CaM for the CaMBD of FGF11A and FGF13A (0.18 kcal/mol) was statistically significant (*p* value 0.02358) but would have a very small effect on saturation.

These affinities were 5–44-fold more favorable than the affinity of (Ca^2+^)_4_-CaM for the FGF12A LTP (Fig. 2, *C* and *I–L*, Table 1). Based on the high degree of conservation in the LTP sequence of all four FGFs, these findings suggest that when a single (Ca^2+^)_4_-CaM is bound to a full-length A-type FGF, it would occupy the putative CaMBD rather than the LTP.

### Both CaM_N_ and CaM_C_ are required for tight binding to FGF CaMBDs

**Figure 3:**
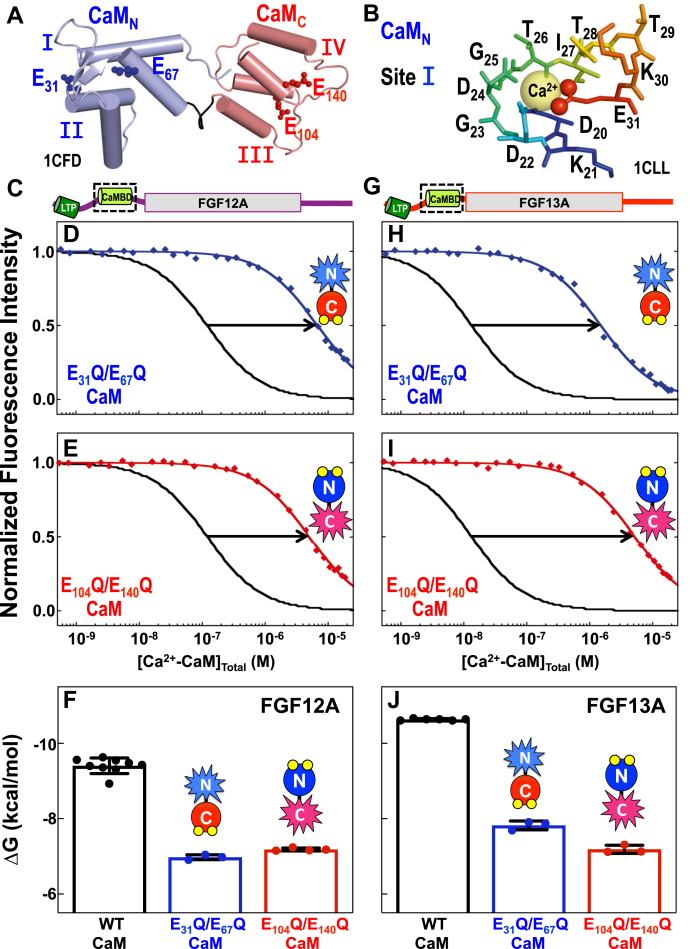
**Energetic contribution of CaM**_**N**_**and CaM**_**C**_**to FGF12A and FGF13A CaMBD binding by CaM.***A*, position of E31 (*blu*e), E67 (*blue*), E104 (*red*), and E140 (*red*), shown as ball-and-stick, in apo CaM (1CFD, CaM_N_/*light blue*, CaM_C_/*salmon*). *B*, (Ca^2+^)_4_-CaM site I (1CLL): Ca^2+^ (*yellow sphere*) is surrounded by residues D20 (*blue*) to E31 (*red*). The coordinating O atoms of E31 are shown as *red spheres*. *C*, schematic of FGF12A. Folded β-trefoil core is shown as a *rectangle*, and LTP (*green*) and CaMBD (*limon*) are shown as *cylinders*. The *black box* indicates the position of the FGF12A CaMBD. *D* and *E*, equilibrium titrations of the FGF12A_CaMBD_ biosensor with E31Q/E67Q (*D*, *blue*) or E104A/E140Q CaM (*E*, *red*). A reference titration with WT CaM is shown in *black*. *F*, ΔG of WT (*black*), E31Q/E67Q (*blue*), and E104Q/E140Q (*red*) CaM binding the FGF12A_CaMBD_ biosensor. *G*, schematic of FGF13A. Folded β-trefoil core is shown as a *rectangle*, and LTP (*green*) and CaMBD (*limon*) are shown as *cylinders*. The *black box* indicates the position of the FGF13A CaMBD. *H* and *I*, equilibrium titrations of the FGF13A_CaMBD_ biosensor with E31Q/E67Q (*H*, *blue*) or E104A/E140Q CaM (*I*, *red*). A reference titration with WT CaM is shown in *black*. *J*, ΔG of WT (*black*), E31Q/E67Q (*blue*), and E104Q/E140Q (*red*) CaM binding the FGF13A_CaMBD_ biosensor.

We assessed the energetic contributions of CaM_N_ and CaM_C_ to the binding of FGF12A_CaMBD_ and FGF13A_CaMBD_. Initial titrations with isolated CaM_N_ (aa 1–80) or CaM_C_ (aa 76–148) showed no evidence of binding at a concentration of 1 μM (data not shown). Because the interaction of CaM with FGF12A_CaMBD_ and FGF13A_CaMBD_ is Ca^2+^-dependent, the energetic contributions of CaM_N_ and CaM_C_ within full-length CaM were explored by determining their affinity for "knockout mutants" of CaM engineered to significantly reduce Ca^2+^-binding to one domain. In these mutants the bidentate Glu (position 12) in sites I and II (E31Q/E67Q) or sites III and IV (E104Q/E140Q) was replaced with Gln (Fig. 3, *A* and *B*) (31). In these mutants with one domain mutated, the other domain retains a high Ca^2+^-binding affinity (31).

Equilibrium titrations of FGF12A_CaMBD_ with these mutants showed that E31Q/E67Q CaM bound to the CaMBD with a K_d_ of 6.74 μM, a 63-fold lower affinity than WT CaM, while E104Q/E140Q CaM bound with a K_d_ of 4.73 μM, a 44-fold lower affinity (Fig. 3, *C*–*E*, Table 2). The affinities of the knockout mutants were very close (Fig. 3*F*), suggesting that CaM_N_ and CaM_C_ contribute similarly to binding FGF12A_CaMBD_. Compared with the pattern observed for FGF12A_CaMBD_, the threefold difference in the affinity of E31Q/E67Q CaM (K_d_ = 1.59 μM, 122-fold weaker than WT) and E104Q/E140Q CaM (K_d_ = 4.73 μM, 363-fold weaker than WT) (Fig. 3, *G*–*I*, Table 2) for FGF13A_CaMBD_ indicated that CaM_C_ makes an energetic contribution to binding FGF13A_CaMBD_ that is larger than that made by CaM_N_.

**Table 2:** FGF biosensor affinities for mutant CaM

CaM_1-148_	FGF12A_CaMBD_			FGF13A_CaMBD_		
	ΔG^a^	K_d_^b^	N	ΔG^a^	K_d_^b^	N
E31Q/E67Q	−6.98 ± 0.07	6.74 μM	3	−7.82 ± 0.12	1.59 μM	3
E104Q/E140Q	−7.18 ± 0.04	4.73 μM	4	−7.18 ± 0.11	4.72 μM	3

a ΔG in kcal/mol. Average and standard deviation based on N titrations of biosensors prepared from at least two independent cultures.

b K_d_, equilibrium dissociation constant, calculated from average value of ΔG reported in this table. Solution Conditions: 50 mM HEPES, 100 mM KCl, 50 μM EGTA, 5 mM NTA, 1 mM MgCl_2,_ 1.5 μM BSA, 500 μM DTT, 1 mM CaCl_2_; pH 7.4, 22 °C.

The mutant E104Q/E140Q CaM contains only a functional CaM_N_. Given that its affinity for FGF13A_CaMBD_ and FGF12A_CaMBD_ was identical (Fig. 3, *F* and *J*, Table 2), this suggests that the separation in the affinity of WT CaM for the CaMBD of FGF12A and FGF13A may result from differences in the interface between CaM_C_ and the CaMBD.

### Stoichiometry of copurified (Ca^2+^)_4_-CaM+FGF NTD complexes

The finding that (Ca^2+^)_4_-CaM can bind to the isolated FGF LTP (Fig. 2, *A*–*C*) and CaMBD (Fig. 2, *D*–*L*) suggests that the NTD of an A-type FGF may bind two molecules of CaM simultaneously. To test this hypothesis, we utilized revered-phase high-performance liquid chromatography (rpHPLC) to determine the molar ratio of (Ca^2+^)_4_-CaM to FGF NTD in copurified complexes of (Ca^2+^)_4_-CaM bound to NTD fragments (residues ∼1–70, containing both the LTP and CaMBD), of FGF11A (FGF11A_NTD_), FGF12A (FGF12A_NTD_), FGF13A (FGF13A_NTD_), and FGF14A (FGF14A_NTD_) (Fig. S4, *A* and *B*).

In rpHPLC chromatograms (Fig. S4*B*) of the copurified complexes, the ratio of the integrated area under the absorbance peaks corresponding to the FGF NTD and (Ca^2+^)_4_-CaM showed that each sample contained a 1:1 M ratio of (Ca^2+^)_4_-CaM to FGF NTD (Fig. S4*C*). This is consistent with the isolated NTD fragment of each A-type FGF binding a single molecule of (Ca^2+^)_4_-CaM following copurification. However, these results do not indicate the location(s) of CaM and do not discriminate among the possibilities of having a single (Ca^2+^)_4_-CaM bound to an LTP or CaMBD site alone, or possibly bridging these two sites with one CaM domain bound to each. Furthermore, these results do not preclude the possibility that a second molecule of (Ca^2+^)_4_-CaM may bind the NTD if the local CaM concentration was sufficiently high.

### CaM_N_ and CaM_C_ bind identically to FGF12A_CaMBDp_ and FGF12A_NTD_

Solution NMR is uniquely capable of monitoring changes in the local environment of individual residues within a protein. To determine how the two domains of (Ca^2+^)_4_-CaM rearrange and interact with an FGF NTD at a one-to-one molar ratio, solution NMR was used to monitor FGF12A CaMBD and FGF12A_NTD_-induced changes in the local environment of residues in labeled (Ca^2+^)_4_-CaM.

**Figure 4:**
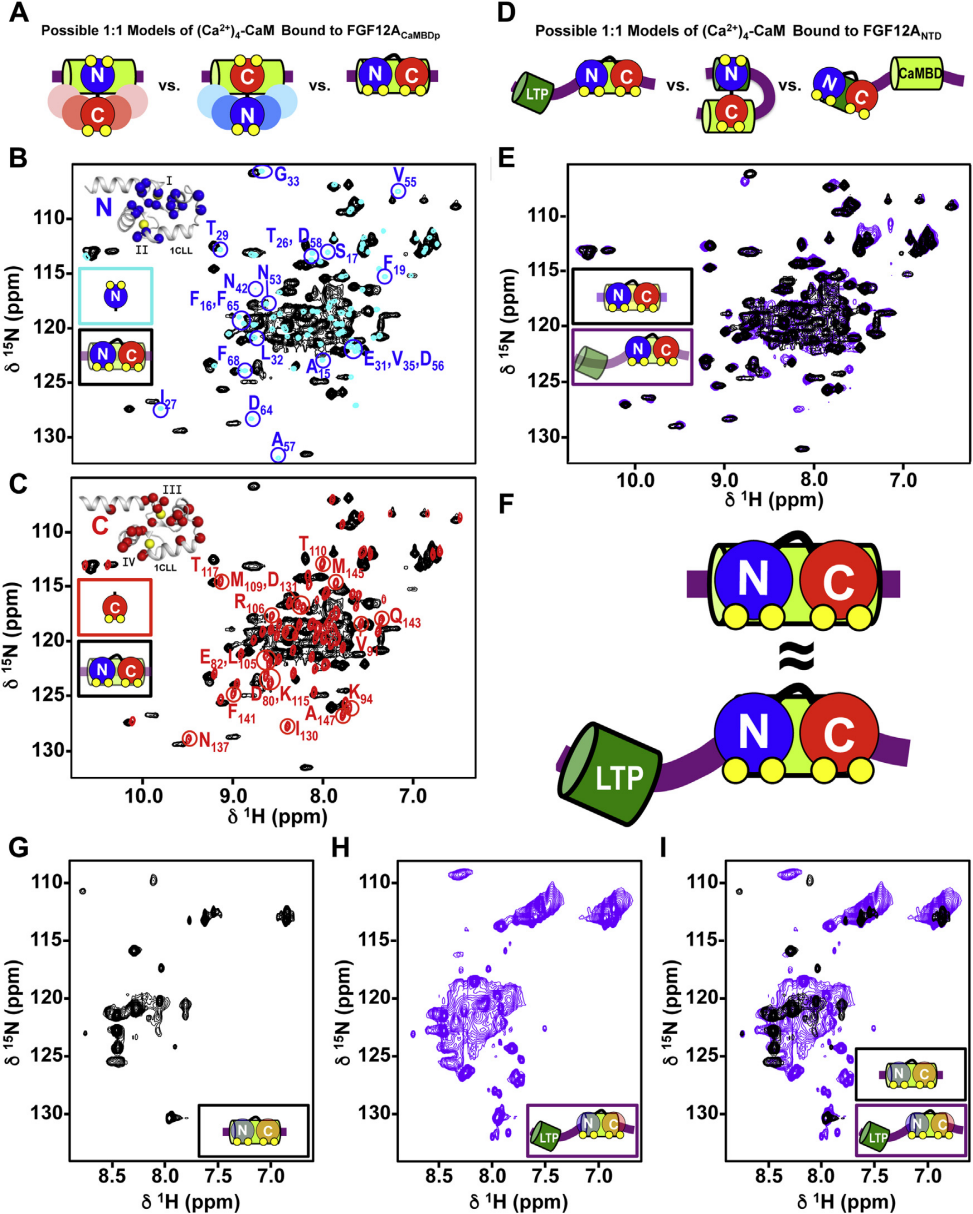
**Interaction of (Ca**^**2+**^**)**_**4**_**-CaM with the FGF12A**_**CaMBDp**_**and FGF12A**_**NTD**._*A*, schematics depicting models of (Ca^2+^)_4_-CaM (CaM_N_/*blue*, CaM_C_/*red*) binding to the FGF12A_CaMBDp_ (*limon cylinder*) in a 1: 1 M ratio. *B* and *C*, overlay of the ^15^N-HSQC spectra of ^15^N-(Ca^2+^)_2_-CaM_N_ (*blue*, *B*) or ^15^N-(Ca^2+^)_2_-CaM_C_ (*red*, *C*) and ^15^N-(Ca^2+^)_4_-CaM+^14^N-FGF12A_CaMBDp_ (*black*). Peaks labeled in the spectrum of ^15^N-(Ca^2+^)_2_-CaM_N_ (*B*) or ^15^N-(Ca^2+^)_2_-CaM_C_ (*C*) are shifted in the ^15^N-(Ca^2+^)_4_-CaM+^14^N-FGF12A_CaMBD_ spectrum. *Insets* show the Cα of labeled CaM_N_ (*blue spheres*, *B*) or CaM_C_ (*red spheres*, *C*) residues on a structure of (Ca^2+^)_4_-CaM (1CLL, *gray helices*). *D*, schematics depicting models of (Ca^2+^)_4_-CaM (CaM_N_/*blue*, CaM_C_/*red*) binding to the FGF12A_NTD_ in a 1:1 M ratio. FGF12A LTP (*green*) and CaMBD (*limon*) are shown as cylinders. *E*, overlay of the ^15^N-HSQC spectra of ^15^N-(Ca^2+^)_4_-CaM bound to the ^14^N-FGF12A_CaMBDp_ (*black*) or ^14^N-FGF12A_NTD_ (*purple*). *F*, schematic showing that the interface is essentially identical between (Ca^2+^)_4_-CaM (CaM_N_/*blue*, CaM_C_/*red*) and the FGF12A_CaMBDp_ (*limon cylinder*) or FGF12A_NTD_ (LTP/*green cylinder*, CaMBD/*limon cylinder*). *G* and *H*, ^15^N-HSQC spectrum of ^14^N-(Ca^2+^)_4_-CaM bound to ^15^N-FGF12A_CaMBDp_ (G, *black*) or ^15^N-FGF12A_NTD_ (*H*, *purple*). *I*, overlay of the ^15^N-HSQC spectra of ^14^N-(Ca^2+^)_4_-CaM bound to ^15^N-FGF12A_CaMBDp_ (*black*) or ^15^N-FGF12A_NTD_ (*purple*).

We first sought to determine how binding of the isolated CaMBD of FGF12A (Fig. 4*A*) changed CaM_N_ and CaM_C_ within (Ca^2+^)_4_-CaM. To do this we compared the ^15^N-HSQC spectrum of ^15^N-(Ca^2+^)_4_-CaM bound to an unlabeled C-terminal fragment of the FGF12A_NTD_ (^14^N-FGF12A_CaMBDp_, residues 41–70), corresponding to roughly half of the FGF12A_NTD_, to spectra of isolated (Ca^2+^)_2_-CaM_N_ (Fig. 4*B*) and (Ca^2+^)_2_-CaM_C_ (Fig. 4*C*). These showed that FGF12A_CaMBDp_ binding induced changes in the chemical shifts of residues throughout CaM_N_ and CaM_C_, which is consistent with both domains of (Ca^2+^)_4_-CaM interacting directly with FGF12A_CaMBDp_.

To determine how (Ca^2+^)_4_-CaM interacts with the full NTD that contains both the LTP and CaMBD sequences (Fig. 4*D*), we compared the ^15^N-HSQC spectrum of ^15^N-(Ca^2+^)_4_-CaM bound to the complete unlabeled NTD of FGF12A (^14^N-FGF12A_NTD_) to that of ^15^N-(Ca^2+^)_4_-CaM bound to the ^14^N-FGF12A_CaMBDp_. As observed in the 1:1 complex of CaM bound to the FGF12A_CaMBDp_, the binding of FGF12A_NTD_ changed the local environment of residues throughout CaM_N_ (Fig. S5, *A* and *B*) and CaM_C_ (Fig. S5, *C* and *D*). This conclusion alone would be consistent with either CaM bridging the LTP and CaMBD or CaM binding either of these sites exclusively.

Comparison of the ^15^N-HSQC spectrum of ^15^N-(Ca^2+^)_4_-CaM+^14^N-FGF12A_CaMBDp_ to that of ^15^N-(Ca^2+^)_4_-CaM+^14^N-FGF12A_NTD_ revealed that peaks corresponding to (Ca^2+^)_4_-CaM residues were in nearly identical positions in both spectra (Fig. 4*E*). This indicated that residues in (Ca^2+^)_4_-CaM have equivalent local environments when bound to the FGF12A_CaMBDp_ or FGF12A_NTD_, suggesting that the interface between (Ca^2+^)_4_-CaM and the FGF12A_NTD_ is identical to that of (Ca^2+^)_4_-CaM+FGF12A_CaMBDp_ (Fig. 4*F*). The simplest explanation is that (Ca^2+^)_4_-CaM binds the FGF12A_NTD_ exclusively through the CaMBD, with neither domain of CaM making persistent contacts with the LTP. There was no evidence for more than one conformation though we cannot exclude the possibility that some additional conformations were populated in low abundance.

### FGF12A_CaMBDp_ and FGF12A_NTD_ respond similarly to (Ca^2+^)_4_-CaM binding

To probe the interface between (Ca^2+^)_4_-CaM and the FGF12A_NTD_ from the FGF side, we used solution NMR to examine the effect of unlabeled (Ca^2+^)_4_-CaM (^14^N-(Ca^2+^)_4_-CaM) binding on the local chemical environment of residues in the labeled FGF12A_CaMBDp_ (^15^N-FGF12A_CaMBDp_) and FGF12A_NTD_ (^15^N-FGF12A_NTD_). The isolated FGF12A_CaMBDp_ and FGF12A_NTD_ were not soluble at the concentrations needed for NMR studies. Thus, we made a pairwise comparison of the local chemical environment of FGF residues in ^15^N-FGF12A_CaMBDp_ (Fig. 4*G*) to those in ^15^N-FGF12A_NTD_ (Fig. 4*H*) when each was bound to ^14^N-(Ca^2+^)_4_-CaM. This was illuminating regarding the preference of CaM for the CaMBD sequence relative to the LTP sequence.

The majority of the peaks in the ^15^N-HSQC spectrum of ^15^N-FGF12A_CaMBDp_ or ^15^N-FGF12A_NTD_ bound to ^14^N-(Ca^2+^)_4_-CaM had a ^1^H chemical shift between 8.0 and 8.5 ppm (Fig. 4, *G* and *H*). Peaks in the^15^N-HSQC spectrum of ^14^N-(Ca^2+^)_4_-CaM+^15^N-FGF12A_CaMBDp_ were relatively well dispersed (Fig. 4*G*), which was likely due to the small size of this peptide (30 FGF12A residues with a four-residue tag). In contrast, the spectrum of ^14^N-(Ca^2+^)_4_-CaM+^15^N-FGF12A_NTD_ was more crowded, as expected for a larger fragment (70 FGF12A residues with a four-residue tag) (Fig. 4*H*). The higher degree of overlap in that spectrum likely reflects the presence of a disordered linker between the LTP and CaMBD, as predicted in the Robetta models of full-length FGF12A (Fig. S3, *B–H*).

To assess whether the pattern of peaks in the ^15^N-HSQC spectra of ^14^N-(Ca^2+^)_4_-CaM+^15^N-FGF12A_CaMBDp_ and ^14^N-(Ca^2+^)_4_-CaM+^15^N-FGF12A_NTD_ were consistent with the predicted α-helical secondary structure of the FGF12A LTP and CaMBD, the observed peak positions were compared with those predicted with SPARTA+ (55) for residues 41–70 (FGF12A_CaMBDp_) and 1–70 (FGF12A_NTD_) (Fig. S6, *A–D*) from the model of full-length FGF12A. Peak positions in the ^15^N-HSQC spectra of ^14^N-(Ca^2+^)_4_-CaM+^15^N-FGF12A_CaMBDp_ (Fig. S6*C*) or +^15^N-FGF12A_NTD_ (Fig. S6*D*) agreed well with those predicted from the fragments of the FGF12A model. We inferred that both the FGF12A LTP and CaMBD adopted an α-helical structure in the complexes of (Ca^2+^)_4_-CaM bound to the FGF12A_CaMBDp_ or FGF12A_NTD_.

Comparison of the ^15^N^-^HSQC spectrum of ^14^N-(Ca^2+^)_4_-CaM+^15^N-FGF12A_CaMBDp_ (Fig. 4*G*) to that of ^14^N-(Ca^2+^)_4_-CaM+^15^N-FGF12A_NTD_ (Fig. 4*H*) showed that a subset of peaks in the ^14^N-(Ca^2+^)_4_-CaM+^15^N-FGF12A_NTD_ spectrum were located at positions essentially equivalent to those of peaks in the ^14^N-(Ca^2+^)_4_-CaM+^15^N-FGF12A_CaMBDp_ spectrum (Fig. 4*I*). This suggests that these peaks correspond to the same residues in the FGF12A_CaMBDp_ and FGF12A_NTD_ and that they have an essentially identical local chemical environment when bound by (Ca^2+^)_4_-CaM. That supports a model where (Ca^2+^)_4_-CaM is anchored to the FGF12A_NTD_ *via* the CaMBD sequence when in a one-to-one complex as shown schematically in Figure 4*F*.

### FGF12A_CaMBDp_ and FGF12A_NTD_ increase the Ca^2+^ affinity of CaM_N_ and CaM_C_

To quantitatively assess the allosteric effect of FGF12A_NTD_ and FGF12A_CaMBDp_ on the Ca^2+^-binding affinity of CaM_N_ and CaM_C_, we conducted equilibrium Ca^2+^ titrations of CaM in the presence of the FGF12A_NTD_ or FGF12A_CaMBDp_ by monitoring changes in the steady-state fluorescence intensity of endogenous Phe and Tyr residues to detect Ca^2+^ binding by CaM_N_ and CaM_C_, respectively (31).

**Figure 5:**
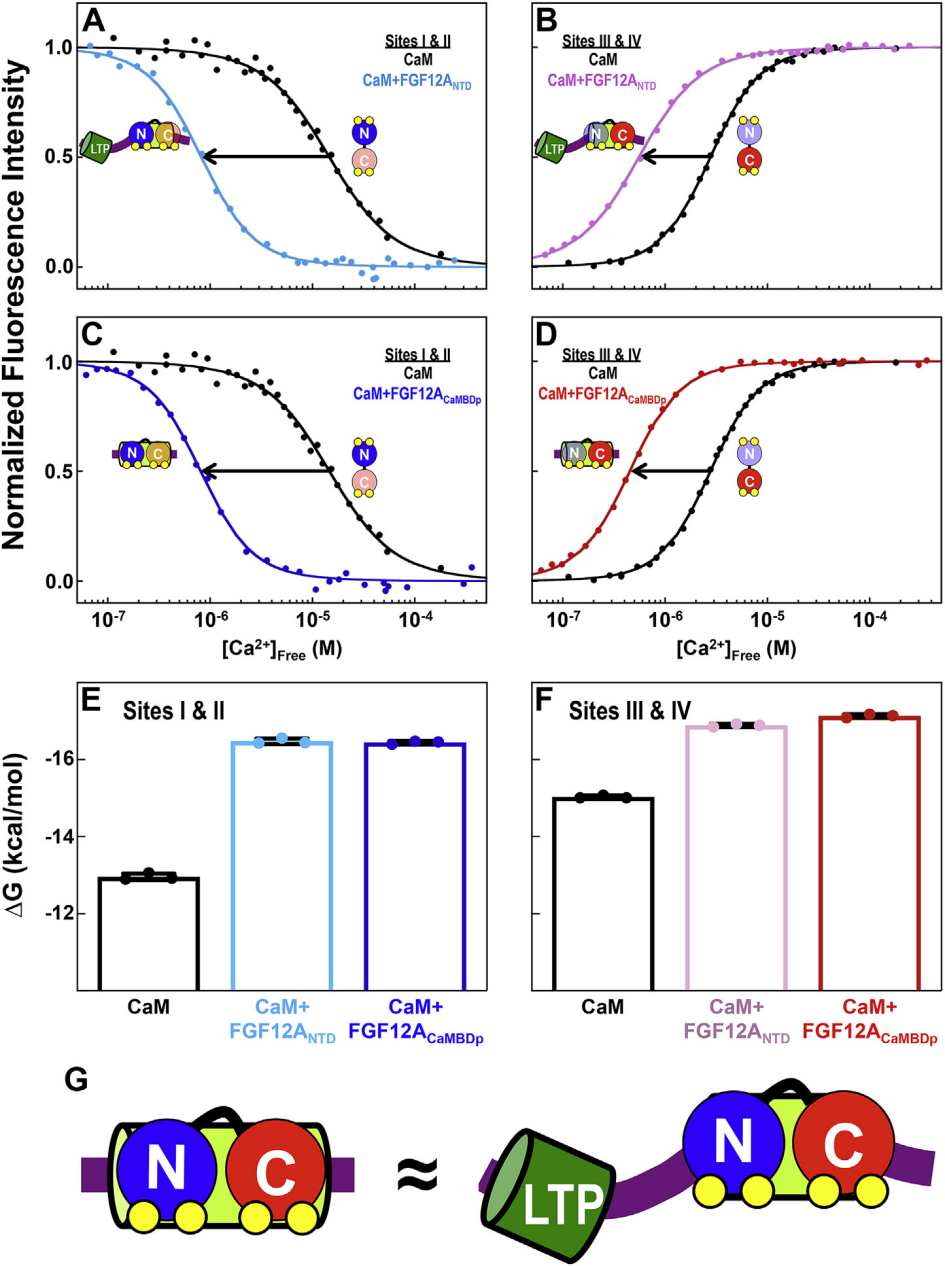
**Equilibrium Ca**^**2+**^**titrations of CaM+FGF12A**_**CaMBDp**_**and CaM+FGF12A**_**NTD**_. *A*, equilibrium Ca^2+^ titrations of sites I and II of CaM alone (*black*) and in the presence of the FGF12A_NTD_ (*teal*). *B*, equilibrium Ca^2+^ titrations of sites III and IV of CaM alone (*black*) and in the presence of the FGF12A_NTD_ (*pink*). *C*, equilibrium Ca^2+^ titrations of sites I and II of CaM alone (*black*) and in the presence of the FGF12A_CaMBDp_ (*blue*). *D*, equilibrium Ca^2+^ titrations of sites III and IV of CaM alone (*black*) and in the presence of the FGF12A_CaMBDp_ (*red*). *E* and *F*, ΔG of Ca^2+^ binding to sites I and II (*E*) in CaM alone (*black*), +FGF12A_NTD_ (*teal*) or +FGF12A_CaMBDp_ (*blue*), and sites III and IV (*F*) in CaM alone (*black*), +FGF12A_NTD_ (*pink*) or +FGF12A_CaMBDp_ (*red*). *G*, schematic depicting that the interface is essentially identical between (Ca^2+^)_4_-CaM (CaM_N_/*blue*, CaM_C_/*red*) and the FGF12A_CaMBDp_ (*lime green cylinder*) or FGF12A_NTD_ (LTP/*green cylinder*, CaMBD/*limon cylinder*).

Equilibrium Ca^2+^ titrations of CaM bound to FGF12A_NTD_ showed it increased the Ca^2+^-binding affinity of sites I and II in CaM_N_ (K_d-app_ = 0.81 μM) by ∼20-fold (Fig. 5*A*, Table 3) and sites III and IV in CaM_C_ (K_d-app_ = 0.45 μM) by approximately fivefold (Fig. 5*B*, Table 3) relative to free CaM. In a similar pattern, FGF12A_CaMBDp_ increased the affinity of sites I and II (K_d-app_ = 0.79 μM) by ∼20-fold relative to CaM alone (Fig. 5*C*, Table 3), while sites III and IV (K_d-app_ = 0.56 μM) (Fig. 5*D*, Table 3) bound Ca^2+^ with an approximately fivefold higher affinity. Thus, FGF12A_NTD_ and FGF12A_CaMBDp_ increased the Ca^2+^-binding affinity of CaM_N_ (Fig. 5*E*, no statistically significant difference between the effect of NTD and CaMBD) and CaM_C_ (Fig. 5*F*, with CaMBD having a larger effect by 0.25 kcal/mol). This is consistent with both domains of (Ca^2+^)_4_-CaM binding FGF12A_NTD_ exclusively through the CaMBD region and having an identical interface with both (Fig. 5*G*). This supports the interpretation of NMR data presented in Figure 4, *A*–*I* and the conclusion that the LTP does not participate in the CaM-FGF12A NTD interaction in a 1:1 complex.

**Table 3:** Effect of FGF12A_CaMBDp_ and FGF12A_NTD_ on Ca^2+^ binding of CaM

CaM_1-148_	Sites I and II			Sites III and IV		
	ΔG_1_^a^ (kcal/mol)	ΔG_2_^a^ (kcal/mol)	K_d-app_^b^ (μM)	ΔG_1_^a^ (kcal/mol)	ΔG_2_^a^ (kcal/mol)	K_d-app_^b^ (μM)
-	−6.27 ± 0.21	−12.96 ± 0.09	15.92	−6.77 ± 0.09	−15.03 ± 0.04	2.73
+ FGF12A_CaMBDp_	−7.75 ± 0.07	−16.45 ± 0.04	0.81	−8.08 ± 0.14	−17.14 ± 0.05	0.45
+ FGF12A_NTD_	−7.69 ± 0.04	−16.48 ± 0.07	0.79	−8.27 ± 0.08	−16.89 ± 0.04	0.56

Solution conditions: 50 mM HEPES, 100 mM KCl, 1 mM MgCl_2_, 50 μM EGTA, 5 mM NTA, 1 mM DTT, pH 7.4, 22 °C.

a Average ΔG values based on three independent determinations. Titrations fit to Equation 4.

b K_d-app_ (apparent dissociation constant) was calculated from half of the average ΔG_2_ value reported in this table.

### FGF12B and FGF12A differ in effects on Ca^2+^ binding by CaM+Na_V_1.2_CTD_

In isolation, both FGF12A_NTD_ and FGF12A_CaMBDp_ increased the Ca^2+^-binding affinity of CaM_N_ and CaM_C_ (Fig. 5, *A*–*D*). This suggests that the allosteric regulatory roles of FGF12A, which includes the NTD and FGF12B, will not differ because only the A-type splice variant will be capable of binding CaM. Currently there is no evidence of CaM binding to FGF12B.

To understand the complementary roles of CaM and FGF bound to Na_V_ channels, it would be ideal to determine the Ca^2+^ affinity of CaM in a ternary complex with FGF12A bound to a full-length Na_V_1.2 channel in a plasma membrane and compare that with the Ca^2+^ affinity of CaM in a complex with FGF12B bound to an identical full-length Na_V_1.2. However, no currently available method is capable of measuring this property in these large transmembrane complexes. Therefore, we investigated Ca^2+^ binding to CaM in soluble ternary complexes containing either full-length FGF12A or FGF12B bound to the Na_V_1.2_CTD_ that contains both the EFL domain and IQ motif.

Unlike the FGF12A NTD fragments, which had a low abundance of naturally occurring fluorophores (2 Phe, 0 Tyr, 0 Trp), FGF12B (8 Phe, 10 Tyr, 1 Trp), FGF12A (10 Phe, 10 Tyr, 1, Trp), and Na_V_1.2_CTD_ (9 Phe, 4 Tyr, 1 Trp) all contain multiple aromatic residues (Fig. S7*A*) that could quench or overwhelm signals coming from CaM. There is also a controversial report that the EFL of Na_V_1.5 binds Ca^2+^ (56), and that phenomenon could contribute a Ca^2+^-dependent change in fluorescence intensity coincident with that of the changes in CaM.

To determine whether the intrinsic fluorescence of FGF12B and Na_V_1.2_CTD_ was Ca^2+^-dependent, titrations of the FGF12B+Na_V_1.2_CTD_ complex were conducted. Its signal was essentially flat (Ca^2+^-independent) (Fig. S7, *B* and *C*), and its excitation (Fig. S7, *D* and *E*) and emission (Fig. S7, *F* and *G*) spectra were essentially identical under Ca^2+^-depleted conditions and in excess Ca^2+^ ([Ca^2+^]_Total_ = 10 mM). Similarly, Ca^2+^ titrations of the FGF12A+Na_V_1.2_CTD_ complex (Fig. S8*A*) showed that its fluorescence intensity was essentially flat (Fig. S8, *B* and *C*), consistent with a lack of intrinsic Ca^2+^ binding by these FGF-Na_V_ complexes. Thus, in the Ca^2+^ titrations of CaM bound to FGF12B+Na_V_1.2_CTD_ or FGF12A+Na_V_1.2_CTD_, the change in fluorescence intensity was interpreted as reporting solely on Ca^2+^ binding to CaM.

**Figure 6:**
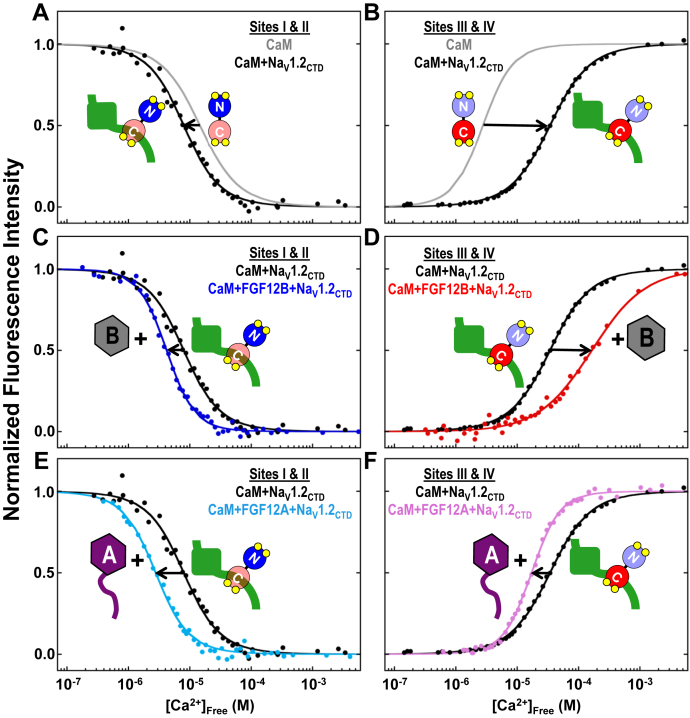
**Equilibrium Ca**^**2+**^**titrations of CaM+FGF12B+Na**_**V**_**1.2**_**CTD**_**and CaM+FGF12A+Na**_**V**_**1.2**_**CTD**._*A* and *B*, equilibrium Ca^2+^ titrations of sites I and II (*A*), and sites III and IV (*B*) of CaM in CaM+Na_V_1.2_CTD_. Solid gray lines show reference Ca^2+^ titrations sites I and II (*A*) and sites III and IV (*B*) of CaM alone. *C*, *E*, equilibrium Ca^2+^ titrations of sites I and II of CaM in CaM+Na_V_1.2_CTD_ (*C*, *E*, *black*), CaM+FGF12B+Na_V_1.2_CTD_ (*C*, *blue*) and CaM+FGF12A+Na_V_1.2_CTD_ (*E*, *teal*). *D*, *F*, equilibrium Ca^2+^ titrations of sites III and IV of CaM in CaM+Na_V_1.2_CTD_ (*D*, *F*, *black*), CaM+FGF12B+Na_V_1.2_CTD_ (*D*, *red*) and CaM+FGF12A+Na_V_1.2_CTD_ (*F*, *pink*).

To understand the effect of full-length FGF12B or FGF12A on Ca^2+^ binding by CaM bound to the Na_V_1.2_CTD_, we compared the equilibrium Ca^2+^ titrations of the ternary complexes to those of the binary CaM+Na_V_1.2_CTD_ complex (Fig. 6, *A* and *B*). In CaM+Na_V_1.2_CTD_ the Ca^2+^ affinity of CaM sites I and II (K_d-app_ = 7.91 μM, Fig. 6*A*, Table 4) increased approximately twofold compared with free CaM. This change was nearly identical to the difference observed between sites I and II in a CaM_N_ fragment (residues 1–75) and in full-length CaM and was shown to reflect the release of anticooperative interactions between CaM_N_ and residues in the linker between CaM domains (30, 57). This indicates that Na_V_1.2_CTD_ binding perturbs the thermodynamic linkage between CaM_N_ and CaM_C_.

**Table 4:** Effect of FGFB and FGF12A on Ca^2+^ binding by CaM bound to Na_V_1.2_CTD_

CaM_1-148_+ Na_V_1.2_CTD_	Ratio^a^	Sites I and II			Sites III and IV		
		ΔG_1_^b^ (kcal/mol)	ΔG_2_^b^ (kcal/mol)	K_d-app_^c^ (μM)	ΔG_1_^b^ (kcal/mol)	ΔG_2_^b^ (kcal/mol)	K_d-app_^c^ (μM)
−	−	−6.27 ± 0.39	−13.78 ± 0.08	7.91	−6.04 ± 0.19	−12.09 ± 0.07	33.42
+ FGF12B	1:1:1	−6.00 ± 0.10	−14.51 ± 0.09	4.51	−5.44 ± 0.11	−10.27 ± 0.11	157.67
+ FGF12A	1:1:1	−7.18 ± 0.22	−14.99 ± 0.08	2.82	−5.31 ± 0.07	−12.91 ± 0.10	16.61

CaM_1-148_+ Na_V_1.2_CTD_	Ratio^a^	ΔG_1-app_^b^ (kcal/mol)	ΔG_2-app_^b^ (kcal/mol)	K_d-app_^c^ (μM)	ΔG_1-app_^d^ (kcal/mol)	ΔG_2-app_^d^ (kcal/mol)	K_d-app_^c^ (μM)
+ FGF12A	2:1:1	−7.59 ± 0.14	−15.41 ± 0.14	1.97	−7.61 ± 0.24	−16.05 ± 0.04	1.14
+ FGF12A	2:1:1	–	–	–	−5.03 ± 0.16	−10.89 ± 0.13	92.94

Solution Conditions: 50 mM HEPES, 100 mM KCl, 1 mM MgCl_2_, 50 μM EGTA, 5 mM NTA, 1 mM DTT, pH 7.4, 22 °C. Fluorescence controls shown in Figures S7 and S8.

a Stoichiometry of CaM:Na_V_1.2_CTD_:FGF12B or CaM:Na_V_1.2_CTD_:FGF12B.

b Titrations fit to Equation 4. Average ΔG values based on three independent determinations monitoring Phe intensity.

c K_d-app_ (apparent dissociation constant) was calculated from half of the average ΔG_2_ value reported in this table.

d Titrations fit to Equation 5. Average ΔG based on three independent determinations monitoring Tyr intensity.

In the CaM+Na_V_1.2_CTD_ complex, the Ca^2+^ affinity of sites III and IV decreased by ∼12-fold (K_d-app_ = 33.42 μM, Fig. 6*B*, Table 4) relative to free CaM, consistent with preferential binding of the Na_V_1.2 IQ motif by apo versus (Ca^2+^)_4_-CaM (22, 23, 24, 25). Comparing the two domains of CaM to each other in the CaM+Na_V_1.2_CTD_ complex, CaM_N_ binds Ca^2+^ with a approximately fourfold higher affinity than CaM_C_, indicating that Na_V_1.2_CTD_ binding reverses the sequential occupancy of the CaM domains observed in CaM alone (gray curves in Fig. 6, *A* and *B*).

In the ternary CaM+FGF12B+Na_V_1.2_CTD_ complex, a slight additional increase (approximately twofold) was observed in the Ca^2+^ affinity of sites I and II (K_d-app_ = 4.51 μM) relative to CaM bound to the Na_V_1.2_CTD_ (Fig. 6*C*, Table 4). Inclusion of FGF12B decreased the Ca^2+^-binding affinity of sites III and IV (K_d-app_ = 157.67 μM) by approximately fivefold relative to CaM in the CaM+Na_V_1.2_CTD_ complex (Fig. 6*D*, Table 4). Thus, FGF12B binding to the Na_V_1.2_CTD_ further separated the midpoints of the Ca^2+^-binding isotherms of CaM_N_ and CaM_C_. This was an unanticipated result because there are no published reports of direct interactions between CaM and this shorter FGF12 splice variant that consists primarily of the folded β-trefoil core (Fig. 1*C*).

For CaM in CaM+FGF12A+Na_V_1.2_CTD_, the Ca^2+^ affinity of sites I and II (K_d-app_ = 2.82 μM) was more favorable than those sites in CaM+Na_V_1.2_CTD_ (approximately threefold) or CaM+FGF12B+Na_V_1.2_CTD_ (approximately twofold) (Fig. 6, *C* and *E*, Table 4). The binding of FGF12A also increased the Ca^2+^ affinity of sites III and IV (K_d-app_ = 16.61 μM) relative to CaM in both the CaM+Na_V_1.2_CTD_ (approximately twofold) and CaM+FGF12B+Na_V_1.2_CTD_ (approximately tenfold) complexes. The increased Ca^2+^-binding affinity of both CaM domains in the CaM+FGF12A+Na_V_1.2_CTD_ complex is consistent with (Ca^2+^)_4_-CaM interacting favorably with the NTD of full-length FGF12A when bound to the Na_V_1.2_CTD_.

### Binding of multiple CaM molecules to the FGF12A+Na_V_1.2_CTD_ complex

Ca^2+^-saturated CaM can bind two sites in the FGF12A NTD (Fig. 2, *A*–*L*) and one site in the Na_V_1.2 IQ motif (22, 23, 24, 25). Thus, at high local concentrations of Ca^2+^ and CaM, the CTD of Na_V_1.2 channels with FGF12A bound might bind up to three molecules of (Ca^2+^)_4_-CaM. However, (Ca^2+^)_4_-CaM binds more weakly to the LTP than the CaMBD (Fig. 2, *C* and *J*, Table 1), and the solution NMR data were consistent with (Ca^2+^)_4_-CaM binding the FGF12A_NTD_ exclusively through the CaMBD region in a one-to-one complex (Fig. 4, *E* and *I*).

**Figure 7:**
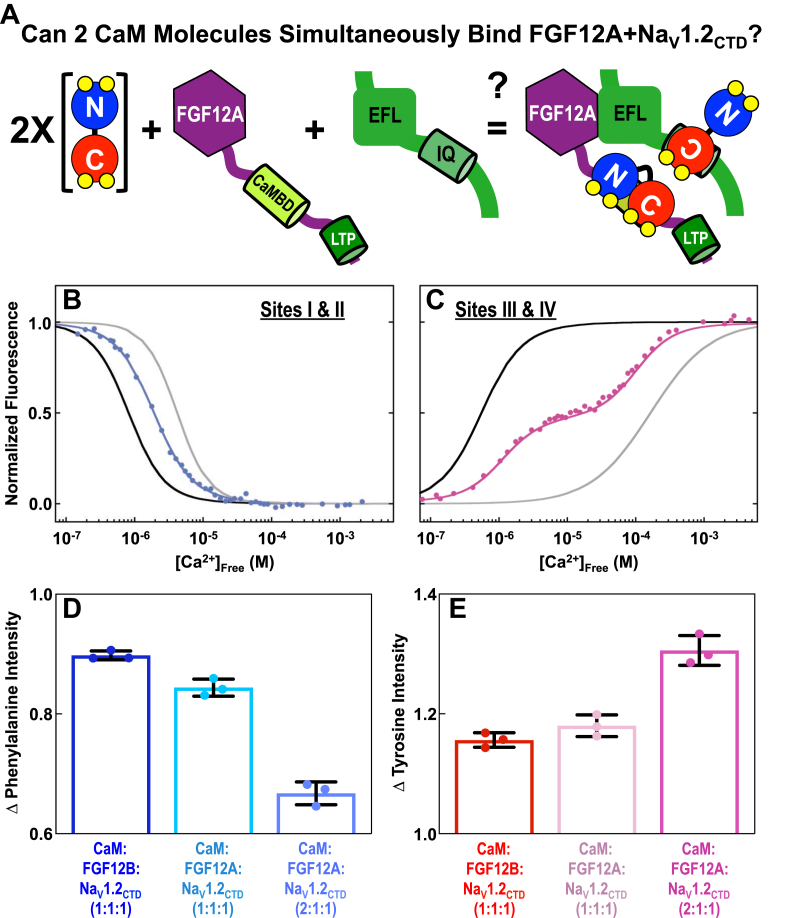
**Equilibrium Ca**^**2+**^**titrations of a 2:1:1 complex of CaM+FGF12A+Na**_**V**_**1.2**_**CTD**._*A*, schematic depicting the possible binding of two CaM (CaM_N_/*blue*, CaM_C_/*red*) molecules the FGF12A (*purple*)+Na_V_1.2_CTD_ (*green*) complex. The FGF12A LTP (*green*) and CaMBD (*limon*) are shown as *cylinders*. *B*, equilibrium Ca^2+^ titrations of CaM sites I and II in CaM+FGF12A+Na_V_1.2_CTD_ (*light blue*, CaM:FGF12A:Na_V_1.2_CTD_ = 2:1:1). The *solid black* and *gray lines* show reference titrations of CaM sites I and II in CaM+FGF12A_NTD_ and CaM+FGF12B+Na_V_1.2_CTD_, respectively. *C*, Equilibrium Ca^2+^ titrations of sites III and IV of CaM in CaM+FGF12A+Na_V_1.2_CTD_ (*hot pink*, CaM:FGF12A:Na_V_1.2_CTD_ = 2:1:1). The *solid black* and *gray lines* show reference titrations of CaM sites III and IV in CaM+FGF12A_NTD_ and CaM+FGF12B+Na_V_1.2_CTD_, respectively. *D*, maximum decrease in Phe fluorescence intensity in equilibrium Ca^2+^ titrations of CaM+FGF12B+Na_V_1.2_CTD_ (*blue*), CaM+FGF12A+Na_V_1.2_CTD_ (*teal*, CaM:FGF12A:Na_V_1.2 = 1:1:1) and CaM+FGF12A+Na_V_1.2_CTD_ (*light blue*, CaM:FGF12A:Na_V_1.2_CTD_ = 2:1:1). *E*, maximum increase in Tyr fluorescence intensity in equilibrium Ca^2+^ titrations of CaM+FGF12B+Na_V_1.2_CTD_ (*red*), CaM+FGF12A+Na_V_1.2_CTD_ (*pink*, CaM:FGF12A:Na_V_1.2 = 1:1:1), and CaM+FGF12A+Na_V_1.2_CTD_ (*hot pink*, CaM:FGF12A:Na_V_1.2_CTD_ = 2:1:1).

To explore the limits of stoichiometry, we tested whether FGF12A+Na_V_1.2_CTD_ might recruit a total of two molecules of CaM: one at the Na_V_1.2 IQ motif and one at the FGF12A CaMBD (Fig. 7*A*). To do this, Ca^2+^ titrations were conducted of the CaM+FGF12A+Na_V_1.2_CTD_ complex at a [CaM]:[FGF12A]:[Na_V_1.2_CTD_] ratio of 2:1:1 (CaM+FGF12A+Na_V_1.2_CTD_ (2:1:1)). In this complex it was anticipated that CaM_N_ would not interact with the IQ motif regardless of the Ca^2+^ concentration (25) and that it would bind the FGF CaMBD only under Ca^2+^-saturating conditions. In contrast, CaM_C_ would bind to the Na_V_1.2 IQ motif constitutively (±Ca^2+^), but CaM_C_ of a second -(Ca^2+^)_4_-CaM molecule might bind at the FGF CaMBD.

The Ca^2+^ titrations of CaM_N_ in CaM+FGF12A+Na_V_1.2_CTD_ (2:1:1) were monotonic (Fig. 7*B*, *light blue line*). Sites I and II bound Ca^2+^ with an affinity (K_d-app_ = 1.97 μM) that was between that of CaM in the CaM+FGF12A_NTD_ (K_d-app_ = 0.81 μM, Fig. 7*B*, *black line*) and CaM+FGF12B+Na_V_1.2_CTD_ (K_d-app_ = 4.51 μM, Fig. 7*B*, *gray line*, Table 4) complexes.

The Ca^2+^ titrations of CaM_C_ in the CaM+FGF12A+Na_V_1.2_CTD_ (2:1:1) were biphasic (Fig. 7*C*, *red line*), in contrast to the titrations of CaM+FGF_NTD_ (Fig. 5*B*) and CaM+FGF12A+Na_V_1.2_CTD_ with a 1:1:1 stoichiometry (Fig. 6*F*). Fitting these data as a sum of two isotherms (see Experimental procedures), we determined that the dissociation constant of the first phase (K_d-app_ = 1.14 μM) was similar to that of CaM_C_ in CaM+FGF12A_NTD_ (K_d-app_ = 0.45 μM, Fig. 7*C*, *black line*) while the dissociation constant of the second phase was similar to that of CaM_C_ in CaM+FGF12B+Na_V_1.2_CTD_ (K_d-app_ = 157.67 μM, Fig. 7*C*, *gray line*, Table 4).

Both the shape of the Ca^2+^ titration curve of CaM sites III and IV in the 2:1:1 complex and the values of the resolved dissociation constants for each transition suggest that the FGF12A+Na_V_1.2_CTD_ complex can bind two CaM molecules simultaneously. Consistent with this, the change in intensity of both the Phe (Fig. 7*D*) and Tyr (Fig. 7*E*) signals observed in titrations of the CaM+FGF12A+Na_V_1.2_CTD_ (2:1:1) complex was approximately twofold larger than in Ca^2+^ titrations of CaM+FGF12B+Na_V_1.2_CTD_ or CaM+FGF12A+Na_V_1.2_CTD_.

The biphasic titration curves of the CaM_C_ sites and the changes in affinity of CaM_N_ and CaM_C_ in the CaM+FGF12A+Na_V_1.2_CTD_ (2:1:1) complex were consistent with FGF12A+Na_V_1.2_CTD_ binding two molecules of CaM simultaneously with one molecule of CaM bound *via* the FGF12A CaMBD and the other bound at the Na_V_1.2 IQ motif. This suggests that if local concentrations of Ca^2+^ and CaM were sufficiently high, Na_V_1.2 channels that have FGF12A associated may recruit a second molecule of CaM through the FGF12A CaMBD independent of CaM binding at the Na_V_1.2 IQ motif.

## Discussion

Under resting conditions, the cytosolic CTD of Na_V_ channels binds one FGF and one CaM (16, 17, 22, 48); however, the mechanism by which these two auxiliary proteins modulate channel function is poorly understood. The thermodynamic and structural studies presented here show a direct, Ca^2+^-dependent interaction between (Ca^2+^)_4_-CaM and the NTD of the four A-type FGF splice variants. We found that (a) (Ca^2+^)_4_-CaM preferentially binds a CaMBD in the NTD with a dissociation constant in the low nM range but can bind the LTP with weaker affinity, and (b) both domains of (Ca^2+^)_4_-CaM bind the CaMBD. These results suggest that at elevated cytosolic Ca^2+^ concentrations reached during an action potential, CaM may translocate from the Na_V_ IQ motif to the FGF CaMBD and participate in regulatory functions previously identified as requiring FGF binding to Na_V_s.

### Discovery of novel (Ca^2+^)_4_-CaM-binding sites

Members of the intracellular FGF subfamily were reported to directly bind Na_V_ isoforms (17, 18), voltage-gated potassium channels (58), and islet brain protein 2 (59). A recent report proposed an interaction between CaM and FGF12B when bound to Na_V_1.4 (52); however, there has been no evidence for a direct interaction between CaM and any FGF isoform. Using multiple spectroscopic methods, we have demonstrated that the isolated LTP (Fig. 2, *B* and *C*) and CaMBD (Fig. 2, *E*–*L*) sequences of A-type FGFs bind (Ca^2+^)_4_-CaM but not apo CaM(Fig. 2, *B* and *E–H*), mirroring the selectivity of CaMBD sequences in targets such as CaMKII (60), myosin light chain kinase (61), and calcineurin (62).

**Figure 8:**
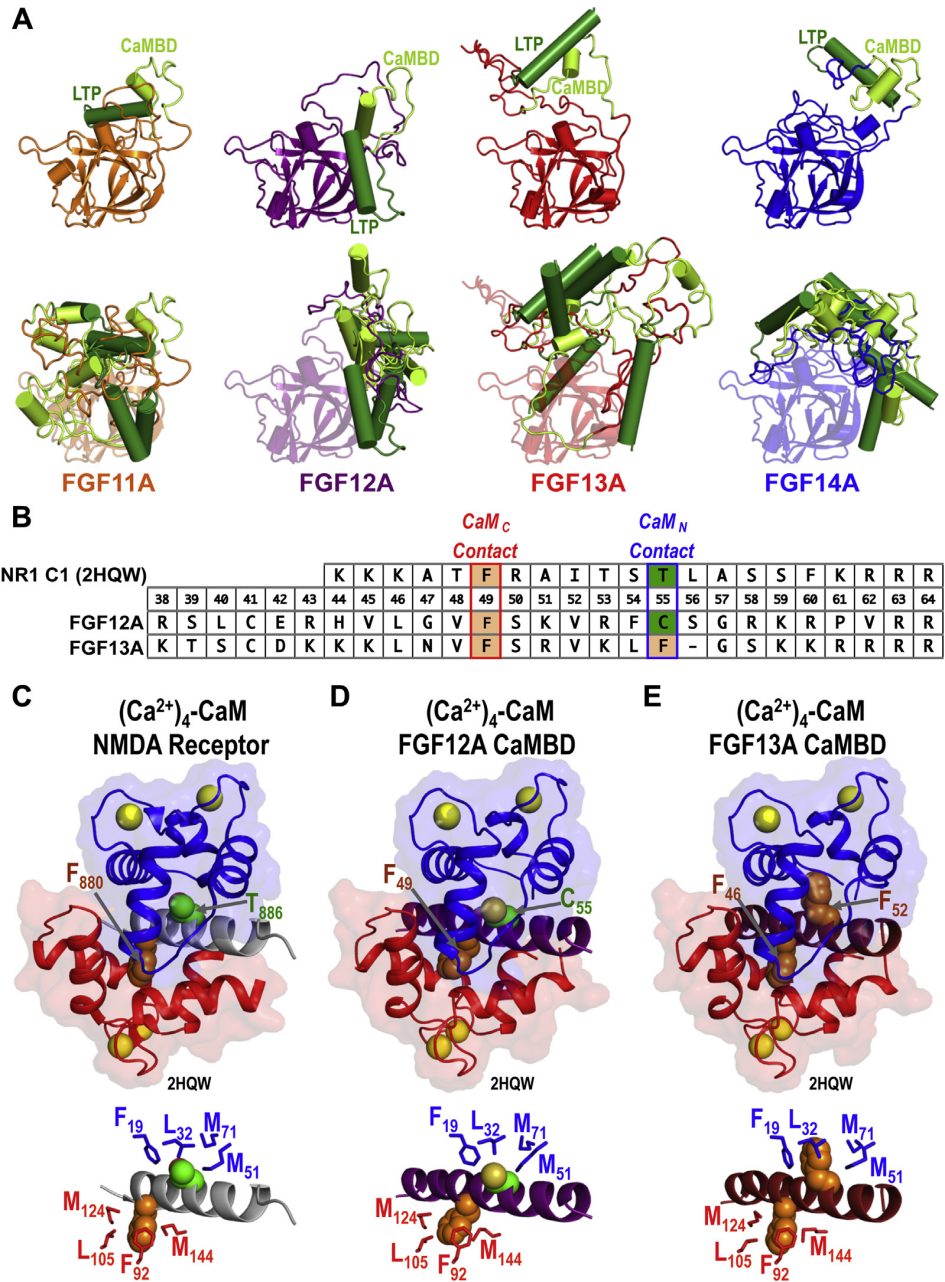
**Models of (Ca**^**2+**^**)**_**4**_**-CaM bound to the FGF12A and FGF13A CaMBD.** In all structures CaM residues 1–75 are *blue*, 76–80 are *black* and 81–148/*red*, and Ca^2+^ are *yellow spheres*. *A*, *top*, model 1 in the Robetta (54) generated ensemble of each full-length A-type FGF. *Bottom*, orientation of the LTP and CaMBD regions in the modeled ensemble of FGF11, FGF12A, FGF13A, and FGF14. The FGF isoforms are colored as follows: FGF11A is *orange*, FGF12A is *purple*, FGF13A is *red*, and FGF14A is *blue*. The LTP and CaMBD regions of each FGF are shown in *green* and *limon*, respectively. Models were aligned with FGF12A a.a. 71–204. *B*, alignment of the NMDA receptor CaMBD, FGF12A CaMBD, and FGF13A CaMBD sequences. The positions that correspond to F880 and T886in the NMDA receptor are *shaded* (hydrophobic/*tan*, polar/*green*). C: Structure of (Ca^2+^)_4_-CaM bound to the NMDA receptor CaMBD (2HQW, *gray*), F880 (*orange*), and T886 (*green*) of the NMDA receptor are shown as *spheres* (*top*) and positions of F19, L32, M51, and M71 in CaM_N_ (*blue*) and F92, L105, M124, and M144 in CaM_C_ (*red*) are shown as *sticks* relative to F880 (*orange spheres*) and T886 (*green spheres*) in the NMDA receptor CaMBD (*lower*). D: Model of (Ca^2+^)_4_-CaM bound to the FGF12A CaMBD (*purple*), F49 (*orange*), and C55 (*green*) of FGF12A are shown as spheres (*top*) and positions of F19, L32, M51, and M71 in CaM_N_ (*blue*) and F92, L105, M124, and M144 in CaM_C_ (*red*) are shown as sticks relative to F49 (*orange spheres*) and C55 (*green spheres*) in the FGF12A CaMBD (*bottom*). *E*, model of (Ca^2+^)_4_-CaM bound to the FGF13A CaMBD (firebrick), F46 and F52 of FGF13A are shown as orange spheres (*top*) and positions of F19, L32, M51, and M71 in CaM_N_ (*blue*) and F92, L105, M124, and M144 in CaM_C_ (*red*) are shown as sticks relative to F46 (*orange spheres*) and C52 (*green spheres*) in the FGF13A CaMBD (*bottom*).

Robetta models of all four full-length A-type FGFs (Fig. 8*A*) predicted that segments of both LTP and CaMBD sequences would adopt α-helical structure. For FGF12A, this was supported by the ^15^N-HSQC spectra of ^15^N-FGF12A_CaMBDp_ and ^15^N-FGF12A_NTD_ with one bound (Ca^2+^)_4_-CaM (Fig. 4, *G*–*I*, Fig S6, *C* and *D*). The energetically similar models for each FGF show an ensemble of positions for the LTP and CaMBD separated by a disordered linker. The full NTD is tethered to the β-trefoil core with a disordered linker (Fig. 8*A*). In solution, this would allow the LTP and CaMBD to move independently relative to each other and the β-trefoil core. This flexibility and range of motion may facilitate (Ca^2+^)_4_-CaM binding the FGF NTD when the β-trefoil core is bound to a Na_V_ EFL.

(Ca^2+^)_4_-CaM is known to bridge noncontiguous sites as observed in structures of CaM bound to the STRA6 retinol receptor (63) and the SK channel (64)., However, comparison of NMR spectra (Fig. 4*E*) and effects of FGF12A_CaMBDp_ and FGF12A_NTD_ on Ca^2+^ binding by CaM (Fig. 5, *A*–*F*, Table 3) were consistent with (Ca^2+^)_4_-CaM binding the FGF12A NTD exclusively at the CaMBD site. This suggests that (Ca^2+^)_4_-CaM would bind the NTD of the other A-type FGF isoforms (11A, 13A, and 14A) exclusively through the CaMBD in a one-to-one complex. However, the ability of (Ca^2+^)_4_-CaM to bind the isolated FGF12A LTP (and its nearly identical sequence in all four A-type FGFs) suggests that if the local CaM concentration is high, the LTP site could recruit a second molecule of CaM to an A-type FGF. Although (Ca^2+^)_4_-CaM binds to CaMBD with higher affinity than to LTP, CaM might interact transiently with LTP before binding to CaMBD. The reported thermodynamic studies were conducted under equilibrium conditions and did not address kinetics or explore possible translocation between the sites.

### Differences in (Ca^2+^)_4_-CaM-FGF CaMBD interface

The two-domain architecture of CaM allows it to recognize target sequences in a variety of ways, with some targets such as Na_V_ and myosin IQ motifs (65), binding a single domain of CaM (CaM_C_) while others, such as CaMKII (66), bind both. Titrations of the FGF12A and FGF13A CaMBD with E31Q/E67Q CaM (Fig. 3, *D* and *H*) and E104Q/E140Q CaM (Fig. 3, *E* and *I*), and the ^15^N-HSQC spectra of labeled (Ca^2+^)_4_-CaM bound to the unlabeled FGF12A_CaMBDp_ (Fig. 4, *B* and *C*) or FGF12A_NTD_ (Fig. 4*E*, and Fig. S5, *A–D*) were consistent with both CaM domains recognizing FGF CaMBD sequences. However, the approximately tenfold difference in the affinity between FGF13A and FGF12A indicates that their CaM–FGF interfaces differ.

To explore possible structural sources for this disparity, we modeled the (Ca^2+^)_4_-CaM– FGF12A CaMBD interface on a high-resolution structure (2HQW.pdb) of (Ca^2+^)_4_-CaM bound to the NR1C1 site of the N-methyl-D-aspartate (NMDA) receptor (67, 68). In 2HQW, F880 binds the hydrophobic cleft of CaM_C_ while T886 is the primary contact in the cleft of CaM_N_ (Fig. 8, *B* and *C*). An alignment of NR1C1 with the CaMBDs of FGF12A and FGF13A (Fig. 8*D*) suggested that CaM_C_ would bind a Phe in each FGF CaMBD (F49 in FGF12A or F46 in FGF13A) (Fig. 8, *B*–*E*). However, CaM_N_ would contact a Cys (C55) in FGF12A (Fig. 8*D*) and a Phe (F52) in FGF13A (Fig. 8*E*). In homology models of (Ca^2+^)_4_-CaM bound to the FGF12A (Fig. 8*D*) and FGF13A CaMBD (Fig. 8*E*), based on 2HQW and minimized using YASARA, the hydrophobic pocket of CaM_N_ would make more favorable close contacts with the bulkier F52 in FGF13A than with the smaller polar C55 of FGF12A. This may explain the approximately tenfold higher affinity of (Ca^2+^)_4_-CaM for FGF13A CaMBD.

The structural models shown in Figure 8, *D* and *E* were simulated assuming that CaM binds to both FGF12A and FGF13A in an antiparallel arrangement (*i.e.*, CaM_N_ binds the C-terminal half of the CaMBD) that is observed for the majority of CaM–target interactions. However, the binding of knockout mutants having only one functional domain suggests another possible arrangement. We found that Ca^2+^-saturated E104Q/E140Q CaM (functionally equivalent to apo CaM_C_ tethered to (Ca^2+^)_2_-CaM_N_) bound the FGF12A and FGF13A CaMBDs with nearly identical affinity (Fig. 3, *E*, *F*, *I*, and *J*). A simple interpretation would be that those titrations represent binding of (Ca^2+^)_2_-CaM_N_ to a similar half-site in each FGF CaMBD. Thus, the disparity in the affinity might arise from differences in the interface between CaM_C_ and FGF CaMBDs.

The ability of CaM_N_ and CaM_C_ to recognize a variety of target sequences (68) makes it extremely difficult to predict their orientation on a CaMBD. There are high-resolution structures of some peptides bound to (Ca^2+^)_4_-CaM in a parallel orientation. The ability of the hydrophobic clefts of CaM_N_ and CaM_C_ to form more favorable interactions with a Phe versus Cys may cause the functional domain in each CaM knockout mutant to compete for the same sequence in the FGF12A CaMBD, as was previously observed in the binding of (Ca^2+^)_4_-CaM to melittin (69). In that complex, the single Trp residue of melittin preferentially binds to CaM_C_ in full-length CaM, and CaM_N_ interacts elsewhere; however, CaM_N_ as an isolated fragment will bind to the Trp residue that is available because CaM_C_ is absent.

In the titrations of the FGF12A_CaMBD_ biosensor with E104Q/E140Q CaM, the functional CaM_N_ may interact with F49 in the FGF12A CaMBD. This would make the interface between E104Q/E140Q CaM with the CaMBD of either FGF12A or FGF13A similar, which could explain the nearly identical affinity of E104Q/E140Q CaM for these sequences.

Although (Ca^2+^)_4_-CaM was modeled according to the more common antiparallel orientation, CaM could recognize the FGF12A and FGF13A CaMBD in a parallel orientation as seen in structures of (Ca^2+^)_4_-CaM bound to IQ motif peptides from Ca_V_1.2, Ca_V_2.2, and Ca_V_2.3 (70, 71) and a CaMBD from CaM-dependent kinase kinase (72). Alternatively, (Ca^2+^)_4_-CaM might bind the CaMBD of FGF12A in an orientation opposite from that adopted when binding to FGF13A. Crystal structures have shown a Ca_V_ target peptide bound to CaM in both parallel and antiparallel orientations (71, 73) and TFP, an antipsychotic drug that binds (Ca^2+^)_4_-CaM, has also been found in opposing orientations (74, 75, 76).

In the future, high-resolution structures of (Ca^2+^)_4_-CaM bound to the FGF12A and FGF13A CaMBD will be required to determine the positions of CaM_N_ and CaM_C_ and how differences in the interface between CaM and these FGF CaMBDs contribute to free energies of binding.

### FGF12B lowers Ca^2+^ affinity of CaM_C_ bound to Na_V_1.2_CTD_

In high-resolution structures of apo CaM bound to Na_V_1.5 CTD fragments in the presence or absence of a B-type FGF, the interface between apo CaM_C_ and the Na_V_ IQ motif is essentially identical (28, 51). Although two residues (Y98 and K144) in the β-trefoil core of the B-type splice variant of FGF13 (FGF13U) are within 5 Å of residues K95 and N111 in CaM_C_ in a crystallographic structure of apo CaM bound to the Na_V_1.5 CTD with FGF13U (51), no interface is observed between CaM_C_ and FGF13U in this structure. The simplest conclusion is that the interaction of CaM_C_ at the Na_V_ IQ motif is independent of B-type FGF binding to Na_V_ EFL. However, binding of FGF12B to CaM+Na_V_1.2_CTD_ decreased the Ca^2+^ affinity of sites III and IV in CaM_C_ (Fig. 6*D*, Table 4). Thermodynamic linkage requires that FGF12B allosterically alters the energy of CaM binding to Na_V_1.2_CTD_.

**Figure 9:**
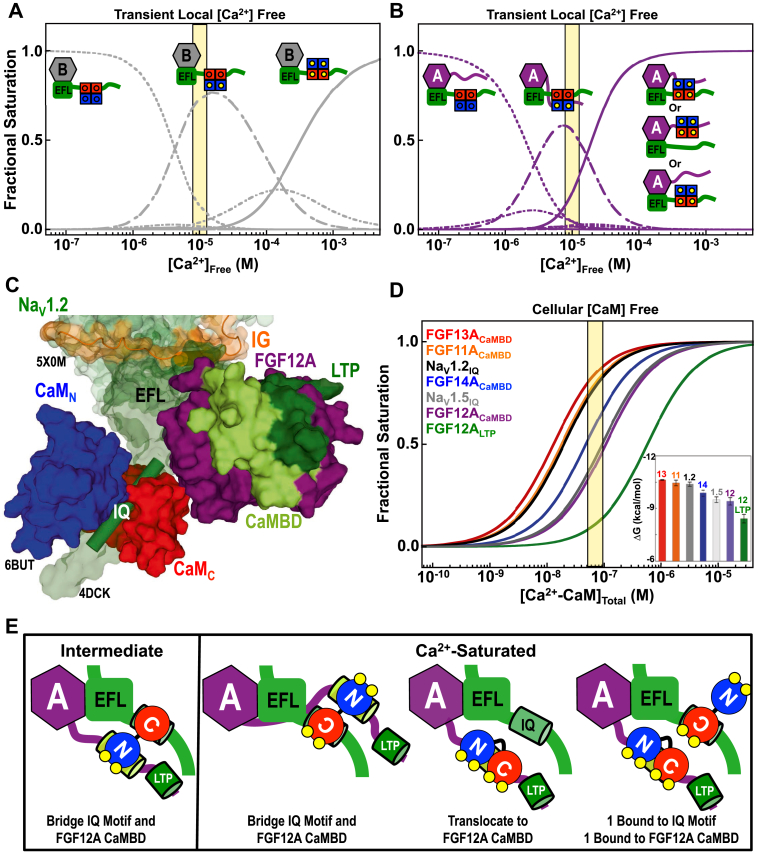
**States of CaM bound to FGF12B+Na**_**V**_**1.2**_**CTD**_**or FGF12A+Na**_**V**_**1.2**_**CTD.**_*A* and *B*, abundance of ligated CaM species in the CaM+FGF12B+Na_V_1.2_CTD_ (*A*, *gray*) and CaM+FGF12A+Na_V_1.2_CTD_ (*B*, *purple*) complexes. Simulations are based on the Ca^2+^-binding affinities reported in Table 4. States of CaM with an abundance >0.25 are shown in the inset schematics that depict the possible orientations of the CaM domains (CaM_N_/*blue*, CaM_C_/*red*) relative to FGF12B (*gray*) and Na_V_1.2_CTD_ (*green*) (*A*) or FGF12A (purple) and Na_V_1.2_CTD_ (*green*) (*B*), Ca^2+^ are shown as *yellow circles*. The *yellow box* is centered at physiologically relevant Ca^2+^ concentration of 10 μM. Representing the occupancy of the four Ca^2+^-binding sites in CaM (in order I, II, III, IV) with binary, where 0 is emtpy and 1 if filled, the discernible (abundances >0.05) species of CaM in the CaM+FGF12B+Na_V_1.2_CTD_ complex (*A*) are 0000 (*dotted line*), 1100 (*dashed* and *dotted line*), 1101 or 1110 (*dashed line*), and 1111 (*solid line*). The discernible (abundance >0.5) species of CaM in the CaM+FGF12A+Na_V_1.2_CTD_ complex (*B*) are 0000 (*dotted line*), 1000 or 0100 (*dashed line*), 1100 (*dashed* and *dotted line*), 1111 (*solid line*). *C*, model of CaM (CaM_N_/*blue*, CaM_C_/*red*) bound to an Na_V_ CTD (*forest green*, surface) relative to the Na_V_ DIII-DIV linker (*orange*) and FGF12A (*purple*, LTP/*green*, CaMBD/*limon*). The model is a composite of Na_V_1.5 CTD (4DCK), aligned with Na_V_PAS (5X0M) EFL (a.a. 1426–1521). Na_V_PAS a.a. 1122–1160 are shown as the DIII–DIV linker. The apo CaM+Na_V_1.2_IQp_ ensemble (6BUT) was aligned with 4DCK via CaM a.a. 101–112 and 117–128. The Na_V_1.2_IQp_ (a.a. 1904–1924) is shown as a *forest green cylinder*. The model of FGF12A was aligned with 4DCK using FGF13U a.a. 11–158. For clarity CaM and FGF13B in 4DCK are hidden. *D*, simulation of the saturation of each A-type FGF CaMBD (FGF11A/*orange*, FGF12A/*purple*, FGF13A/*red*, FGF14A/*blue*), FGF12A LTP (*green*) or the IQ motifs of Na_V_1.2 (*black*) and Na_V_1.5 (*gray*) with (Ca^2+^)_4_-CaM. Simulations of (Ca^2+^)_4_-CaM binding the FGF12A LTP and each FGF CaMBD are based on the affinities reported in Table 1. Simulations of (Ca^2+^)_4_-CaM binding to IQ motifs are based on K_d_ of 19.8 nM for Na_V_1.2 and 92.3 nM for Na_V_1.5 (data not shown). The yellow shaded region highlights the estimated concentration range of free CaM (90) within the cell. The *inset* shows the ΔG of (Ca^2+^)_4_-CaM binding to the putative A-type FGF CaMBDs (FGF11A/*orange*, FGF12A/*purple*, FGF13A/*red*, and FGF14/*blue*), FGF12A LPTD (*green*) and the Na_V_1.2 IQ motif (*black*) and Na_V_1.5 IQ motif (*gray*). The affinities are arranged from tightest (*left*) to weakest (*right*). *E*, schematic depiction of different models of partially ((Ca^2+^)_2_-CaM_N_, apo CaM_C_) and fully Ca^2+^-saturated CaM (CaM_N_/*blue*, CaM_C_/*red*) binding to the IQ motif (*green cylinder*) in Na_V_1.2_CTD_ (*green*) and/or FGF12A CaMBD (*limon cylinder*) in full-length FGF12A (*purple*). The FGF12A LTP is shown as a *green cylinder* and Ca^2+^ is shown as *yellow circles*.

Because CaM is a highly acidic protein (pI ∼ 4) and FGF12B is basic (pI ∼ 9), we hypothesized that FGF12B may increase the affinity of apo CaM for the Na_V_1.2_CTD_ through favorable electrostatic interactions. However, in superpositions of apo and (Ca^2+^)_4_-CaM bound to the Na_V_1.2_CTD_ with either FGF12B or FGF12A (Fig. 9*C* and Fig. S9, *A*–*D*), the nearest residues in apo CaM_C_ (K94) or (Ca^2+^)_2_-CaM_C_ (I130 and R126) and the large basic patch in the β-trefoil core of FGF12B (K117) or FGF12A (K193) are separated by > 13 Å. Thus, it is unlikely that electrostatic interactions between CaM_C_ and FGF12B are sufficient to explain the FGF12B-induced changes in the energetics of CaM association with the Na_V_1.2_CTD_.

Ca^2+^ binding to CaM_C_ induces a ∼180° rotation of CaM_C_ on the Na_V_1.2 IQ motif (25). This may require transient release and reassociation of CaM_C_ with the Na_V_1.2 IQ motif, which in turn might be facilitated by conformational flexibility (*i.e.*, a hinge) between the EFL and IQ motif of Na_V_1.2. In crystallographic structures containing an Na_V_ CTD bound by FGF12B or FGF13U, the FGF contacts both the Na_V_ EFL and residues that precede the IQ motif (26, 51). This suggests that the binding of FGF12B may alter the dynamics between the Na_V_1.2 EFL and IQ motif, which could perturb the ability of CaM_C_ to reassociate with the Na_V_1.2 IQ motif following a Ca^2+^-induced release.

### Ca^2+^ ligation states of CaM bound to Na_V_1.2

Based on the Ca^2+^ affinities of the CaM domains when bound to FGF12B+Na_V_1.2_CTD_ or FGF12A+Na_V_1.2_CTD_, we predict that, at high cytosolic Ca^2+^ concentrations (∼10 μM (77)), Na_V_1.2 associated with FGF12B will have either apo CaM or half-saturated ((Ca^2+^)_2_-CaM_N_, apo CaM_C_) CaM bound (Fig. 9*A*). In contrast, channels bound to FGF12A are expected to be populated by apo, half ((Ca^2+^)_2_-CaM_N_, apo CaM_C_), and fully Ca^2+^-saturated CaM (Fig. 9*B*). Thus, Na_V_1.2 with either FGF12 splice variant bound could undergo regulatory processes requiring only apo CaM or Ca^2+^ binding solely to the sites in CaM_N_. However, only Na_V_1.2 bound to FGF12A would support modulation requiring Ca^2+^-induced rotation or release of CaM from the Na_V_1.2 IQ motif.

The effect of FGF binding on Na_V_ function has been shown to depend upon the FGF splice variant bound (21, 43, 46, 47). Multiple splice variants of a particular FGF isoform have been found to be expressed simultaneously (38, 78), suggesting that within a cell, Na_V_s would be associated with more than one. Their unique effects on Na_V_ function have been proposed to correlate with variations in their NTD sequences. While it is unclear how CaM and the FGFs modulate Na_V_ function, the FGF splice variant-dependent differences in the available states of CaM during a spike in the local Ca^2+^ concentration could contribute to their unique effects on the functional states of Na_V_.

### Cellular competition for (Ca^2+^)_4_-CaM

Both CaM and FGF colocalize with Na_V_ (19, 47, 50, 79, 80). Because both apo and (Ca^2+^)_4_-CaM bind Na_V_ IQ motifs with high affinity (22, 23, 24, 25, 26, 27, 28) and the β-trefoil cores of FGF12 and FGF13 also bind Na_V_ CTD fragments with high affinity (20, 40), it is likely that CaM and an FGF are constitutively associated with an Na_V_ (Fig. 9*C*). Proteomic studies investigating Na_V_1.2-associated proteins in rat neurons have found a level of CaM and FGF enrichment in pull-downs of the Na_V_1.2 α-subunit similar to other constitutively associated proteins such as the Na_V_ β-subunit β2 (16).

Our thermodynamic and structural studies suggest that at elevated cytosolic Ca^2+^ levels, an FGF CaMBD and Na_V_ IQ motif will compete for (Ca^2+^)_4_-CaM in an isoform-dependent manner (Fig. 9*D*) due to their proximity and similar affinities for (Ca^2+^)_4_-CaM. The FGF12A-induced increase in the Ca^2+^ affinity of both CaM domains when bound to the Na_V_1.2_CTD_ (Fig. 6, *E* and *F*) suggests that CaM translocates from the Na_V_ IQ motif to the FGF CaMBD at elevated cytosolic Ca^2+^ concentrations.

While the nine Na_V_ and four FGF isoforms have tissue-dependent patterns of expression, multiple isoforms of both are expressed in some tissues (38, 42, 45, 81, 82), implying that multiple Na_V_-FGF pairings are present across tissues as well as within a particular cell. The different possible forms of FGF CaMBD and Na_V_ IQ motif recognition by (Ca^2+^)_4_-CaM may have distinct effects on channel function and could provide a means to elicit unique modulation of Na_V_ by Ca^2+^, CaM, and FGF in different tissues and potentially across channels within a particular cell. Structural and functional studies investigating (Ca^2+^)_4_-CaM bound to complexes composed of CTD fragments of other Na_V_ isoforms and FGF11A, FGF13A or FGF14A will be needed to determine the generality of the allosteric effects of FGFs on Ca^2+^ binding by CaM observed in this study

### Stoichiometry of CaM bound to Na_V_

The Ca^2+^ titrations of CaM+FGF12A+Na_V_1.2_CTD_ (2:1:1) were consistent with the FGF12A+Na_V_1.2_CTD_ complex binding one molecule of CaM at the FGF12A CaMBD and one at the Na_V_1.2 IQ motif (Fig. 7, *B*–*D*, Table 4). This may allow Na_V_ channels to recruit a second molecule of CaM, as has been reported for Ca_V_1.2 (50, 83). The ability of the FGF CaMBD to anchor a CaM molecule to an Na_V_ may explain the results of a recent report that found FGF13A is sufficient to regulate arrhythmogenic late current in cardiac Na_V_1.5 channels with an IQ>AA mutation that blocks CaM binding (44). Although those channels would not have CaM bound at the IQ motif, our findings predict that the NTD of FGF13A would bind (Ca^2+^)_4_-CaM, which might be sufficient for regulation if the primary role of the IQ motif is to serve as a sink of constitutively bound CaM.

The closely related channel Na_V_1.4, primarily found in skeletal muscle, bound only a single molecule of CaM in HEK293 cells under resting conditions and during spikes in cytosolic Ca^2+^ (50). That 1:1 stoichiometry may be related to the fact that HEK293 cells do not express any A-type FGF splice variant (42) and may lack (or have a lower expression level of) other auxiliary proteins as well. When present, other auxiliary proteins could enable multiple CaM molecules to be recruited to an Na_V_ in an excitable cell.

The schematic models in Figure 9*E* show Na_V_1.2_CTD_ with both CaM and FGF12A bound to illustrate how partially ((Ca^2+^)_2_-CaM_N_, apo CaM_C_) and fully Ca^2+^-saturated CaM may recognize the Na_V_1.2 IQ motif and FGF12A CaMBD and how two CaM molecules might bind. The stoichiometry between CaM, Na_V_1.2_CTD_, and FGF12A and locations of CaM reflect those used in the Ca^2+^ titrations of the CaM+FGF12A+Na_V_1.2_CTD_ complex and the results of those experiments (Fig. 6, *E* and *F*, and Fig. 7, *A–E*). However, the finding that (Ca^2+^)_4_-CaM binds the isolated FGF12A LTP with a weaker affinity than the CaMBD suggests that at a sufficiently high local CaM concentration a third CaM molecule could also bind to the LTP.

Multiple reports have found that (Ca^2+^)_4_-CaM binds the highly conserved linker between domains III and IV (DIII–DIV linker) (56, 84, 85, 86, 87). Recently CaM has also been reported to associate with a site in the NTD of Na_V_1.5 (88). The direct binding of (Ca^2+^)_4_-CaM to the LTP and CaMBD in the NTD of the A-type FGFs suggests that, when bound to an A-type FGF, the CTD of an Na_V_ may associate with three molecules of CaM simultaneously. Thus, in the cell an Na_V_ with a bound A-type FGF could potentially bind multiple CaM molecules.

Within the context of a cell, multiple targets compete for CaM, making free CaM a limiting reagent in the cytosol (89, 90). However, a number of CaM-binding proteins are found near or within the membrane that may serve as sinks. Among these proteins are ion channels including the Na_V_, Ca_V_, SK channels, and NMDA receptor, which can form clusters in the membrane (91, 92, 93) that would have high local CaM concentrations. Additional CaM-binding targets, such as neurogranin (94, 95) and neuromodulin (96), constitutively bind apo CaM and release it upon Ca^2+^ binding. This may provide a pool of free CaM on the intracellular side of the plasma membrane that could allow recruitment of additional CaM to an Na_V_ associated with an A-type FGF.

Flux of Na^2+^ through the pore of the Na_V_ α-subunit is tightly regulated by a network of intramolecular conformational changes and direct interactions with auxiliary proteins that are expressed as different isoforms and splice variants. These may interact with each other as well as with the channel, which could contribute to how they tune Na_V_ function. An understanding of the structural and energetic forces driving direct interactions between individual auxiliary proteins present at an Na_V_ will provide valuable insights into how they work in concert to modulate channel function as well as how mutations within their sequences disrupt regulatory processes.

The thermodynamic and structural studies here are the first to identify a direct Ca^2+^-dependent interaction between CaM and the NTD of A-type FGFs and suggest that Ca^2+^-saturated CaM may translocate from the Na_V_ IQ motif to the FGF CaMBD. These findings lay the groundwork for future studies investigating the consequence of the (Ca^2+^)_4_-CaM–FGF CaMBD interaction on Na_V_ function.

## Experimental procedures

### Modeling software and protein structure images

Model structures of human full-length FGF11A (UniProt ID: Q92914), FGF12A (UniProt ID: P61328), FGF13A (UniProt ID: Q92913), and FGF14A (UniProt ID: Q92915) were generated with the Robetta server (https://robetta.bakerlab.org/) (54). Helical wheels of the FGF12A LTP (aa 1–23) and CaMBD (38–64) were made with HeliQuest (https://heliquest.ipmc.cnrs.fr/) (97). PyMOL (Schrödinger LLC) was used to render all protein structures with these color conventions (unless stated otherwise): CaM residue 1–75/blue, 76–80/black, 81–148/red, Ca^2+^/yellow, Na_V_/green, FGF12B/light gray, FGF12A/deep purple, FGF12A LTP/forest green, and FGF12A CaMBD/limon, and calculate vacuum electrostatic surfaces.

### Protein expression and purification

Full-length (aa 1–148) and domain fragments (CaM_N_ [aa 1–75], CaM_C_ [aa 76–148]) of WT or mutant human CaM sequences were bacterially overexpressed and purified (23, 98). Genes for full-length or domain fragments of WT or mutant CaM sequences [E31Q/E67Q (E32Q/E68Q) and E104Q/E140Q (E105Q/E141Q)] were expressed with a pT7-7 vector (23). The standard protein designation for each mutant is given first. The parenthetical notation corresponds to the UniProt convention in which the initial Met is designated as residue 1. The three human genes for CaM (CALM1, CALM2, and CALM3) all code for the same protein sequence that corresponds to the sequence of WT CaM used in this study.

The FGF12A_CaMBDp_ (aa 41–70, [CERHVLGVFSKVRFCSGRKRPVRRRPEPQL]), FGF11A_NTD_ (aa 1–68) FGF12A_NTD_ (aa 1–70), FGF13A_NTD_ (aa 1–66), FGF14A_NTD_ (aa 1–68), full-length FGF12A (aa 1–243), full-length FGF12B (aa 1–181), and Na_V_1.2_CTD_ (aa 1777–1937) were expressed with an N-terminal His-GST-tag using a pBG101 vector (99). The FGF12A_CaMBDp_, FGF11A_NTD_, FGF12A_NTD_, FGF13A_NTD_, FGF14A_NTD_, FGF12A, FGF12B, and Na_V_1.2_CTD_ constructs contained four nonnative residues (GPGS) at the N terminus after removal of the His-GST-tag with 3C protease. FGF12B and complexes of full-length CaM bound to human FGF12A_CaMBD_, FGF11A_NTD_, FGF12A_NTD_, FGF13A_NTD_, FGF14A_NTD_, or human Na_V_1.2_CTD_ (SCN2A) were made by bacterial coexpression and purified as previously described (100). Purity was assessed by SDS-PAGE, UV/Vis spectroscopy, and rpHPLC.

### Purification and refolding of full-length FGF12A from inclusion bodies

The cell pellet was thawed, resuspended in lysis buffer (50 mM Tris, 500 mM KCl, 0.01% (w/v) NaN_3_, 1 mM DTT, 1% (v/v) Triton X-100, pH = 7.4), sonicated, and centrifuged (15,000 rpm, 4 °C, 20 min). The supernatant was discarded, and the pellet was stored at –80 °C. Inclusion bodies were solubilized for 2 h at 22–25 °C, with gentle rocking, in 50 mM Tris, 100 mM KCl, 5 mM imidazole, 6 M guanidine HCl, 1 mM DTT, 0.01% (w/v) NaN_3_, pH 7.4. The sample was then centrifuged (25,000 rpm, 4 °C, 25 min), the supernatant passed through a sterile 0.45 μM PVFD filter and loaded onto 5 ml of nickel sepharose resin (GE Life Sciences) equilibrated in wash buffer (50 mM Tris, 100 mM KCl, 20 mM imidazole, 6 M urea, 1 mM DTT, 0.01% (w/v) NaN_3_, 1 mM DTT, pH 7.4). The resin was then washed with 25 ml of wash buffer, and the His-GST-tagged FGF12A was eluted with elution buffer (wash buffer with 500 mM imidazole).

Full-length FGF12A was then refolded by rapid dilution into refolding buffer (100 mM Tris, 200 mM KCl, 100 mM L-arginine, 5% (w/v) sucrose, 0.02% NaN_3_, 2 mM DTT, pH 7.7, 500 ml per L of cell growth) at 4 °C stirred at 700 rpm with a Teflon-coated stir bar. Prior to the addition of the denaturated His-GST-FGF12A, the CaM+Na_V_1.2_CTD_ complex (final concentration 0.2 μM) was added to the refolding buffer to assist FGF12A refolding. Twenty-four hours after the addition of His-GST-FGF12A, insoluble material was removed by centrifugation (20,000 rpm, 4 °C, 20 min) and the sample was concentrated. The HIS-GST-tag was cleaved with 3C protease and removed by repassing the sample over nickel sepharose resin. Anion exchange chromatography (pH 8.5–7.4, KCl 0–300 mM) separated FGF12A and Na_V_1.2_CTD_ from CaM to make the FGF12A+Na_V_1.2_CTD_ complex. Complex purity (>95%) was assessed by SDS-PAGE, rpHPLC, and UV-Vis spectroscopy.

### Affinity of CaM for FGF biosensors

YFP-CFP biosensors containing the sequences for WT human FGF11A CaMBD (aa 36–62), FGF12A LTP (aa 1–23), FGF12A CaMBD (aa 38–64), FGF13A CaMBD (aa 35–60), or FGF14A CaMBD (aa 37–63) were expressed from a pET21B vector (25) (parent vector from A. Persechini and D.J. Black (UMKC) (101, 102)). The FGF sequence used in each biosensor is given in Table 5. Biosensors (≥1 nM) were titrated with WT or mutant CaM (50 mM HEPES, 100 mM KCl, 1 mM MgCl_2_, 50 μM EGTA, 5 mM NTA, 1.5 μM BSA, 500 μM DTT, 1 mM CaCl_2_, pH 7.4, 22 °C) in a stirred, water-jacketed quartz cuvette. The steady-state fluorescence intensity of CFP (λ_EX_ 430 nm, λ_EM_ 475 nm), YFP (λ_EX_ 430 nm, λ_EM_ 525 nm), and an experimentally determined isoemissive point (λ_EX_ 430 nm, λ_EM_ 509–513 nm) were monitored throughout the titration with a PTI QM4 fluorimeter (4 nm excitation, 8 nm emission bandpasses, 4–16 s integration). Emission spectra (λ_EX_ 430 nm, 4 nm excitation, 8 nm emission bandpasses, 4 s integration) were collected from 450 to 550 nm.

**Table 5:** FGF biosensor sequences

FGF_LTP_	Start Position^a^	Sequence^b^	End Position^a^	pI^c^
FGF12A_LTP_	1	MAAAIASSLIRQKRQARESNSDR	23	11.54


FGF_CaMBD_	Start Position^a^	Sequence^b^	End Position^a^	pI^c^
FGF11A_CaMBD_	36	KSLCQKQLLILLSKVRLCGGRPARPDR	62	10.95
FGF12A_CaMBD_	38	RSLCERHVLGVFSKVRFCSGRKRPVRR	64	11.88
FGF13A_CaMBD_	35	KTSCDKNKLNVFSRVKLFGSKKRRRR	60	11.85
FGF14A_CaMBD_	37	KTSCDKNKLNVFSRVKLFGSKKRRRR	63	11.85

a Start and end positions are numbered according to the UniProt (https://www.uniprot.org/) convention (106).

b FGF sequence inserted between the sequences for the YFP and CFP fluorophores in the FGF biosensor constructs (at KpnI and AgeI restriction sites).

c Theoretical isoelectric point calculated with ExPASy (https://web.expasy.org/protparam/) (107).

Buffer subtracted and dilution-corrected titrations were fit to Equation 1, as described (25).

(1) $$\bar{Y}=\frac{K_{a}{\left[CaM\right]}_{free}}{1+K_{a}{\left[CaM\right]}_{free}}$$

where *[CaM]*_*free*_ was calculated using Equation 2,

(2) $${\left[CaM\right]}_{free}=\frac{-b\pm \sqrt{b^{2}-4K_{a}\left(-{\left[CaM\right]}_{total}\right)}}{2K_{a}}$$

*b* is (1 + *K*_*a*_*·[biosensor]* − *K*_*a*_*·[CaM]*_*total*_), and the positive value is taken as *[CaM]*_*free*_. The quality of each fit was judged by evaluating the values of the 67% confidence intervals for each parameter, the span and randomness of the residuals, square root of the variance, and the values of the correlation matrix (30). The magnitude of the confidence intervals was typically smaller and within a factor of 2 of the standard deviation of the average determined from the independent replicate titrations. The average ΔG values and standard deviations from three to nine replicate titrations are reported in Tables 1 and 2. Pairwise comparisons were evaluated using the unpaired *t*-test (GraphPad Prism, StatPlus); all *p* values were considered to be very (<0.005) or extremely (<0.0001) statistically significant unless noted otherwise.

### Molar ratio of CaM to FGF NTD

The molar ratio of CaM to FGF NTD in the copurified complexes of (Ca^2+^)_4_-CaM bound to the FGF11A_NTD_, FGF12A_NTD_, FGF13A_NTD_, or FGF14A_NTD_ was analyzed chromatographically with rpHPLC. The complexes were separated with a Supelco C-18 column with a binary solvent system of water (A) and acetonitrile (B), both with 0.1% (v/v) TFA, using the following gradients: 20%–70% B from 1 to 10 min, 70% B from 10 to 14 min, 70%–90% B from 14 to 16 min. The molar ratio between CaM and the FGF11A_NTD_, FGF12A_NTD_, FGF13A_NTD_, or FGF14A_NTD_ was determined by comparing the area of the absorbance peaks at 220 nm for CaM and the FGF NTD construct in rpHPLC chromatograms.

### Solution NMR

Spectra were collected at 25 °C on a Bruker Avance II 500 MHz, Varian Unity Inova 600 MHz, or cryoprobe-equipped Bruker Avance NEO 600 MHz spectrometer. Samples were ^15^N-(Ca^2+^)_2_-CaM_N_ (450 μM), ^15^N-(Ca^2+^)_2_-CaM_C_ (300 μM), ^15^N-(Ca^2+^)_4_-CaM+^14^N-FGF12A_CaMBDp_ (330 μM), ^14^N-(Ca^2+^)_4_-CaM+^15^N-FGF12A_CaMBDp_ (450 μM), ^15^N-(Ca^2+^)_4_-CaM+^14^N-FGF12A_NTD_ (290 μM), or ^14^N-(Ca^2+^)_4_-CaM+^15^N-FGF12A_NTD_ (600 μM) in 50 mM HEPES, 100 mM KCl, 1 mM MgCl_2_, 50 μM EGTA, 5 mM NTA, 0.01% NaN_3_, 10 mM CaCl_2_, ±1 mM DTT, pH 7.4. Spectra were processed with NMRPipe (103) and analyzed with CCPN Analysis (104). SPARTA+ (Shifts Predicted from Analogy in Residue-type and Torsion Angle) (55) was used to predict the position of peaks for residues 41–70 (FGF12A_CaMBDp_) and 1–70 (FGF12A_NTD_) of model 1 in the ensemble of full-length FGF12A generated with Robetta (54).

### Steady-state fluorescence excitation and emission spectra

Excitation (λ_EM_ 280 nm, λ_EX_ 240–270 nm or λ_EM_ 320 nm, λ_EX_ 250–310 nm) and emission (λ_EX_ 250 nm, λ_EM_ 270–370 nm or λ_EX_ 277 nm, λ_EM_ 290–370 nm) spectra of Na_V_1.2_CTD_ (4 μM) and FGF12B (5 μM) (50 mM HEPES, 100 mM KCl, 1 mM MgCl_2_, 50 μM EGTA, 5 mM NTA, 500 μM DTT, ± 10 mM CaCl_2_, pH 7.4, 22 °C) were collected in a stirred, water-jacketed quartz cuvette with a PTI QM4 fluorimeter. Spectra of were collected with 3 (excitation: λ_EM_ 320 nm, λ_EX_ 250–310 nm, emission: λ_EX_ 277 nm, λ_EM_ 290–370 nm) or 5 nm (excitation: λ_EM_ 280 nm, λ_EX_ 240–270 nm, emission: λ_EX_ 250 nm, λ_EM_ 270–370 nm) bandpasses and a 4 s integration time.

### Equilibrium Ca^2+^ titrations

Equilibrium Ca^2+^ titrations of full-length WT CaM (4 μM) alone or with the FGF12A_CaMBDp_ (4 μM), FGF12A_NTD_ (4 μM), Na_V_1.2_CTD_ (4 μM), FGF12B (5 μM) and Na_V_1.2_CTD_ (4 μM), or FGF12A and Na_V_1.2_CTD_ (4 μM) in 5 nM Oregon Green or 50 nM XRhod-5F, 50 mM HEPES, 100 mM KCl, 1 mM MgCl_2_, 50 μM EGTA, 5 mM NTA, 500 μM DTT, pH 7.4, 22 °C were conducted in a water-jacketed quartz cuvette as described (23). Ca^2+^ titrations of the CaM+FGF12A+Na_V_1.2_CTD_ complex conducted at a CaM:FGF12A:Na_V_1.2_CTD_ ratio of 2:1:1 were conducted with 8 μM CaM and 4 μM FGF12A+Na_V_1.2_CTD_ in the same buffer. Steady-state fluorescence intensity of intrinsic CaM fluorophores, Phe (λ_EX_ 250 nm, λ_EM_ 280 nm) and Tyr (λ_EX_ 277 nm, λ_EM_ 320 nm), was monitored with a PTI QM4 fluorimeter. Phe and Tyr-monitored titrations of CaM alone, +FGF12A_CaMBDp_, or +FGF12A_NTD_ were collected with 3 nm bandpasses and a 4 s integration time. Phe-monitored titrations of the CaM+Na_V_1.2_CTD_, +FGF12B+Na_V_1.2_CTD_ or +FGF12A+Na_V_1.2_CTD_ were collected using 5 nm bandpasses and a 16 s integration time, and Tyr-monitored Ca^2+^ titrations of these complexes were collected with 3 nm bandpasses and a 16 s integration time. The free [Ca^2+^] was calculated from the change in Oregon Green (λ_EX_ 494 nm, λ_EM_ 521 nm, K_d_ 34.2 μM) or XRhod-5F (λ_EX_ 576 nm, λ_EM_ 603 nm, K_d_ 1.78 μM) emission intensity (31). The average affinity and standard deviation from three to four replicate titrations are reported in Tables 3 and 4.

### Analysis of Ca^2+^-binding affinity

Each domain of CaM can be considered a two-site macromolecule and the affinities of Ca^2+^ binding to sites I and II or sites III and IV can be fit to a two-site Adair equation (Equation 3) (23)

(3) $${\bar{Y}}_{total}={\bar{Y}}_{2}=\frac{K_{1}\left[X\right]+2K_{2}{\left[X\right]}^{2}}{2\left(1+K_{1}\left[X\right]+K_{2}{\left[X\right]}^{2}\right)}$$

where *K*_*1*_ (*ΔG*_*1*_ = −*RTlnK*_*1*_) is the sum of the intrinsic constants (*k*_*1*_ + *k*_*2*_) of the two sites in either domain of CaM, *K*_*2*_ (*ΔG*_*2*_ = −*RTlnK*_*2*_) is the product of the intrinsic constants and the cooperativity constant (k_1_·k_2_·k_c_), and [X] is the free [Ca^2+^].

To account for variations in intensity between experiments, the low and high endpoints of each titration were fit to the function *[f(x)]* shown in Equation 4 with nonlinear least squares analysis.

(4) $$f\left(X\right)=Y_{\left[X\right]low}+{\bar{Y}}_{2}\cdot Span$$

The variable Y_[X]low_ corresponds to the intensity of the Ca^2+^-depleted sample, $\bar{Y}$_2_ is the average fractional saturation, and Span accounts for the difference in intensity at the highest and lowest ligand concentrations. Ca^2+^ titrations of CaM in the presence of the FGF12A_CaMBDp_, FGF12_NTD_, Na_V_1.2_CTD_, FGF12B and Na_V_1.2_CTD_, or FGF12A and Na_V_1.2_CTD_ were analyzed assuming that each domain of CaM contained two functional Ca^2+^-binding sites. Pairwise comparisons of ΔG determinations were evaluated using the unpaired *t* test (GraphPad Prism, StatPlus); all *p* values were considered to be very (<0.005) or extremely (<0.0001) statistically significant unless noted otherwise.

### Analysis of Ca^2+^-binding affinity of CaM+FGF12A+Na_V_1.2_CTD_

Ca^2+^ titrations of CaM sites I and II in the CaM+FGF12A+Na_V_1.2_CTD_ complex conducted at a [CaM]:[FGF12A]:[Na_V_1.2_CTD_] ratio of 2:1:1 were fit to Equation 5. Ca^2+^ titrations of CaM sites III and IV in the same complex were fit to the biphasic function [f(x)] shown in Equation 5 as previously described (37).

(5) $$f\left(X\right)=\bar{Y_{2A}}\cdot Span_{A}+\bar{Y_{2B}}\cdot Span_{B}+Y_{\left[X\right]low}$$

The variables $\bar{Y}$_2A_ and $\bar{Y}$_2B_ are the average fractional saturation of the Ca^2+^-binding sites that correspond to the first and second transitions, respectively. Span_A_ and Span_B_ account for the direction and magnitude of signal change in the first and second transitions, respectively, and Y_[X]low_ is the intensity of the Ca^2+^-depleted sample.

### Fractional population of intermediate states

The fractional populations of ligated species shown in Figure 9, *A* and *B* were calculated with a standard Boltzmann distribution, where the probability (ƒ_s_) of a species (s) is given by Equation 6:

(6) $$f_{s}=\frac{\exp\left(-\Delta G_{s}/RT\right){\left[X\right]}^{j}}{\sum_{s,j}\;\exp-\Delta G_{s}/RT){\left[X\right]}^{j}}$$

where [X] is the free [Ca^2+^], j is the stoichiometry of Ca^2+^ bound by species s, and ΔG_s_ represents the free energy of species s, which includes the intrinsic binding affinity for each site (k_I_ and k_II_ for CaM_N_, and k_III_ and k_IV_ for CaM_C_) and cooperative interactions between the sites (k_I-II_ or k_III-IV_). The curves in Figure 9, *A* and *B* were simulated using the ΔG_1_ values for Ca^2+^ binding by CaM in the CaM+FGF12B+Na_V_1.2_CTD_ or CaM+FGF12A+Na_V_1.2_CTD_ complexes reported in Table 4 and assumed that the intrinsic Ca^2+^-binding affinities of both sites in each domain were equal (*i.e.*, k_I_ = k_II_ = k_N_ and k_III_ = k_IV_ = k_C_), as described previously (23). For CaM in the CaM+FGF12B+Na_V_1.2_CTD_ complex k_N_ and k_I-II_ were 1.39 × 10^4^ M^−1^ and 284.3, respectively, and k_C_ and k_III-IV_ were 5.36 × 10^3^ M^−1^ and 1.5, respectively. For CaM in the CaM+FGF12A+Na_V_1.2_CTD_ complex k_N_ and k_I-II_ were 1.04 × 10^5^ M^−1^ and 11.9, respectively, and k_C_ and k_III-IV_ were 4.30 × 10^3^ M^−1^ and 198.1, respectively.

### Modeling of FGF12A and FGF13A CaMBD bound to (Ca^2+^)_4_-CaM

Models of (Ca^2+^)_4_-CaM bound to the FGF12 (aa 38–64) and FGF13A (aa 35–60) CaMBD were generated by aligning idealized α-helical models of both FGF CaMBDs to the crystallographic structure of (Ca^2+^)_4_-CaM bound to the NMDA receptor CaMBD (2HQW.pdb) (68). The α-helical models FGF12A CaMBD and FGF13A CaMBD were aligned to the structure with NMDA receptor CaMBD residues 880–886 in PyMOL (Schrödinger LLC). Coordinates of the initial models were then energy-minimized with YASARA (105) using the default em_runclean macro.

## Data availability

Data reported in this publication are shown in the figures and contained within the article or available upon request from the corresponding author (Madeline A. Shea, madeline-shea@uiowa.edu).

## Supporting information

This article contains supporting information.

## Conflicts of interest

The authors declare that they have no conflicts of interest with the contents of this article.
