# Supplementary material for: Ca2+-saturated calmodulin binds tightly to the N-terminal domain of A-type fibroblast growth factor homologous factors
Source: J Biol Chem. 2021 Feb 24;296:100458. doi: 10.1016/j.jbc.2021.100458 (PMC8059062; doi:10.1016/j.jbc.2021.100458)
Supplement: Figures S1 to S9 and Tables S1 to S8 [file mmc1.pdf]

Supporting Information

for

Ca^{2+}-Saturated calmodulin binds tightly to the N-terminal domain of A-type fibroblast growth factor homologous factors

Ryan Mahling, Cade R. Rahlf, Samuel C. Hansen, Matthew R. Hayden, and Madeline A. Shea*

Department of Biochemistry, Carver College of Medicine, University of Iowa
Iowa City, IA, 52242-1109, USA

Author ORCID Identifiers

Hansen	0000-0003-4114-1355
Hayden	0000-0002-9680-6644
Mahling	0000-0002-4590-2724
Rahlf	0000-0001-9748-247X
Shea	0000-0003-0814-7069

*Corresponding author:
E-mail: madeline-shea@uiowa.edu
Telephone: (319) 335-7885
FAX: (319) 335-9570

These studies were supported by the Interdisciplinary Training in Pain Research Program NIH T32 NS045549 (RM); Iowa Center for Research by Undergraduates (ICRU); University of Iowa Carver College of Medicine FUTURE in Biomedicine Fellowship (MRH); the US National Institutes of Health R01 GM57001 (MAS). The content of this manuscript is solely the responsibility of the authors and does not necessarily represent the official views of the US National Institutes of Health.

Running Title: $(Ca^{2+})_4$-CaM Tightly Binds the NTD of A-Type FGFs

Keywords: Molecular recognition, Ca^{2+}-dependent interaction, voltage-gated sodium channel, protein-protein interaction, NMR, FRET, FHF, Biosensor, Thermodynamics, Allostery, Affinity

References

Supporting Information – Figure Legends

1. Crooks, G. E., Hon, G., Chandonia, J. M., and Brenner, S. E. (2004) WebLogo: a sequence logo generator. *Genome Res* **14**, 1188-1190
(Unique to Supporting Information, Figure S1)

2. Gautier, R., Douguet, D., Antonny, B., and Drin, G. (2008) HELIQUEST: a web server to screen sequences with specific alpha-helical properties. *Bioinformatics (Oxford, England)* **24**, 2101-2102
(Same as reference 97 in primary article)

3. Shen, Y., and Bax, A. (2010) SPARTA+: a modest improvement in empirical NMR chemical shift prediction by means of an artificial neural network. *J Biomol NMR* **48**, 13-22
(Same as reference 55 in primary article)

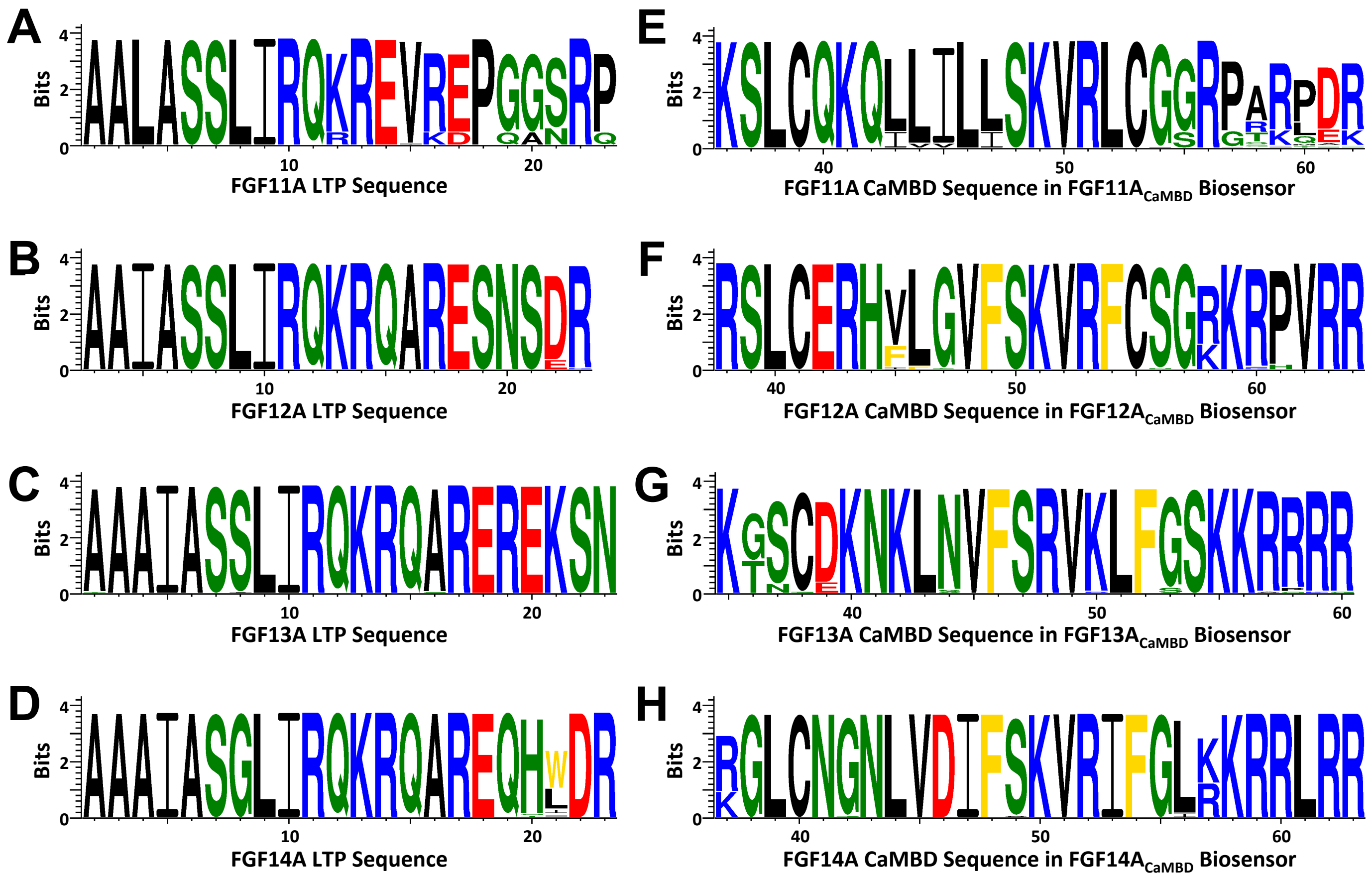

Figure S1 (*Related to Fig. 2)*
Comparison of A-Type FGF LTP and CaMBD Sequences Contained in Biosensors

A-D: Weblogo [1] comparison of the FGF11A (**A**, a.a. 2 – 23, 60 species), FGF12A (**B**, a.a. 3 – 23, 56 species), FGF13A (**C**, a.a. 2 – 23, 63 species), and FGF14A (**D**, a.a. 2 – 23, 48 species) LTP sequences across species.

E-H: Weblogo [1] comparison of the FGF11A (**E**, a.a. 36 – 62, 60 species), FGF12A (**F**, a.a. 38 – 64, 56 species), FGF13A (**G**, a.a. 35 – 60, 53 species) and FGF14A (**G**, a.a. 37 – 63, 48 species) sequences used in the CaMBD biosensors across species.

Figure S2

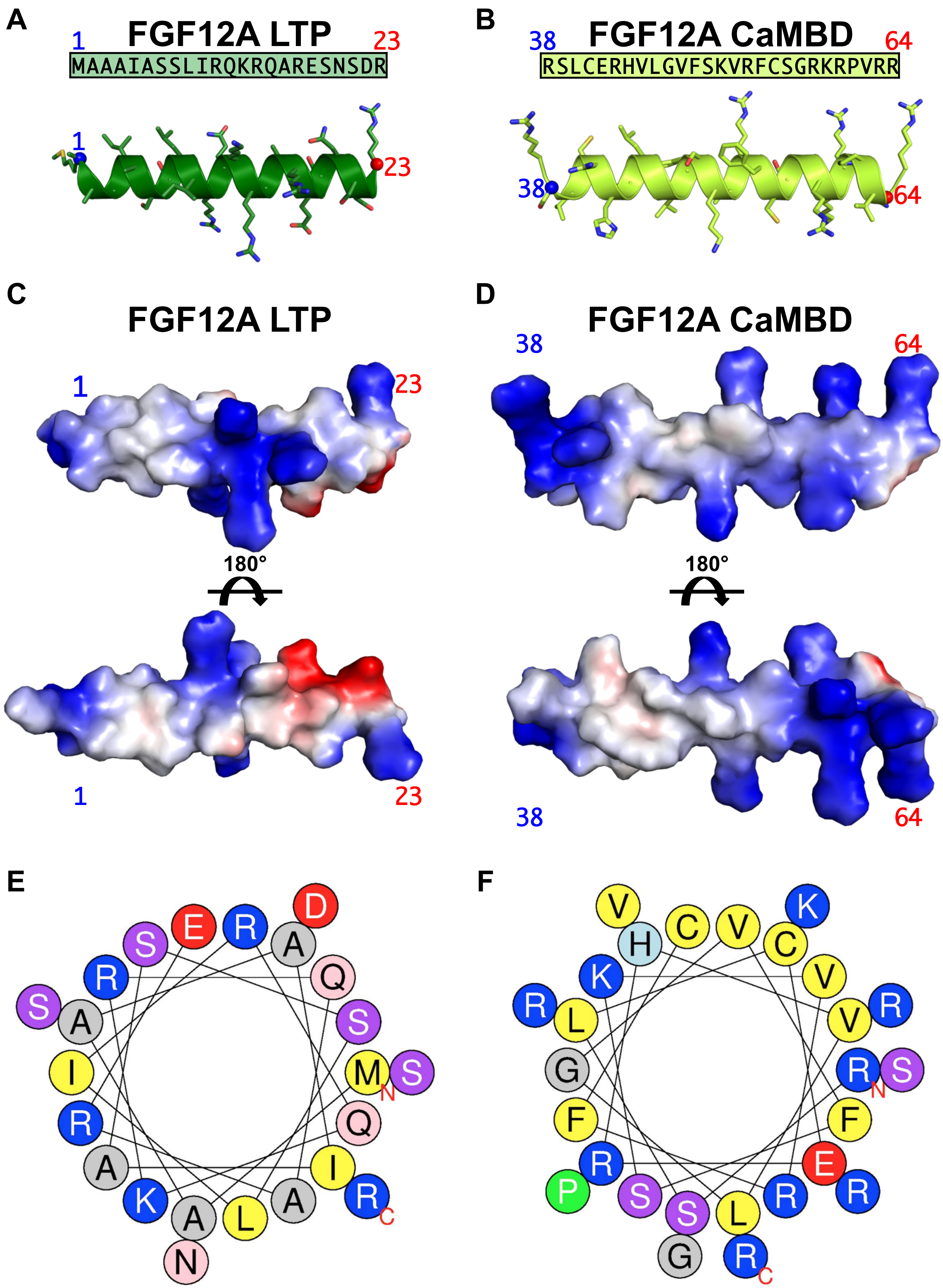
A
FGF12A LTP
1
23
MAAAIASSLIRQKRQARESNSDR
B
FGF12A CaMBD
38
64
RSLCERHVLGVFSKVRFCSGRKRPVRR
C
FGF12A LTP
D
FGF12A CaMBD
180°
180°
E
F

Figure S2 (*Related to Fig. 2)*

Models of FGF12A LTP and CaMBD

A: Top: Human FGF12A LTP (a.a. 1-23) sequence. Bottom: FGF12A LTP sequences depicted as sticks on a modeled α-helix. Cα of residues 1 (blue) and 23 (red) are spheres.

B: Top: Human FGF12A CaMBD (a.a. 38-64) sequence. Bottom: FGF12A CaMBD sequence depicted as sticks on a modeled α-helix. Cα of residues 38 (blue) and 64 (red) are spheres.

C-D: Calculated vacuum electrostatic surface (positive/blue, negative/red, neutral/white) of the FGF12A LTP (**C**) and FGF12A CaMBD (**D**).

E-F: Helical wheels of FGF12A LTP (**E**) and FGF12A CaMBD (**F**). Helical wheels were generated with HeliQuest (https://heliquest.ipmc.cnrs.fr/) [2].

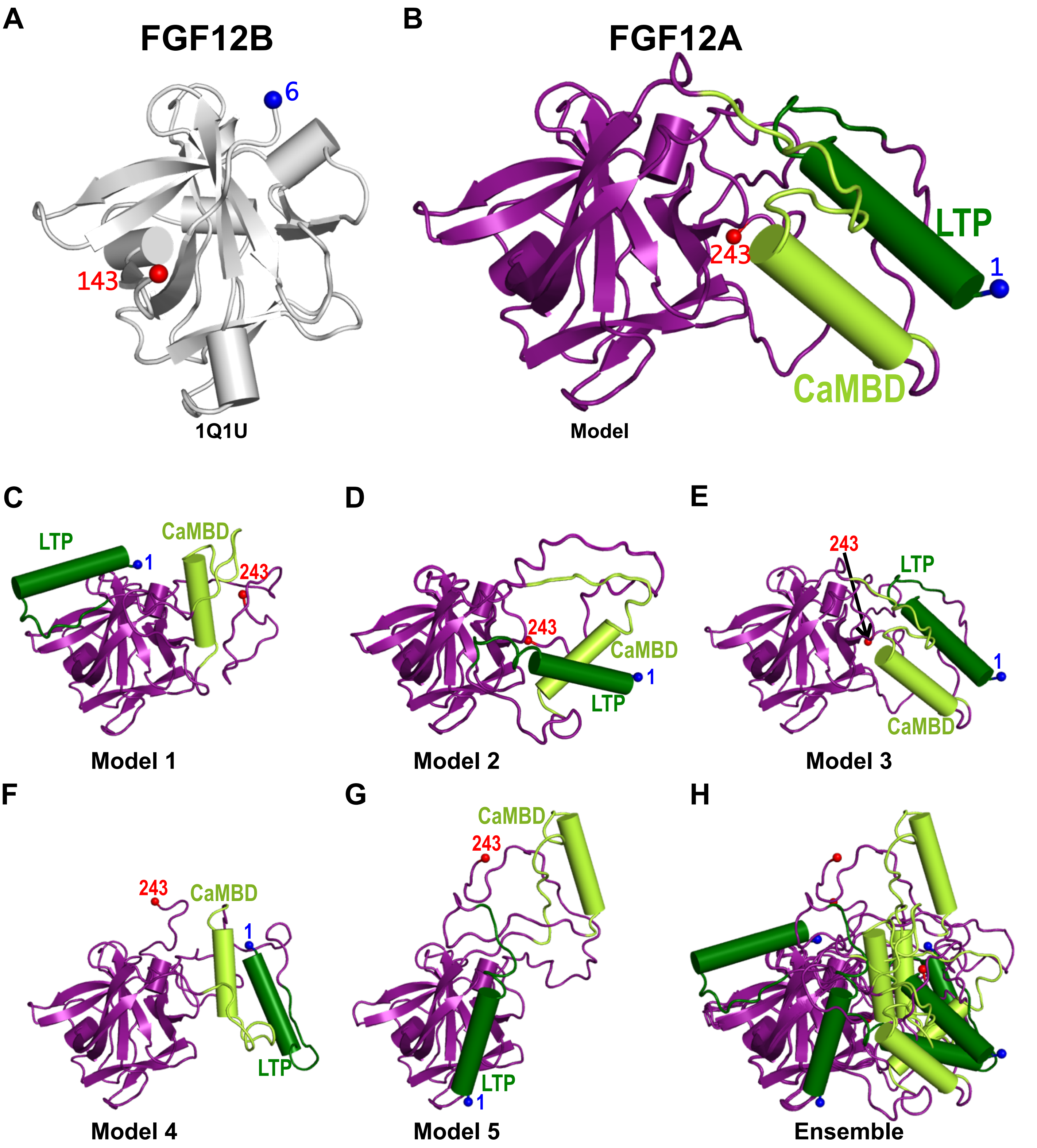

Figure S3 (*Related to Fig. 2*) **Ensemble of FGF12A Models**

A-B: Alignment of FGF12B (**B**, 1Q1U, gray) and model 3 of FGF12A (**C**, purple, LTP/green, CaMBD/limon). The FGF12A model was aligned with FGF12B a.a. 6-143. Cα of FGF12B residues 6 (blue) and 143 (red) and FGF12A residues 1 (blue) and 243 (red) are shown as spheres.

C-G: Orientations of the LTP (green) and CaMBD (limon) in the models of FGF12A (purple). Cα of FGF12A residues 1 (blue) and 243 (red) are shown as spheres.
H: Ensemble of FGF12A aligned with FGF12A (purple) a.a. 69-207. The FGF12A LTP is green and CaMBD is limon. Cα of FGF12A residues 1 (blue) and 243 (red) are shown as spheres.

Figure S4

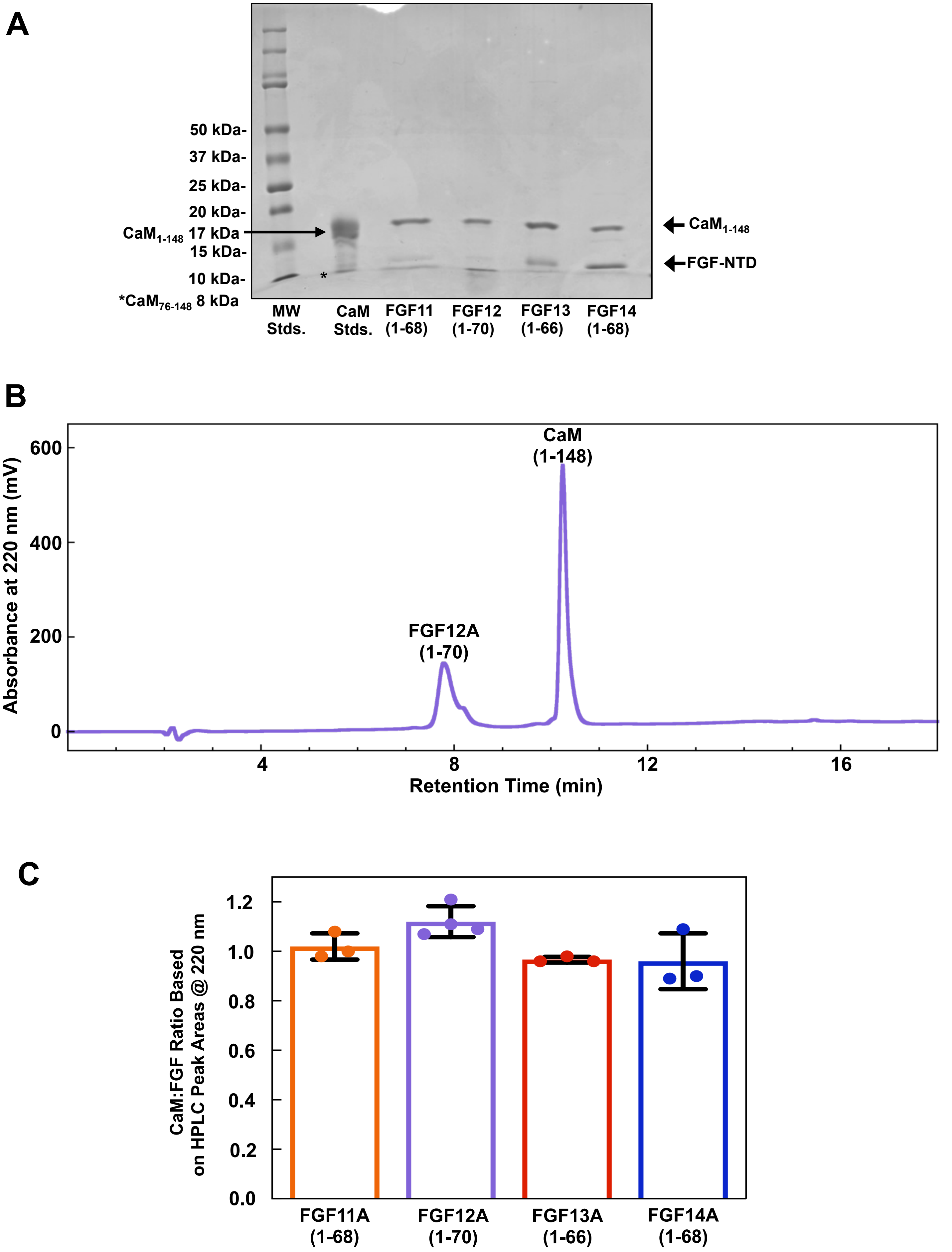
A
50 kDa-
37 kDa-
25 kDa-
20 kDa-
CaM1-148 17 kDa
15 kDa-
10 kDa-
*CaM76-148 8 kDa
*
CaM1-148
FGF-NTD
MW Stds.
CaM Stds.
FGF11 (1-68)
FGF12 (1-70)
FGF13 (1-66)
FGF14 (1-68)
B
Absorbance at 220 nm (mV)
600
400
200
0
4
8
12
16
Retention Time (min)
CaM (1-148)
FGF12A (1-70)
C
CaM:FGF Ratio Based on HPLC Peak Areas @ 220 nm
1.2
1.0
0.8
0.6
0.4
0.2
0.0
FGF11A (1-68)
FGF12A (1-70)
FGF13A (1-66)
FGF14A (1-68)

Figure S4 (*Related to Fig. 4*) **$(Ca^{2+})_4$-CaM Co-Purifies with A-Type FGF NTDs at a 1:1 Ratio**

A: 17% SDS-PAGE gel of the $(Ca^{2+})_4$-CaM+FGF11A_{NTD}, FGF12A_{NTD}, FGF13A_{NTD}, or FGF14A_{NTD} complexes following co-purification.

B: rpHPLC chromatogram of the co-purified $(Ca^{2+})_4$-CaM+FGF12A_{NTD} complex.

C: Molar ratio of $(Ca^{2+})_4$-CaM to FGF11A_{NTD} (orange), FGF12A_{NTD} (purple), FGF13A_{NTD} (red) or FGF14A_{NTD} (blue) in the copurified complexes. The molar ratio was determined by comparing the area of the peak at 220 nm for CaM to that of FGF NTD construct in rpHPLC chromatograms. Error bars show the standard deviation of ≥ 3 experiments.

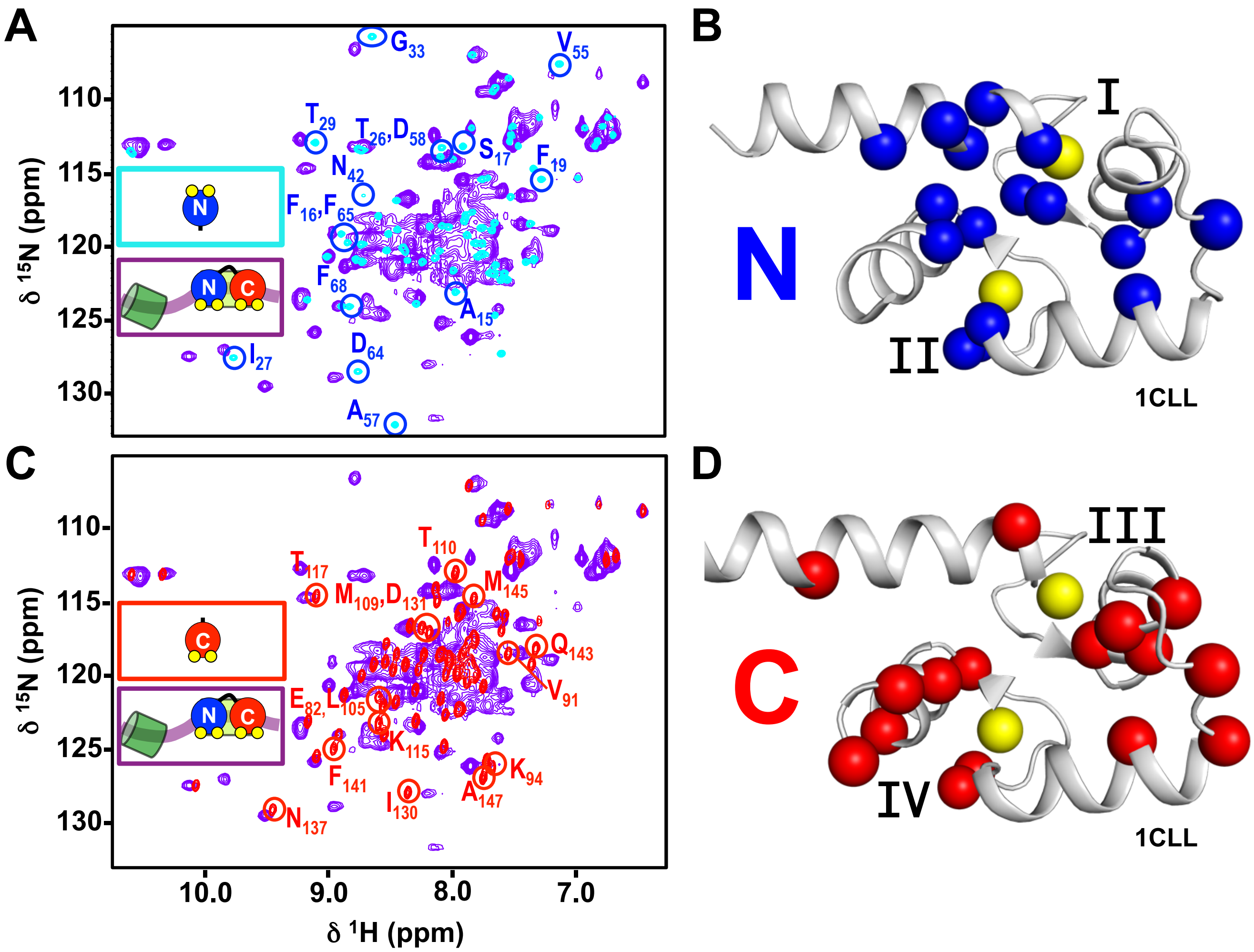

Figure S5 (*Related to Fig. 4*) **FGF12A$_{NTD}$ Binding Perturbs Residues in Both CaM Domains**

A: Overlay of the ^{15}N-HSQC spectra of ^{15}N-(Ca^{2+})$_2$-CaM$_N$ (blue) and ^{15}N-(Ca^{2+})$_4$-CaM+^{14}N-FGF12A$_{NTD}$ (purple). Peaks labeled in ^{15}N-(Ca^{2+})$_2$-CaM$_N$ spectrum are shifted in the ^{15}N-(Ca^{2+})$_4$-CaM+^{14}N-FGF12A$_{NTD}$ spectrum.

B: Cα of CaM$_N$ residues (blue spheres) labeled in panel **A** on a structure of (Ca^{2+})$_4$-CaM (1CLL, gray helices). For clarity of residues 1-75 of 1CLL are shown.

C: Overlay of the ^{15}N-HSQC spectra of ^{15}N-(Ca^{2+})$_2$-CaM$_C$ (red) and ^{15}N-(Ca^{2+})$_4$-CaM+^{14}N-FGF12A$_{NTD}$ (purple). Peaks labeled in ^{15}N-(Ca^{2+})$_2$-CaM$_C$ spectrum are shifted in the ^{15}N-(Ca^{2+})$_4$-CaM+^{14}N-FGF12A$_{NTD}$ spectrum.

D: Cα of CaM$_C$ residues (red spheres) labeled in panel **C** on a structure of (Ca^{2+})$_4$-CaM (1CLL, gray helices). For clarity of residues 76-148 of 1CLL are shown.

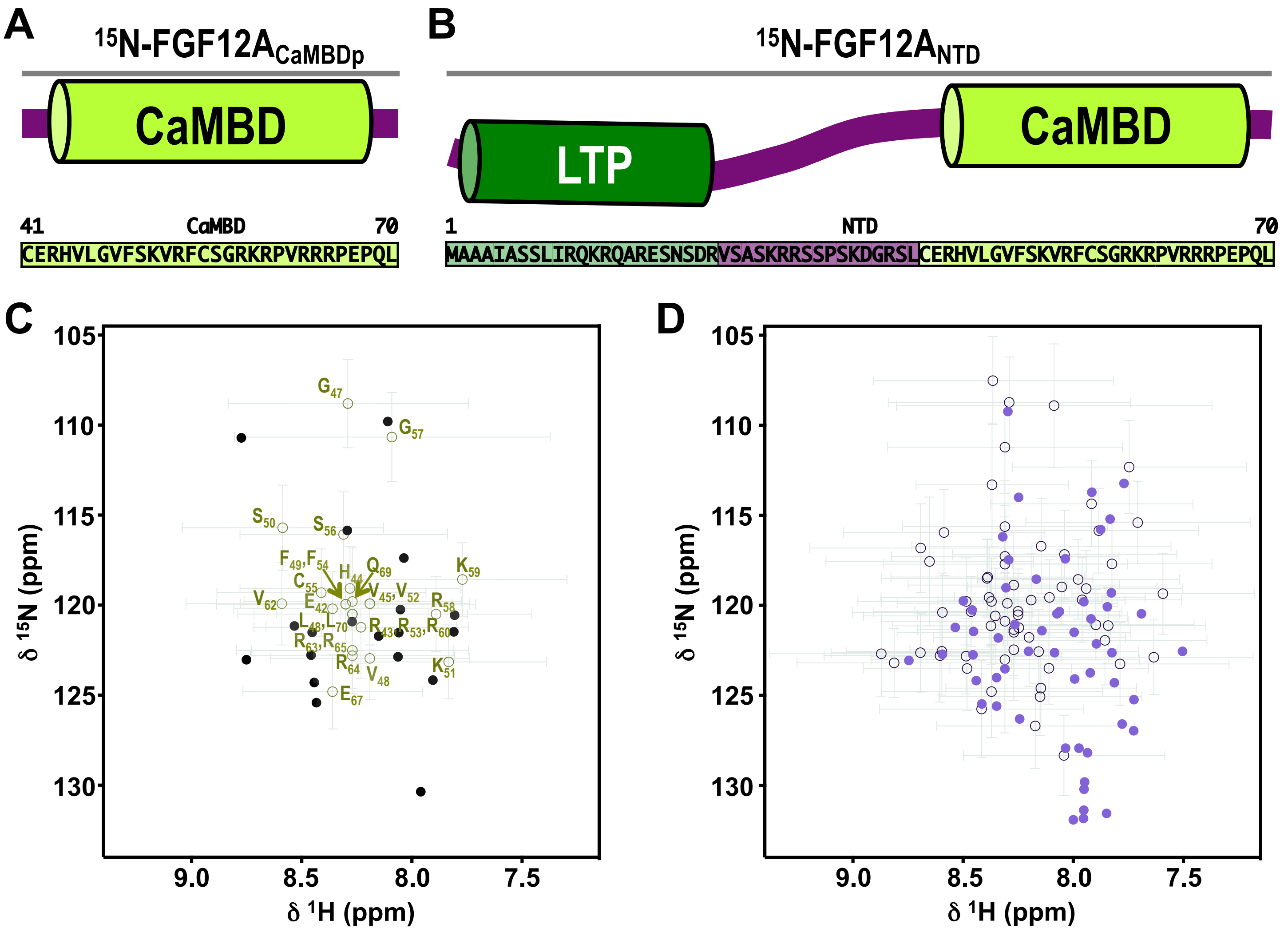

Figure S6 (*Related to Fig. 4)*
Predicted ^{15}N-HSQC Spectra of ^{15}N-FGF12A$_{CaMBDp}$ and ^{15}N-FGF12A LTP

A-B: Schematic depictions and sequences of the FGF12A$_{CaMBDp}$ (**A,** limon cylinder) and FGF12A$_{NTD}$ (**B**, LTP/green cylinder, CaMBD/limon green cylinder). The sequences have been shaded to match the schematics.

C: Peaks observed in the ^{15}N-HSQC spectrum of ^{15}N-FGF12A$_{CaMBDp}$+^{14}N-(Ca^{2+})$_4$-CaM (closed, black) are compared to peak positions predicted by SPARTA+ [3] for residues 41 – 70 (FGF12A$_{CaMBDp}$/open, olive) of the energetically most favorable Robetta model (model 1, Fig. 8A) of full-length FGF12A (olive).

D: Peaks observed in the ^{15}N-HSQC spectrum of ^{15}N-FGF12A$_{NTD}$+^{14}N-(Ca^{2+})$_4$-CaM (closed, purple) are compared to peak positions predicted by SPARTA+ [55] for residues 1 – 70 (FGF12A$_{NTD}$/open, dark purple) of the energetically most favorable Robetta model (model 1, Fig. 8A) of full-length FGF12A.

Figure S7

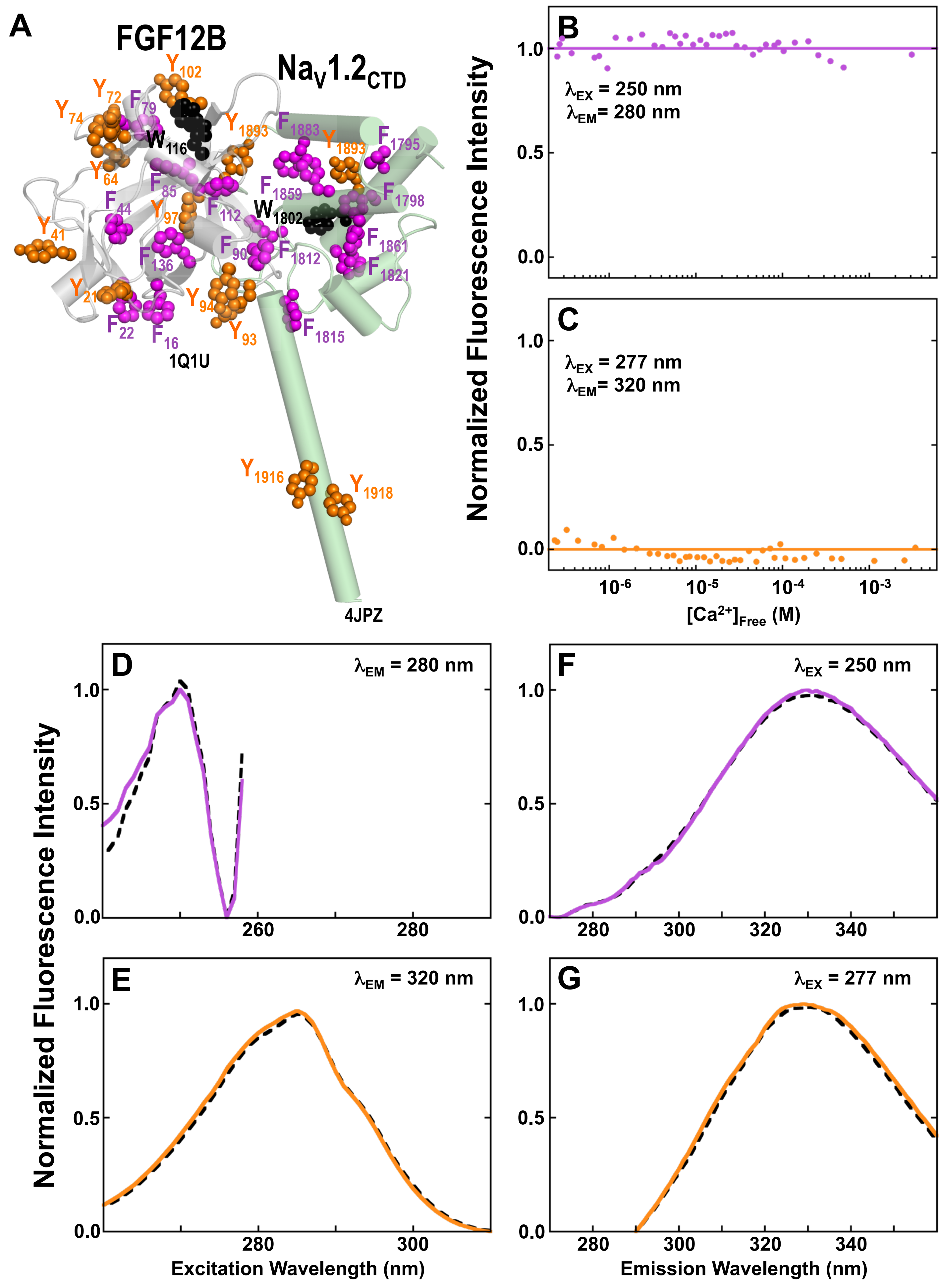
A
FGF12B
NaV1.2CTD
Y102
Y72
Y74
F79
W116
Y1893
F1883
Y1893
F1795
Y64
F85
F1859
F1798
F44
Y97
F112
W1802
Y41
F90
F1812
F1861
F136
F1821
Y21
Y94
F22
F16
Y93
F1815
1Q1U
Y1916
Y1918
4JPZ
B
λEX = 250 nm
λEM= 280 nm
C
λEX = 277 nm
λEM= 320 nm
Normalized Fluorescence Intensity
[Ca2+]Free (M)
D
λEM = 280 nm
E
λEM = 320 nm
F
λEX = 250 nm
G
λEX = 277 nm
Normalized Fluorescence Intensity
Excitation Wavelength (nm)
Emission Wavelength (nm)

Figure S7 (*Related to Fig. 6*)
Equilibrium Ca^{2+} Titrations of the FGF12B+$Na_V1.2_{CTD}$ Complex

A: Position of Phe (magenta, ball-and-stick), Tyr (orange, ball-and-stick) and Trp (black, ball-and-stick) residues in a model of FGF12B (1Q1U, gray) bound to $Na_V1.2_{CTD}$ (4JPZ, forest green). The model is a composite of FGF12B from 1Q1U aligned with FGF13U from 4JPZ via FGF13U residues 11-158. FGF13U and CaM in 4JPZ are not shown.

B-C: Equilibrium Ca^{2+} titrations of the FGF12B+$Na_V1.2_{CTD}$ complex. The average Phe (**B**) or Tyr (**C**) fluorescence intensity of FGF12B+$Na_V1.2_{CTD}$ in each titration was normalized to the average net change in intensity in Ca^{2+} titrations of CaM+FGF12B+$Na_V1.2_{CTD}$. The solid lines at 1 (**B**) and 0 (**C**) are for reference.

D: Steady-state excitation spectra (λ_{EM} = 280 nm) of the FGF12B+$Na_V1.2_{CTD}$ complex in Ca^{2+}-depleted conditions (black, dashed) and in the presence or 10 mM Ca^{2+} (pink, solid).

E: Steady-state excitation spectra (λ_{EM} = 320 nm) of the FGF12B+$Na_V1.2_{CTD}$ complex in Ca^{2+}-depleted conditions (black, dashed) and in the presence of 10 mM Ca^{2+} (orange, solid).

F: Steady-state emission spectra (λ_{EX} = 250 nm) of the FGF12B+$Na_V1.2_{CTD}$ complex in Ca^{2+}-depleted conditions (black, dashed) and in the presence of 10 mM Ca^{2+} (pink, solid).

G: Steady-state emission spectra (λ_{EX} = 277 nm) of the FGF12B+$Na_V1.2_{CTD}$ complex in Ca^{2+}-depleted conditions (black, dashed) and in the presence of 10 mM Ca^{2+} (orange, solid).

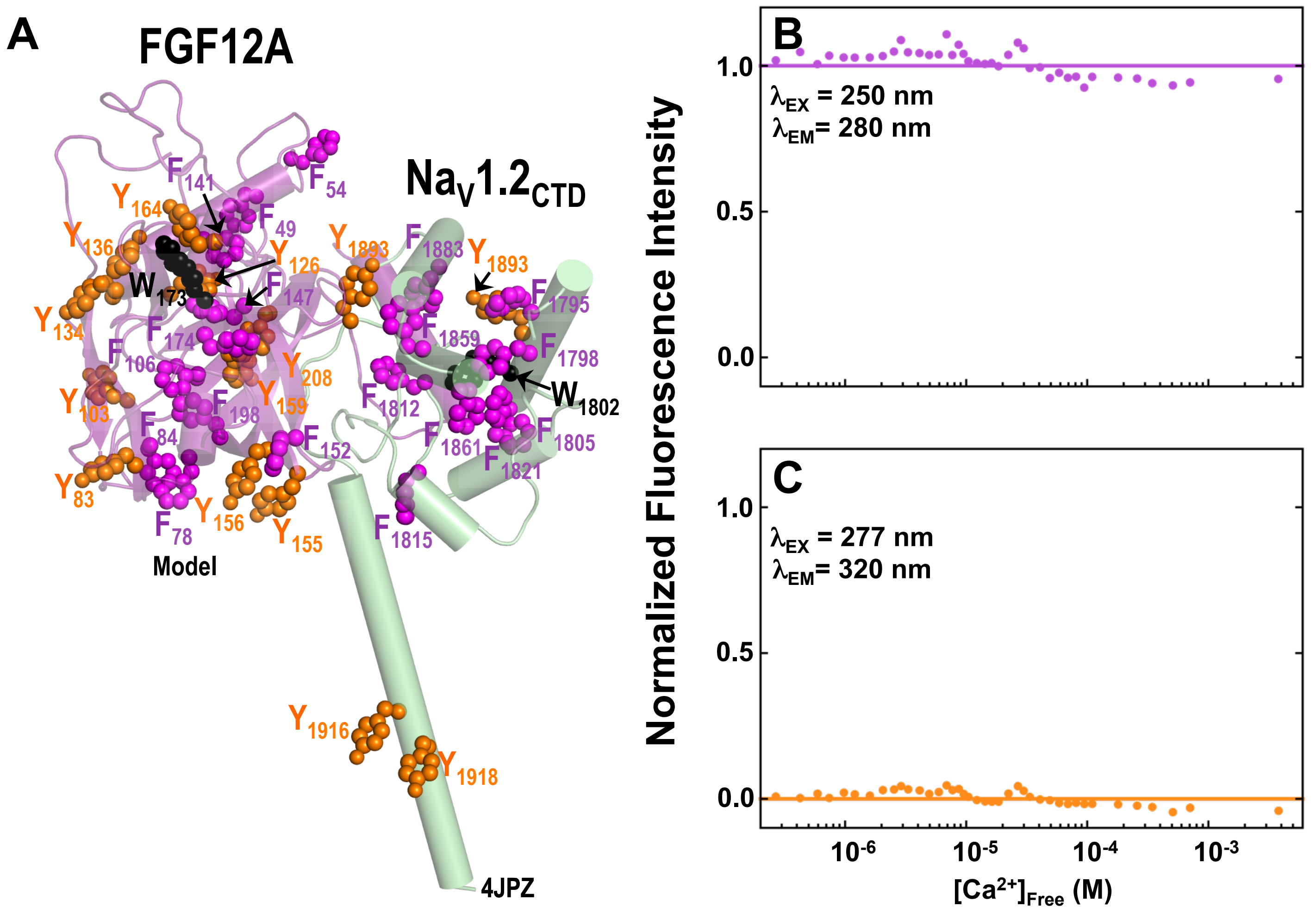

Figure S8 (*Related to Fig. 6*)
Equilibrium Ca^{2+} Titrations of the FGF12A+$Na_V1.2_{CTD}$ Complex

A: Position of Phe (magenta, ball-and-stick), Tyr (orange, ball-and-sticks), and Trp (black, ball-and-stick) residues in a model of FGF12A (model, purple) bound to $Na_V1.2_{CTD}$ (4JPZ, forest green). The model is a composite of the FGF12A aligned with FGF13U from 4JPZ via FGF13U residues 11-158. FGF13U and CaM in 4JPZ are not shown.

B-C: Equilibrium Ca^{2+} titrations of the FGF12A+$Na_V1.2_{CTD}$ complex. The average Phe (**B**) or Tyr (**C**) fluorescence intensity of FGF12A+$Na_V1.2_{CTD}$ in each titration was normalized to the average net change in intensity in Ca^{2+} titrations of CaM+FGF12A+$Na_V1.2_{CTD}$. The solid lines at 1 (**B**) and 0 (**C**) are for reference.

Figure S9

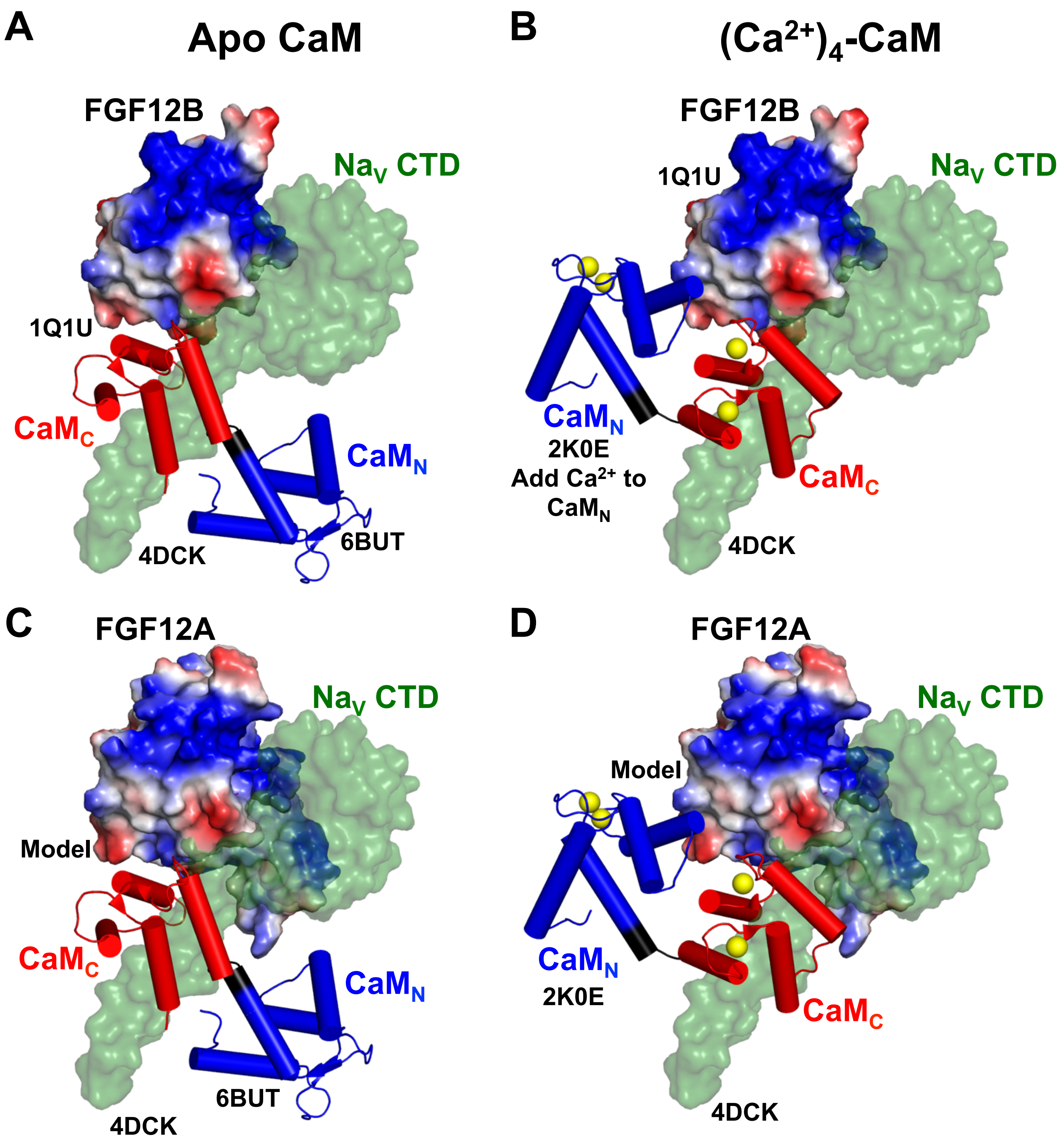
A
Apo CaM
B
(Ca2+)4-CaM
FGF12B
NaV CTD
1Q1U
CaMC
CaMN
4DCK
6BUT
FGF12B
1Q1U
NaV CTD
CaMN
2K0E
Add Ca2+ to
CaMN
CaMC
4DCK
C
FGF12A
NaV CTD
Model
CaMC
CaMN
6BUT
4DCK
D
FGF12A
NaV CTD
Model
CaMN
2K0E
CaMC
4DCK

Figure S9 (*Related to Fig. 9*)
Models of CaM and FGF12B or FGF12A Bound to an Na_V CTD

In all models the Na_V CTD (4DCK) is shown as a transparent surface (green), FGF12B (1Q1U) and FGF12A are shown as a vacuum electrostatic surface (basic/blue, acidic/red, neutral/white), CaM residues 1-75 are blue, 76-80 are black, and 81-148 are red. Ca^{2+} are shown as yellow spheres where appropriate.

A: Model of apo CaM and FGF12B bound to an Na_V CTD. Model was generated by aligning the solution structure of apo CaM+$Na_V1.2_{IQp}$ (6BUT) to the crystallographic structure of apo CaM+FGF13B+Na_V1.5 CTD (4DCK) with CaM residues 101-112 and 117-128. The crystallographic structure of FGF12B (1Q1U) was dock using FGF13U residues 11–136.

B: Model of $(Ca^{2+})_4$-CaM and FGF12B bound to an Na_V CTD. Model was generated by docking the structure of $(Ca^{2+})_2$-CaM_C+Na_V1.2 IQ motif (2M5E) with the Na_V1.5 IQ motif in the crystallographic structure of apo CaM+FGF13U+Na_V1.5 CTD (4DCK) via Na_V1.2 IQ motif residues 1904-1924. A solution structure of $(Ca^{2+})_4$-CaM (2K0E) was docked via CaM residues 101-112 and 117-128 of 2M5E. FGF12B (1Q1U) was docked through residues 11–136 of FGF13U.

C: Model of apo CaM and FGF12A bound to an Na_V CTD. Model was generated by aligning the solution structure of apo CaM+$Na_V1.2_{IQp}$ (6BUT) to the crystallographic structure of apo CaM+FGF13U+Na_V1.5 CTD (4DCK) with CaM residues 101-112 and 117-128. The modeled structure of FGF12A was dock using FGF13U residues 11–136.

D: Model of $(Ca^{2+})_4$-CaM and FGF12A bound to an Na_V CTD. Model was generated by docking the structure of $(Ca^{2+})_2$-CaM_C+Na_V1.2 IQ motif (2M5E) with the Na_V1.5 IQ motif in the crystallographic structure of apo CaM+FGF13U+Na_V1.5 CTD (4DCK) via Na_V1.2 IQ motif residues 1904-1924. A solution structure of $(Ca^{2+})_4$-CaM (2K0E) was docked via CaM residues 101-112 and 117-128 of 2M5E. The model structure of FGF12A was docked through residues 11–136 of FGF13U.
For clarity, ribbon drawings of CaM and FGF13U of 4DCK, CaM and Na_V1.2 IQ motif of 2M5E and Na_V1.2 IQ motif of 6BUT are not shown.

Table S1

(Related to Fig. 2)

Comparison of FGF11A LTP Sequences From 60 Species

LTP Sequence Table (Completely Conserved Positions Highlighted in Yellow, Positions Conserved in ≥ 90% of Species Highlighted in Green)

	Species	Common Name	Start Residue	1	2	3	4	5	6	7	8	9	10	11	12	13	14	15	16	17	18	19	20	21	22	23	End Residue
				M	A	A	L	A	S	S	L	I	R	Q		R	E								R		
1	Homo sapiens	Human	1	M	A	A	L	A	S	S	L	I	R	Q	K	R	E	V	R	E	P	G	G	S	R	P	23
2	Danio rerio	Zebrafish	1	M	A	A	L	A	S	S	L	I	R	Q	R	R	E	V	K	D	P	K	A	N	R	Q	23
3	Pangasianodon hypophthalmus	Iridescent shark	1	M	A	A	L	A	S	S	L	I	R	Q	R	R	E	V	K	D	Q	Q	A	N	R	Q	23
4	Anarrhichthys ocellatus	Wolf eel	1	M	A	A	L	A	S	S	L	I	R	Q	R	R	E	V	K	D	P	Q	A	N	R	Q	23
5	Myripristis murdjan	Soldierfish	1	M	A	A	L	A	S	S	L	I	R	Q	R	R	E	V	K	D	P	Q	A	N	R	Q	23
6	Hippoglossus hippoglossus	Righteye Founder	1	M	A	A	L	A	S	S	L	I	R	Q	R	R	E	V	K	D	P	Q	A	N	R	Q	23
7	Oreochromis aureus	Blue Tilpia	1	M	A	A	L	A	S	S	L	I	R	Q	R	R	E	V	K	D	P	Q	A	N	R	Q	23
8	Denticeps clupeoides	Denticle Herring	1	M	A	A	L	A	S	S	L	I	R	Q	R	R	E	V	K	D	P	Q	A	N	R	Q	23
9	Astyanax mexicanus	Mexican Tetra	1	M	A	A	L	A	S	S	L	I	R	Q	R	R	E	V	K	D	P	Q	A	N	R	Q	23
10	Cyprinus carpio	Carp	1	M	A	A	L	A	S	S	L	I	R	Q	R	R	E	V	K	D	P	Q	A	N	R	Q	23
11	Clupea harengus	Atlantic Herring	1	M	A	A	L	A	S	S	L	I	R	Q	R	R	E	I	K	D	P	Q	A	N	R	Q	23
12	Scophthalmus maximus	Turbot	1	M	A	A	L	A	S	S	L	I	R	Q	R	R	E	V	K	D	P	Q	A	N	R	Q	23
13	Microcebus murinus	Gray Mouse Lemur	1	M	A	A	L	A	S	S	L	I	R	Q	K	R	E	V	R	E	P	G	G	S	R	P	23
14	Vicugna pacos	Alpaca	1	M	A	A	L	A	S	S	L	I	R	Q	K	R	E	V	R	E	P	G	G	S	R	P	23
15	Macaca mulatta	Rhesus Maceque	1	M	A	A	L	A	S	S	L	I	R	Q	K	R	E	V	R	E	P	G	G	S	R	P	23
16	Papio anubis	Olive Baboon	1	M	A	A	L	A	S	S	L	I	R	Q	K	R	E	V	R	E	P	G	G	S	R	P	23
17	Trachypithecus francoisi	Tonkin leaf monkey	1	M	A	A	L	A	S	S	L	I	R	Q	K	R	E	V	R	E	P	G	G	S	R	P	23
18	Castor canadensis	North American Beaver	1	M	A	A	L	A	S	S	L	I	R	Q	K	R	E	V	R	E	P	G	G	S	R	P	23
19	Rhinopithecus roxellana	Golden snub-nosed monkey	1	M	A	A	L	A	S	S	L	I	R	Q	K	R	E	V	R	E	P	G	G	S	R	P	23
20	Tupaia chinensis	Treeshrew	1	M	A	A	L	A	S	S	L	I	R	Q	K	R	E	V	R	E	P	G	G	S	R	P	23
21	Carlito syrichta	Philippine Tarsier	1	M	A	A	L	A	S	S	L	I	R	Q	K	R	E	V	R	E	P	G	G	S	R	P	23
22	Canis lupus familiaris	Dog	1	M	A	A	L	A	S	S	L	I	R	Q	K	R	E	V	R	E	P	G	G	S	R	P	23
23	Crocuta crocuta	Spotted Hyena	1	M	A	A	L	A	S	S	L	I	R	Q	K	R	E	V	R	E	P	G	G	S	R	P	23
24	Canis lupus dingo	Dingo	1	M	A	A	L	A	S	S	L	I	R	Q	K	R	E	V	R	E	P	G	G	S	R	P	23
25	Sapajus apella	Tuffed Capuchin	1	M	A	A	L	A	S	S	L	I	R	Q	K	R	E	V	R	E	P	G	G	N	R	P	23
26	Aotus nancymaae	Night Monkey	1	M	A	A	L	A	S	S	L	I	R	Q	K	R	E	V	R	E	P	G	G	N	R	P	23
27	Felis catus	Cat	1	M	A	A	L	A	S	S	L	I	R	Q	K	R	E	V	R	E	P	G	G	S	R	P	23
28	Zalophus californianus	California Sea Lion	1	M	A	A	L	A	S	S	L	I	R	Q	K	R	E	V	R	E	P	G	G	S	R	P	23
29	Nomascus leucogenys	Northern White-Cheeked Gibbon	1	M	A	A	L	A	S	S	L	I	R	Q	K	R	E	V	R	E	P	G	G	S	R	P	23
30	Callorhinus ursinus	Northern Fur Seal	1	M	A	A	L	A	S	S	L	I	R	Q	K	R	E	V	R	E	P	G	G	S	R	P	23
31	Otolemur garnettii	Northern Greater Galago	1	M	A	A	L	A	S	S	L	I	R	Q	K	R	E	V	R	E	P	G	G	S	R	P	23
32	Pteropus alecto	Black Flying Fox	1	M	A	A	L	A	S	S	L	I	R	Q	K	R	E	V	R	E	P	G	G	S	R	P	23
33	Suricata suricatta	Meerkat	1	M	A	A	L	A	S	S	L	I	R	Q	K	R	E	V	R	E	P	G	G	S	R	P	23
34	Dasypus novemcinctus	None-banded Armadillo	1	M	A	A	L	A	S	S	L	I	R	Q	K	R	E	V	R	E	P	G	G	S	R	P	23
35	Bos taurus	Cattle	1	M	A	A	L	A	S	S	L	I	R	Q	K	R	E	V	R	E	P	G	G	S	R	P	23
36	Phyllostomus discolor	Pale Spear-nosed Bat	1	M	A	A	L	A	S	S	L	I	R	Q	K	R	E	V	R	E	P	G	G	S	R	P	23
37	Hylobates moloch	Silvery Gibbon	1	M	A	A	L	A	S	S	L	I	R	Q	K	R	E	V	R	E	P	G	G	S	R	P	23
38	Pongo abelii	Sumatran Orangutan	1	M	A	A	L	A	S	S	L	I	R	Q	K	R	E	V	R	E	P	G	G	S	R	P	23
39	Heterocephalus glaber	Naked mole-rat	1	M	A	A	L	A	S	S	L	I	R	Q	K	R	E	V	R	E	P	G	G	S	R	P	23
40	Equus caballus	Horse	1	M	A	A	L	A	S	S	L	I	R	Q	K	R	E	V	R	E	P	G	G	S	R	P	23
41	Ovis aries	Sheep	1	M	A	A	L	A	S	S	L	I	R	Q	K	R	E	V	R	E	P	G	G	S	R	P	23
42	Eptesicus fuscus	Big Brown Bat	1	M	A	A	L	A	S	S	L	I	R	Q	K	R	E	V	R	E	P	G	G	S	R	P	23
43	Leptonychotes weddellii	Weddell Seal	1	M	A	A	L	A	S	S	L	I	R	Q	K	R	E	V	R	E	P	G	G	S	R	P	23
44	Octodon degus	Common Degu	1	M	A	A	L	A	S	S	L	I	R	Q	K	R	E	V	R	E	P	G	G	S	R	P	23
45	Gorilla gorilla gorilla	Gorilla	1	M	A	A	L	A	S	S	L	I	R	Q	K	R	E	V	R	E	P	G	G	S	R	P	23
46	Cavia porcellus	Guinea Pig	1	M	A	A	L	A	S	S	L	I	R	Q	K	R	E	V	R	E	P	G	G	S	R	P	23
47	Callithrix jacchus	Common Marmoset	1	M	A	A	L	A	S	S	L	I	R	Q	K	R	E	V	R	E	P	G	G	N	R	P	23
48	Mus pahari	Gairdner's Shrewmouse	1	M	A	A	L	A	S	S	L	I	R	Q	K	R	E	V	R	E	P	G	G	S	R	P	23
49	Mirounga leonina	Southern Elepant Seal	1	M	A	A	L	A	S	S	L	I	R	Q	K	R	E	V	R	E	P	G	G	S	R	P	23
50	Mus musculus	House Mouse	1	M	A	A	L	A	S	S	L	I	R	Q	K	R	E	V	R	E	P	G	G	S	R	P	23
51	Ailuropoda melanoleuca	Giant Panda	1	M	A	A	L	A	S	S	L	I	R	Q	K	R	E	V	R	E	P	G	G	S	R	P	23
52	Grammomys surdaster	African Tree Rat	1	M	A	A	L	A	S	S	L	I	R	Q	K	R	E	V	R	E	P	G	G	S	R	P	23
53	Ursus arctos horribilis	Grizzly Bear	1	M	A	A	L	A	S	S	L	I	R	Q	K	R	E	V	R	E	P	G	G	S	R	P	23
54	Nannospalax galili	Blind Mole-rat	1	M	A	A	L	A	S	S	L	I	R	Q	K	R	E	V	R	E	P	G	G	S	R	P	23
55	Rattus norvegicus	Brown Rat	1	M	A	A	L	A	S	S	L	I	R	Q	K	R	E	V	R	E	P	G	G	S	R	P	23
56	Desmodus rotundus	Vampire Bat	1	M	A	A	L	A	S	S	L	I	R	Q	K	R	E	V	R	E	P	G	G	S	R	P	23
57	Meriones unguiculatus	Mongolian Gerbil	1	M	A	A	L	A	S	S	L	I	R	Q	K	R	E	V	R	E	P	G	G	S	R	P	23
58	Mus caroli	Ryukyu Mouse	1	M	A	A	L	A	S	S	L	I	R	Q	K	R	E	V	R	E	P	G	G	S	R	P	23
59	Urocitellus parryii	Arctic Ground Squirrel	1	M	A	A	L	A	S	S	L	I	R	Q	K	R	E	V	R	E	P	G	G	S	R	P	23
60	Lontra canadensis	North American River Otter	1	M	A	A	L	A	S	S	L	I	R	Q	K	R	E	V	R	E	P	G	G	S	R	P	23

Table S2

(*Related to Fig. 2)*

Comparison of FGF12A LTP Sequences From 56 Species

LTP Sequence Table (Completely Conserved Positions Highlighted in Yellow, Positions Conserved in ≥ 90% of Species Highlighted in Green)

	Species	Common Name	Start Residue	1	2	3	4	5	6	7	8	9	10	11	12	13	14	15	16	17	18	19	20	21	22	23	End Residue
				M		A	A	I	A	S	S	L	I	R	Q	K	R	Q	A	R	E	S	N	S			
1	Homo sapiens	Human	1	M	A	A	A	I	A	S	S	L	I	R	Q	K	R	Q	A	R	E	S	N	S	D	R	23
2	Danio rerio	Zebrafish	1	M	-	A	A	I	A	S	S	L	I	R	Q	K	R	Q	A	R	E	S	N	S	D	R	22
3	Apteryx rowi	Kiwi	1	M	A	A	A	I	A	S	S	L	I	R	Q	K	R	Q	A	R	E	S	N	S	D	R	23
4	Castor canadensis	North American Beaver	1	M	A	A	A	I	A	S	S	L	I	R	Q	K	R	Q	A	R	E	S	N	S	D	R	23
5	Cavia porcellus	Guinea Pig	1	M	A	A	A	I	A	S	S	L	I	R	Q	K	R	Q	A	R	E	S	N	S	D	R	23
6	Cricetulus griseus	Chinese Hamster	1	M	A	A	A	I	A	S	S	L	I	R	Q	K	R	Q	A	R	E	S	N	S	D	R	23
7	Cyprinus carpio	Common Carp	1	M	-	A	A	I	A	S	S	L	I	R	Q	K	R	Q	A	R	E	S	N	S	D	R	22
8	Fukomys damarensis	Damaraland Mole-Rat	1	M	A	A	A	I	A	S	S	L	I	R	Q	K	R	Q	A	R	E	S	N	S	D	R	23
9	Gallus gallus	Red Junglefowl	1	M	A	A	A	I	A	S	S	L	I	R	Q	K	R	Q	A	R	E	S	N	S	D	R	23
10	Lynx pardinus	Iberian Lynx	1	M	A	A	A	I	A	S	S	L	I	R	Q	K	R	Q	A	R	E	S	N	S	D	R	23
11	Macaca mulatta	Rhesus Macaque	1	M	A	A	A	I	A	S	S	L	I	R	Q	K	R	Q	A	R	E	S	N	S	D	R	23
12	Mirounga leonina	Southern Elephant Seal	1	M	A	A	A	I	A	S	S	L	I	R	Q	K	R	Q	A	R	E	S	N	S	D	R	23
13	Myotis davidii	Vesper Bat	1	M	A	A	A	I	A	S	S	L	I	R	Q	K	R	Q	A	R	E	S	N	S	D	R	23
14	Myotis lucifugus	Little Brown Bat	1	M	A	A	A	I	A	S	S	L	I	R	Q	K	R	Q	A	R	E	S	N	S	D	R	23
15	Rattus norvegicus	Brown Rat	1	M	A	A	A	I	A	S	S	L	I	R	Q	K	R	Q	A	R	E	S	N	S	D	R	23
16	Spheniscus humboldti	Humboldt Penguin	1	M	A	A	A	I	A	S	S	L	I	R	Q	K	R	Q	A	R	E	S	N	S	D	R	23
17	Thamnophis elegans	Western Terrestrial Garter Snake	1	M	A	A	A	I	A	S	S	L	I	R	Q	K	R	Q	A	R	E	S	N	S	E	R	23
18	Todus mexicanus	Puetro Rican Tody	1	M	A	A	A	I	A	S	S	L	I	R	Q	K	R	Q	A	R	E	S	N	S	D	R	23
19	Mus musculus	House Mouse	1	M	A	A	A	I	A	S	S	L	I	R	Q	K	R	Q	A	R	E	S	N	S	D	R	23
20	Arvicanthis niloticus	African grass rat	1	M	A	A	A	I	A	S	S	L	I	R	Q	K	R	Q	A	R	E	S	N	S	D	R	23
21	Phocoena sinus	Porpoise	1	M	A	A	A	I	A	S	S	L	I	R	Q	K	R	Q	A	R	E	S	N	S	D	R	23
22	Camelus dromedarius	Arabian Camel	1	M	A	A	A	I	A	S	S	L	I	R	Q	K	R	Q	A	R	E	S	N	S	D	R	23
23	Nannospalax galili	Mole-rat	1	M	A	A	A	I	A	S	S	L	I	R	Q	K	R	Q	A	R	E	S	N	S	D	R	23
24	Camelus ferus	Bactrian Camel	1	M	A	A	A	I	A	S	S	L	I	R	Q	K	R	Q	A	R	E	S	N	S	D	R	23
25	Rhinolophus ferrumequinum	Horseshoe Bat	1	M	A	A	A	I	A	S	S	L	I	R	Q	K	R	Q	A	R	E	S	N	S	D	R	23
26	Trichechus manatus latirostris	Florida Manatee	1	M	A	A	A	I	A	S	S	L	I	R	Q	K	R	Q	A	R	E	S	N	S	D	R	23
27	Lonchura striata domestica	Society Finch	1	M	A	A	A	I	A	S	S	L	I	R	Q	K	R	Q	A	R	E	S	N	S	D	R	23
28	Tupaia chinensis	Tree Shrew	1	M	A	A	A	I	A	S	S	L	I	R	Q	K	R	Q	A	R	E	S	N	S	D	R	23
29	Dasypus novemcinctus	Nine-Banded Armadillo	1	M	A	A	A	I	A	S	S	L	I	R	Q	K	R	Q	A	R	E	S	N	S	D	R	23
30	Ornithorhynchus anatinus	Platypus	1	M	A	A	A	I	A	S	S	L	I	R	Q	K	R	Q	A	R	E	S	N	S	D	R	23
31	Camarhynchus parvulus	Small Tree Finch	1	M	A	A	A	I	A	S	S	L	I	R	Q	K	R	Q	A	R	E	S	N	S	D	R	23
32	Dromaius novaehollandiae	Emu	1	M	A	A	A	I	A	S	S	L	I	R	Q	K	R	Q	A	R	E	S	N	S	D	R	23
33	Microcaecilia unicolor	Microcaecilia unicolor	1	M	A	A	A	I	A	S	S	L	I	R	Q	K	R	Q	A	R	E	S	N	S	D	R	23
34	Podarcis muralis	Wall Lizard	1	M	A	A	A	I	A	S	S	L	I	R	Q	K	R	Q	A	R	E	S	N	S	D	R	23
35	Geotrypetes seraphini	Gaboon Caecilian	1	M	A	A	A	I	A	S	S	L	I	R	Q	K	R	Q	A	R	E	S	N	S	D	R	23
36	Chrysemys picta bellii	Western Painted Turtle	1	M	A	A	A	I	A	S	S	L	I	R	Q	K	R	Q	A	R	E	S	N	S	E	R	23
37	Terrapene carolina triunguis	Three-Toed Box Turtle	1	M	A	A	A	I	A	S	S	L	I	R	Q	K	R	Q	A	R	E	S	N	S	E	R	23
38	Notechis scutatus	Tiger Snake	1	M	A	A	A	I	A	S	S	L	I	R	Q	K	R	Q	A	R	E	S	N	S	E	R	23
39	Xenopus tropicalis	Western Clawed Frog	1	M	A	A	A	I	A	S	S	L	I	R	Q	K	R	Q	A	R	E	S	N	S	D	R	23
40	Gopherus evgoodei	Goode's Thronscrub Tortoise	1	M	A	A	A	I	A	S	S	L	I	R	Q	K	R	Q	A	R	E	S	N	S	E	R	23
41	Chelonoidis abingdonii	Pinta Island Tortoise	1	M	A	A	A	I	A	S	S	L	I	R	Q	K	R	Q	A	R	E	S	N	S	E	R	23
42	Labeo rohita	Rohu	1	M	-	A	A	I	A	S	S	L	I	R	Q	K	R	Q	A	R	E	S	N	S	D	R	22
43	Denticeps clupeoides	Denticle Herring	1	M	A	A	A	I	A	S	S	L	I	R	Q	K	R	Q	A	R	E	S	N	S	D	R	23
44	Chanos chanos	Milkfish	1	M	-	A	A	I	A	S	S	L	I	R	Q	K	R	Q	A	R	E	S	N	S	D	R	22
45	Epinephelus lanceolatus	Giant Grouper	1	M	-	A	A	I	A	S	S	L	I	R	Q	K	R	Q	A	R	E	S	N	S	D	R	22
46	Paramormyrops kingsleyae	Paramormyrops kingsleyae	1	M	-	A	A	I	A	S	S	L	I	R	Q	K	R	Q	A	R	E	S	N	S	D	R	22
47	Betta splendens	Siamese Fighting Fish	1	M	-	A	A	I	A	S	S	L	I	R	Q	K	R	Q	A	R	E	S	N	S	D	R	22
48	Anabas testudineus	Climbing Perch	1	M	A	A	A	I	A	S	S	L	I	R	Q	K	R	Q	A	R	E	S	N	S	D	K	23
49	Notolabrus celidotus	Spotty	1	M	-	A	A	I	A	S	S	L	I	R	Q	K	R	Q	A	R	E	S	N	S	D	R	22
50	Oryzias melastigma	Marine Medaka	1	M	-	A	A	I	A	S	S	L	I	R	Q	K	R	Q	A	R	E	S	N	S	D	R	22
51	Oryzias latipes	Japanese Rice Fish	1	M	-	A	A	I	A	S	S	L	I	R	Q	K	R	Q	A	R	E	S	N	S	D	R	22
52	Fundulus heteroclitus	Mummichog	1	M	-	A	A	I	A	S	S	L	I	R	Q	K	R	Q	A	R	E	S	N	S	D	R	22
53	Boleophthalmus pectinirostris	Mudskipper	1	M	-	A	A	I	A	S	S	L	I	R	Q	K	R	Q	A	R	E	S	N	S	D	R	22
54	Labrus bergylta	Ballan Wrasse	1	M	-	A	A	I	A	S	S	L	I	R	Q	K	R	Q	A	R	E	S	N	S	D	R	22
55	Oreochromis niloticus	Nile Tilapia	1	M	-	A	A	I	A	S	S	L	I	R	Q	K	R	Q	A	R	E	S	N	S	D	R	22
56	Hippoglossus hippoglossus	Atlantic Halibut	1	M	-	A	A	I	A	S	S	L	I	R	Q	K	R	Q	A	R	E	S	N	S	D	R	22

Table S3

(*Related to Fig. 2*)

Comparison of FGF13A LTP Sequences From 63 Species

LTP Sequence Table (Completely Conserved Positions Highlighted in Yellow, Positions Conserved in ≥ 90% of Species Highlighted in Green)

				M		A	A	I	A	S		L	I	R	Q	K	R	Q		R	E	R	E	K	S	N	
	**Species**	**Common Name**	**Start Residue**	1	2	3	4	5	6	7	8	9	10	11	12	13	14	15	16	17	18	19	20	21	22	23	**End Residue**
1	Loxodonta africana	African Bush Elephant	1	M	A	A	A	I	A	S	S	L	I	R	Q	K	R	Q	A	R	E	R	E	K	S	N	23
2	Vicugna pacos	Alpaca	1	M	A	A	A	I	A	S	S	L	I	R	Q	K	R	Q	A	R	E	R	E	K	S	N	23
3	Urocitellus parryii	Arctic Ground Squirrel	1	M	A	A	A	I	A	S	S	L	I	R	Q	K	R	Q	A	R	E	R	E	K	S	N	23
4	Alaudala cheleensis	Asian Short-Toed Lark	1	M	A	A	A	I	A	S	S	L	I	R	Q	K	R	Q	A	R	E	R	E	K	S	N	23
5	Serinus canaria	Atlantic Canary	1	M	A	A	A	I	A	S	S	L	I	R	Q	K	R	Q	A	R	E	R	E	K	S	N	23
6	Hirundo rustica	Barn Swallow	1	M	A	A	A	I	A	S	S	L	I	R	Q	K	R	Q	A	R	E	R	E	K	S	N	23
7	Limosa lapponica baueri	Bar-tailed Godwit	1	M	A	A	A	I	A	S	S	L	I	R	Q	K	R	Q	A	R	E	R	E	K	S	N	23
8	Rattus rattus	Black Rat	1	M	A	A	A	I	A	S	S	L	I	R	Q	K	R	Q	A	R	E	R	E	K	S	N	23
9	Myotis brandtii	Brandt's Bat	1	M	A	A	A	I	A	S	S	L	I	R	Q	K	R	Q	A	R	E	R	E	K	S	N	23
10	Python bivittatus	Burmese Python	1	M	A	A	A	I	A	S	S	L	I	R	Q	K	R	Q	A	R	E	R	E	K	S	N	23
11	Bos taurus	Cattle	1	M	A	A	A	I	A	S	S	L	I	R	Q	K	R	Q	A	R	E	R	E	K	S	N	23
12	Alligator sinensis	Chinese Alligator	1	M	A	A	A	I	A	S	S	L	I	R	Q	K	R	Q	A	R	E	R	E	K	S	N	23
13	Macaca fascicularis	Crab-eating Macaque	1	M	A	A	A	I	A	S	S	L	I	R	Q	K	R	Q	A	R	E	R	E	K	S	N	23
14	Myotis davidii	David's Myotis	1	M	A	A	A	I	A	S	S	L	I	R	Q	K	R	Q	A	R	E	R	E	K	S	N	23
15	Gopherus evgoodei	Desert Tortoise	1	M	A	A	A	I	A	S	S	L	I	R	Q	K	R	Q	A	R	E	R	E	K	S	N	23
16	Canis lupus familiaris	Dog	1	M	A	A	A	I	A	S	S	L	I	R	Q	K	R	Q	A	R	E	R	E	K	S	N	23
17	Camelus dromedarius	Dromedary	1	M	A	A	A	I	A	S	S	L	I	R	Q	K	R	Q	A	R	E	R	E	K	S	N	23
18	Pseudonaja textilis	Eastern Brown Snake	1	M	A	A	A	I	A	S	S	L	I	R	Q	K	R	Q	A	R	E	R	E	K	S	N	23
19	Erithacus rubecula	European Robin	1	M	A	A	A	I	A	S	S	L	I	R	Q	K	R	Q	A	R	E	R	E	K	S	N	23
20	Geotrypetes seraphini	Gaboon Caecilian	1	M	A	A	A	I	A	S	S	L	I	R	Q	K	R	Q	A	R	E	R	E	K	S	N	23
21	Mesocricetus auratus	Golden Hamster	1	M	A	A	A	I	A	S	S	L	I	R	Q	K	R	Q	A	R	E	R	E	K	S	N	23
22	Acrocephalus arundinaceus	Great Reed Warbler	1	M	A	A	A	I	A	S	S	L	I	R	Q	K	R	Q	A	R	E	R	E	K	S	N	23
23	Rhinolophus ferrumequinum	Greater Horseshoe Bat	1	M	A	A	A	I	A	S	S	L	I	R	Q	K	R	Q	A	R	E	R	E	K	S	N	23
24	Chelonia mydas	Green Sea Turtle	1	M	A	A	A	I	A	S	S	L	I	R	Q	K	R	Q	A	R	E	R	E	K	S	N	23
25	Cavia porcellus	Guinea Pig	1	M	A	A	A	I	A	S	S	L	I	R	Q	K	R	Q	A	R	E	R	E	K	S	N	23
26	Mus musculu	House Mouse	1	M	T	A	A	I	A	S	S	L	I	R	Q	K	R	Q	A	R	E	R	E	K	S	N	23
27	Homo sapiens	Human	1	M	A	A	A	I	A	S	S	L	I	R	Q	K	R	Q	A	R	E	R	E	K	S	N	23
28	Cercotrichas coryphoeus	Karoo Scrub Robin	1	M	A	A	A	I	A	S	S	L	I	R	Q	K	R	Q	A	R	E	R	E	K	S	N	23
29	Phascolarctos cinereus	Koala	1	M	A	A	A	I	A	S	S	L	I	R	Q	K	R	Q	A	R	E	R	E	K	S	N	23
30	Turnix velox	Little Buttonquail	1	M	A	A	A	I	A	S	S	L	I	R	Q	K	R	Q	A	R	E	R	E	K	S	N	23
31	Mionectes macconnelli	McConnell's Flycatcher	1	M	A	A	A	I	A	S	S	L	I	R	Q	K	R	Q	A	R	E	R	E	K	S	N	23
32	Suricata suricatta	Meerkat	1	M	A	A	A	I	A	S	S	L	I	R	Q	K	R	Q	A	R	E	R	E	K	S	N	23
33	Meriones unguiculatus	Mongolian Gerbil	1	M	A	A	A	I	A	S	S	L	I	R	Q	K	R	Q	A	R	E	R	E	K	S	N	23
34	Heterocephalus glaber	Naked Mole-rat	1	M	A	A	A	I	A	S	S	L	I	R	Q	K	R	Q	A	R	E	R	E	K	S	N	23
35	Otolemur garnettii	Northern Greater Galago	1	M	A	A	A	I	A	S	S	L	I	R	Q	K	R	Q	A	R	E	R	E	K	S	N	23
36	Papio anubis	Olive Baboon	6	M	A	A	A	I	A	S	S	L	I	R	Q	K	R	Q	A	R	E	R	E	K	S	N	23
37	Chloropsis hardwickii	Orange-bellied Leafbird	1	M	A	A	A	I	A	S	S	L	I	R	Q	K	R	Q	A	R	E	R	E	K	S	N	23
38	Phainopepla nitens	Phainopepla	1	M	A	A	A	I	A	S	S	L	I	R	Q	K	R	Q	A	R	E	R	E	K	S	N	23
39	Pedionomus torquatus	Plains-wanderer	1	M	A	A	A	I	A	S	S	L	I	R	Q	K	R	Q	A	R	E	R	E	K	S	N	23
40	Ornithorhynchus anatinus	Platypus	1	M	A	A	A	I	A	S	S	L	I	R	Q	K	R	Q	A	R	E	R	E	K	S	N	23
41	Protobothrops mucrosquamatus	Pointed Scale Viper	1	M	A	A	A	I	A	S	S	L	I	R	Q	K	R	Q	A	R	E	R	E	K	S	N	23
42	Todus mexicanus	Puerto Rican Tody	1	M	A	A	A	I	A	S	S	L	I	R	Q	K	R	Q	A	R	E	R	E	K	S	N	23
43	Gallus gallus	Red Junglefowl	1	M	A	A	A	I	A	S	S	L	I	R	Q	K	R	Q	A	R	E	R	E	K	S	N	23
44	Malurus elegans	Red-winged Fairywren	1	M	A	A	A	I	A	S	S	L	I	R	Q	K	R	Q	A	R	E	R	E	K	S	N	23
45	Macaca mulatta	Rhesus Macaque	6	M	A	A	A	I	A	S	S	L	I	R	Q	K	R	Q	G	R	E	R	E	K	S	N	23
46	Rhinatrema bivittatum	Rhinatrema bivittatum	1	M	A	A	A	I	A	S	S	L	I	R	Q	K	R	Q	A	R	E	R	E	K	S	N	23
47	Origma solitaria	Rockwarbler	1	M	A	A	A	I	A	S	S	L	I	R	Q	K	R	Q	A	R	E	R	E	K	S	N	23
48	Turdus rufiventris	Rufous-bellied thrush	1	M	A	A	A	I	A	S	S	L	I	R	Q	K	R	Q	A	R	E	R	E	K	S	N	23
49	Xiphophorus maculatus	Southern Platyfish	1	M	A	A	A	I	A	S	S	L	I	R	Q	K	R	Q	A	R	E	R	E	K	S	N	23
50	Drymodes brunneopygia	Southern Scrub Robin	1	M	A	A	A	I	A	S	S	L	I	R	Q	K	R	Q	A	R	E	R	E	K	S	N	23
51	Acipenser ruthenus	Sterlet	1	M	A	A	A	I	A	S	A	L	I	R	Q	K	R	Q	A	R	E	R	E	K	S	N	23
52	Pongo abelii	Sumatran Orangutan	1	M	A	A	A	I	A	S	S	L	I	R	Q	K	R	Q	A	R	E	R	E	K	S	N	23
53	Catharus ustulatus	Swainson's Thrush	1	M	A	A	A	I	A	S	S	L	I	R	Q	K	R	Q	A	R	E	R	E	K	S	N	23
54	Notechis Scutatus	Tiger Snake	1	M	A	A	A	I	A	S	S	L	I	R	Q	K	R	Q	A	R	E	R	E	K	S	N	23
55	Thalassophryne amazonica	Toadfish	1	M	A	A	A	I	A	S	S	L	I	R	Q	K	R	Q	A	R	E	R	E	K	S	N	23
56	Tupaia chinensis	Treeshrew	1	M	A	A	A	I	A	S	S	L	I	R	Q	K	R	Q	A	R	E	R	E	K	S	N	23
57	Podarcis muralis	Wall Lizard	1	M	A	A	A	I	A	S	S	L	I	R	Q	K	R	Q	A	R	E	R	E	K	S	N	23
58	Xenopus tropicalis	Western Clawed Frog	1	M	A	A	A	I	A	S	S	L	I	R	Q	K	R	Q	A	R	E	R	E	K	S	N	23
59	Nicator chloris	Western Nicator	1	M	A	A	A	I	A	S	S	L	I	R	Q	K	R	Q	A	R	E	R	E	K	S	N	23
60	Chrysemys picta bellii	Western Painted Turtle	1	M	A	A	A	I	A	S	S	L	I	R	Q	K	R	Q	A	R	E	R	E	K	S	N	23
61	Thamnophis elegans	Western Terrestrial Garter Snake	1	M	A	A	A	I	A	S	S	L	I	R	Q	K	R	Q	A	R	E	R	E	K	S	N	23
62	Sus scrofa	Wild Boar	1	M	A	A	A	I	A	S	S	L	I	R	Q	K	R	Q	A	R	E	R	E	K	S	N	23
63	Taeniopygia guttata	Zebra Finch	1	M	A	A	A	I	A	S	S	L	I	R	Q	K	R	Q	A	R	E	R	E	K	S	N	23

Table S4
(Related to Fig. 2)
Comparison of FGF14A LTP Sequences From 48 Species

LTP Sequence Table (Completely Conserved Positions Highlighted in Yellow, Positions Conserved in ≥ 90% of Species Highlighted in Green)

	Species	Common Name	Start Residue	1	2	3	4	5	6	7	8	9	10	11	12	13	14	15	16	17	18	19	20	21	22	23	End Residue
				M	A	A	A	I	A	S	G	L	I	R	Q	K	R	Q	A	R	E	Q			D	R	
1	Calypte anna	Anna's Hummingbird	1	M	A	A	A	I	A	S	G	L	I	R	Q	K	R	Q	A	R	E	Q	H	W	D	R	23
2	Scleropages formosus	Asian Arowana	1	M	A	A	A	I	A	S	G	L	I	R	Q	K	R	Q	A	R	E	Q	H	I	D	R	23
3	Monopterus albus	Asian Swamp Eel	1	M	A	A	A	I	A	S	G	L	I	R	Q	K	R	Q	A	R	E	Q	H	L	D	R	23
4	Alectura lathami	Australian Brushturkey	1	M	A	A	A	I	A	S	G	L	I	R	Q	K	R	Q	A	R	E	Q	H	W	D	R	23
5	Limosa lapponica baueri	Bar-tailed Godwit	1	M	A	A	A	I	A	S	G	L	I	R	Q	K	R	Q	A	R	E	Q	H	W	D	R	23
6	Pteropus alecto	Black Flying Fox	1	M	A	A	A	I	A	S	G	L	I	R	Q	K	R	Q	A	R	E	Q	H	W	D	R	23
7	Rattus rattus	Black Rat	1	M	A	A	A	I	A	S	G	L	I	R	Q	K	R	Q	A	R	E	Q	H	W	D	R	23
8	Felis catus	Cat	1	M	A	A	A	I	A	S	G	L	I	R	Q	K	R	Q	A	R	E	Q	H	W	D	R	23
9	Cricetulus griseus	Chinese Hamster	1	M	A	A	A	I	A	S	G	L	I	R	Q	K	R	Q	A	R	E	Q	H	W	D	R	23
10	Desmodus rotundus	Common Vampire Bat	1	M	A	A	A	I	A	S	G	L	I	R	Q	K	R	Q	A	R	E	Q	H	W	D	R	23
11	Cottoperca gobio	Cottoperca	1	M	A	A	A	I	A	S	G	L	I	R	Q	K	R	Q	A	R	E	Q	H	L	D	R	23
12	Cynoglossus semilaevis	Cynoglossus semilaevis	1	M	A	A	A	I	A	S	G	L	I	R	Q	K	R	Q	A	R	E	Q	H	L	D	R	23
13	Denticeps clupeoides	Denticle Herring	1	M	A	A	A	I	A	S	G	L	I	R	Q	K	R	Q	A	R	E	Q	H	L	D	R	23
14	Canis lupus dingo	Dingo	1	M	A	A	A	I	A	S	G	L	I	R	Q	K	R	Q	A	R	E	Q	H	W	D	R	23
15	Camelus dromedarius	Dromedary	1	M	A	A	A	I	A	S	G	L	I	R	Q	K	R	Q	A	R	E	Q	T	W	D	R	23
16	Electrophorus electricus	Electric Eel	1	M	A	A	A	I	A	S	G	L	I	R	Q	K	R	Q	A	R	E	Q	H	L	D	R	23
17	Anguilla anguilla	European Eel	1	M	A	A	A	I	A	S	G	L	I	R	Q	K	R	Q	A	R	E	Q	H	V	D	R	23
18	Geotrypetes seraphini	Gaboon Caecilian	1	M	A	A	A	I	A	S	G	L	I	R	Q	K	R	Q	A	R	E	Q	H	L	D	R	23
19	Aquila chrysaetos chrysaetos	Golden Eagle	1	M	A	A	A	I	A	S	G	L	I	R	Q	K	R	Q	A	R	E	Q	H	W	D	R	23
20	Microcebus murinus	Gray Mouse Lemur	1	M	A	A	A	I	A	S	G	L	I	R	Q	K	R	Q	A	R	E	Q	H	W	D	R	23
21	Mus musculus	House Mouse	1	M	A	A	A	I	A	S	G	L	I	R	Q	K	R	Q	A	R	E	Q	H	W	D	R	23
22	Homo sapiens	Human	1	M	A	A	A	I	A	S	G	L	I	R	Q	K	R	Q	A	R	E	Q	H	W	D	R	23
23	Clarias magur	Indian Catfish	1	M	A	A	A	I	A	S	G	L	I	R	Q	K	R	Q	A	R	E	Q	H	L	D	R	23
24	Oryzias latipes	Japanese Rice Fish	1	M	A	A	A	I	A	S	G	L	I	R	Q	K	R	Q	A	R	E	Q	H	L	D	R	23
25	Trichechus manatus latirostris	Manatee	1	M	A	A	A	I	A	S	G	L	I	R	Q	K	R	Q	A	R	E	Q	H	W	D	R	23
26	Microcaecilia unicolor	Microcaecilia unicolor	1	M	A	A	A	I	A	S	G	L	I	R	Q	K	R	Q	A	R	E	Q	H	L	D	R	23
27	Chanos chanos	Milkfish	1	M	A	A	A	I	A	S	G	L	I	R	Q	K	R	Q	A	R	E	Q	H	I	D	R	23
28	Fundulus heteroclitus	Mummichog	1	M	A	A	A	I	A	S	G	L	I	R	Q	K	R	Q	A	R	E	Q	H	L	D	R	23
29	Dasypus novemcinctus	Nine-banded Armadillo	1	M	A	A	A	I	A	S	G	L	I	R	Q	K	R	Q	A	R	E	Q	H	W	D	R	23
30	Paramormyrops kingsleyae	Paramaormyrops	1	M	A	A	A	I	A	S	G	L	I	R	Q	K	R	Q	A	R	E	Q	H	I	D	R	23
31	Chelonoidis abingdonii	Pinta Island Tortoise	1	M	A	A	A	I	A	S	G	L	I	R	Q	K	R	Q	A	R	E	Q	H	W	D	R	23
32	Microtus ochrogaster	Prairie Vole	1	M	A	A	A	I	A	S	G	L	I	R	Q	K	R	Q	A	R	E	Q	H	W	D	R	23
33	Takifugu flavidus	Pufferfish	1	M	A	A	A	I	A	S	G	L	I	R	Q	K	R	Q	A	R	E	Q	H	L	D	R	23
34	Gallus gallus	Red Junglefowl	1	M	A	A	A	I	A	S	G	L	I	R	Q	K	R	Q	A	R	E	Q	H	W	D	R	23
35	Erpetoichthys calabaricus	Reedfish	1	M	A	A	A	I	A	S	G	L	I	R	Q	K	R	Q	A	R	E	Q	H	T	D	R	23
36	Xiphophorus maculatus	Souther Platyfish	1	M	A	A	A	I	A	S	G	L	I	R	Q	K	R	Q	A	R	E	Q	H	L	D	R	23
37	Mirounga leonina	Southern Elephant Seal	1	M	A	A	A	I	A	S	G	L	I	R	Q	K	R	Q	A	R	E	Q	H	W	D	R	23
38	Nannospalax galili	Spalax	1	M	A	A	A	I	A	S	G	L	I	R	Q	K	R	Q	A	R	E	Q	H	W	D	R	23
39	Mustela erminea	Stoat	1	M	A	A	A	I	A	S	G	L	I	R	Q	K	R	Q	A	R	E	Q	Q	W	D	R	23
40	Thalassophryne amazonica	Toadfish	1	M	A	A	A	I	A	S	G	L	I	R	Q	K	R	Q	A	R	E	Q	H	F	D	R	23
41	Amazona aestiva	Turquoise-fronted Amazon	1	M	A	A	A	I	A	S	G	L	I	R	Q	K	R	Q	A	R	E	Q	H	W	D	R	23
42	Rhinatrema bivittatum	Two-tailed Caecilian	1	M	A	A	A	I	A	S	G	L	I	R	Q	K	R	Q	A	R	E	Q	H	L	D	R	23
43	Bubalus bubalis	Water Buffalo	1	M	A	A	A	I	A	S	G	L	I	R	Q	K	R	Q	A	R	E	Q	H	W	D	R	23
44	Xenopus tropicalis	Western Clawed Frog	1	M	A	A	A	I	A	S	G	L	I	R	Q	K	R	Q	A	R	E	Q	H	L	D	R	23
45	Chrysemys picta bellii	Western Painted Turtle	1	M	A	A	A	I	A	S	G	L	I	R	Q	K	R	Q	A	R	E	Q	H	W	D	R	23
46	Zonotrichia albicollis	White-throated Sparrow	1	M	A	A	A	I	A	S	G	L	I	R	Q	K	R	Q	A	R	E	Q	H	W	D	R	23
47	Sus scrofa	Wild Boar	1	M	A	A	A	I	A	S	G	L	I	R	Q	K	R	Q	A	R	E	Q	H	W	D	R	23
48	Mastacembelus armatus	Zig-zag Eel	1	M	A	A	A	I	A	S	G	L	I	R	Q	K	R	Q	A	R	E	Q	H	L	D	R	23

Table S5

(Related to Fig. 2)

Comparison of FGF11A CaMBD Sequences From 60 Species

CaMBD Sequence Table (Completely Conserved Positions Highlighted in Yellow, Positions Conserved in ≥ 90% of Species Highlighted in Green)

				K	S	L	C	Q	K	Q				L		S	K	V	R	L	C			R							
	Species	Common Name	Start Residue	36	37	38	39	40	41	42	43	44	45	46	47	48	49	50	51	52	53	54	55	56	57	58	59	60	61	62	End Residue
1	Homo sapiens	Human	36	K	S	L	C	Q	K	Q	L	L	I	L	L	S	K	V	R	L	C	G	G	R	P	A	R	P	D	R	62
2	Danio rerio	Zebrafish	36	K	S	L	C	Q	K	Q	I	L	I	L	I	S	K	V	R	L	C	G	S	R	G	R	K	L	E	K	62
3	Pangasianodon hypophthalmus	Iridescent shark	36	K	S	L	C	Q	K	Q	I	L	I	L	I	S	K	V	R	L	C	G	S	R	G	R	K	L	E	K	62
4	Anarrhichthys ocellatus	Wolf eel	36	K	S	L	C	Q	K	Q	I	L	I	L	I	S	K	V	R	L	C	G	S	R	G	R	K	L	E	K	62
5	Myripristis murdjan	Soldierfish	36	K	S	L	C	Q	K	Q	I	L	I	L	I	S	K	V	R	L	C	G	S	R	G	R	K	M	E	K	62
6	Hippoglossus hippoglossus	Righteye Founder	36	K	S	L	C	Q	K	Q	I	L	I	L	I	S	K	V	R	L	C	G	S	R	G	R	K	L	E	K	62
7	Oreochromis aureus	Blue Tilpia	36	K	S	L	C	Q	K	Q	I	L	I	L	I	S	K	V	R	L	C	G	S	R	G	R	K	L	E	K	62
8	Denticeps clupeoides	Denticle Herring	36	K	S	L	C	Q	K	Q	I	L	I	L	I	S	K	V	R	L	C	G	S	R	G	R	K	L	E	K	62
9	Astyanax mexicanus	Mexican Tetra	36	K	S	L	C	Q	K	Q	I	L	I	L	I	S	K	V	R	L	C	G	S	R	G	R	K	L	E	K	62
10	Cyprinus carpio	Carp	36	K	S	L	C	Q	K	Q	I	L	I	L	I	S	K	V	R	L	C	G	S	R	G	R	K	L	V	K	62
11	Clupea harengus	Atlantic Herring	36	K	S	L	C	Q	K	Q	I	L	I	L	I	S	K	V	R	L	C	G	S	R	G	R	K	L	E	K	62
12	Scophthalmus maximus	Turbot	36	K	S	L	C	Q	K	Q	I	L	I	L	I	S	K	V	R	L	C	G	S	R	G	R	K	M	E	K	62
13	Microcebus murinus	Gray Mouse Lemur	36	K	S	L	C	Q	K	Q	L	L	I	L	L	S	K	V	R	L	C	G	G	R	P	A	R	P	D	R	62
14	Vicugna pacos	Alpaca	36	K	S	L	C	Q	K	Q	L	L	I	L	L	S	K	V	R	L	C	G	G	R	P	A	R	P	D	R	62
15	Macaca mulatta	Rhesus Maceque	36	K	S	L	C	Q	K	Q	L	L	I	L	L	S	K	V	R	L	C	G	G	R	P	A	R	P	D	R	62
16	Papio anubis	Olive Baboon	36	K	S	L	C	Q	K	Q	L	L	I	L	L	S	K	V	R	L	C	G	G	R	P	A	R	P	D	R	62
17	Trachypithecus francoisi	Tonkin leaf monkey	36	K	S	L	C	Q	K	Q	L	L	I	L	L	S	K	V	R	L	C	G	G	R	P	A	R	P	D	R	62
18	Castor canadensis	North American Beaver	36	K	S	L	C	Q	K	Q	L	L	I	L	L	S	K	V	R	L	C	G	G	R	P	A	R	P	D	R	62
19	Rhinopithecus roxellana	Golden snub-nosed monkey	36	K	S	L	C	Q	K	Q	L	L	I	L	L	S	K	V	R	L	C	G	G	R	P	A	R	P	D	R	62
20	Tupaia chinensis	Treeshrew	36	K	S	L	C	Q	K	Q	L	L	I	L	L	S	K	V	R	L	C	G	G	R	P	A	R	P	D	R	62
21	Carlito syrichta	Philippine Tarsier	36	K	S	L	C	Q	K	Q	L	L	I	L	L	S	K	V	R	L	C	G	G	R	P	A	R	P	D	R	62
22	Canis lupus familiaris	Dog	36	K	S	L	C	Q	K	Q	L	L	I	L	L	S	K	V	R	L	C	G	G	R	P	A	R	P	D	R	62
23	Crocuta crocuta	Spotted Hyena	36	K	S	L	C	Q	K	Q	L	V	I	L	L	S	K	V	R	L	C	G	G	R	P	A	R	P	D	R	62
24	Canis lupus dingo	Dingo	36	K	S	L	C	Q	K	Q	L	L	I	L	L	S	K	V	R	L	C	G	G	R	P	A	R	P	D	R	62
25	Sapajus apella	Tuffed Capuchin	36	K	S	L	C	Q	K	Q	L	L	I	L	L	S	K	V	R	L	C	G	G	R	P	A	R	P	D	R	62
26	Aotus nancymaae	Night Monkey	36	K	S	L	C	Q	K	Q	L	L	I	L	L	S	K	V	R	L	C	G	G	R	P	A	R	P	D	R	62
27	Felis catus	Cat	36	K	S	L	C	Q	K	Q	L	V	I	L	L	S	K	V	R	L	C	G	G	R	P	A	R	P	D	R	62
28	Zalophus californianus	California Sea Lion	36	K	S	L	C	Q	K	Q	L	L	I	L	L	S	K	V	R	L	C	G	G	R	P	A	R	P	D	R	62
29	Nomascus leucogenys	Northern White-Cheeked Gibbon	36	K	S	L	C	Q	K	Q	L	L	I	L	L	S	K	V	R	L	C	G	G	R	P	A	R	P	D	R	62
30	Callorhinus ursinus	Northern Fur Seal	36	K	S	L	C	Q	K	Q	L	L	I	L	L	S	K	V	R	L	C	G	G	R	P	A	R	P	D	R	62
31	Otolemur garnettii	Northern Greater Galago	36	K	S	L	C	Q	K	Q	L	L	I	L	L	S	K	V	R	L	C	G	G	R	P	T	R	P	D	R	62
32	Pteropus alecto	Black Flying Fox	36	K	S	L	C	Q	K	Q	L	L	I	L	L	S	K	V	R	L	C	G	G	R	P	A	R	P	N	R	62
33	Suricata suricatta	Meerkat	36	K	S	L	C	Q	K	Q	L	V	I	L	L	S	K	V	R	L	C	G	G	R	P	S	R	P	D	R	62
34	Dasypus novemcinctus	None-banded Armadillo	36	K	S	L	C	Q	K	Q	L	L	V	L	L	S	K	V	R	L	C	G	G	R	P	S	R	P	D	R	62
35	Bos taurus	Cattle	36	K	S	L	C	Q	K	Q	L	L	I	L	L	S	K	V	R	L	C	G	G	R	P	A	R	T	D	R	62
36	Phyllostomus discolor	Pale Spear-nosed Bat	36	K	S	L	C	Q	K	Q	L	L	I	L	L	S	K	V	R	L	C	G	G	R	P	G	R	P	D	H	62
37	Hylobates moloch	Silvery Gibbon	36	K	S	L	C	Q	K	Q	L	L	I	L	L	S	K	V	R	L	C	G	G	R	P	P	R	P	D	R	62
38	Pongo abelii	Sumatran Orangutan	36	K	S	L	C	Q	K	Q	L	L	I	L	L	S	K	V	R	L	C	G	G	R	P	A	L	P	D	R	62
39	Heterocephalus glaber	Naked mole-rat	36	K	S	L	C	Q	K	Q	L	L	I	L	L	S	K	V	R	L	C	G	G	R	P	A	P	P	D	R	62
40	Equus caballus	Horse	36	K	S	L	C	Q	K	Q	L	L	I	L	L	S	K	V	R	L	C	G	G	R	P	A	R	P	D	R	62
41	Ovis aries	Sheep	36	K	S	L	C	Q	K	Q	L	L	I	L	L	S	K	V	R	L	C	G	G	R	P	A	R	T	D	R	62
42	Eptesicus fuscus	Big Brown Bat	36	K	S	L	C	Q	K	Q	L	L	I	L	L	S	K	V	R	L	C	G	G	R	P	A	R	L	D	R	62
43	Leptonychotes weddellii	Weddell Seal	36	K	S	L	C	Q	K	Q	L	L	I	L	L	S	K	V	R	L	C	G	G	R	P	A	R	L	D	R	62
44	Octodon degus	Common Degu	36	K	S	L	C	Q	K	Q	L	L	I	L	L	S	K	V	R	L	C	G	G	R	P	A	R	P	D	R	62
45	Gorilla gorilla gorilla	Gorilla	36	K	S	L	C	Q	K	Q	L	L	I	L	L	S	K	V	R	L	C	R	G	R	P	A	R	P	D	R	62
46	Cavia porcellus	Guinea Pig	36	K	S	L	C	Q	K	Q	L	L	I	L	L	S	K	V	R	L	C	G	G	R	P	A	R	P	D	R	62
47	Callithrix jacchus	Common Marmoset	36	K	S	L	C	Q	K	Q	L	L	I	L	L	S	K	V	R	L	C	G	G	R	P	A	R	P	D	G	62
48	Mus pahari	Gairdner's Shrewmouse	36	K	S	L	C	Q	K	Q	L	L	I	L	L	S	K	V	R	L	C	G	G	R	P	T	R	Q	D	R	62
49	Mirounga leonina	Southern Elepant Seal	36	K	S	L	C	Q	K	Q	L	L	I	L	L	S	K	V	R	L	C	G	G	R	P	A	R	L	D	R	62
50	Mus musculus	House Mouse	36	K	S	L	C	Q	K	Q	L	L	I	L	L	S	K	V	R	L	C	G	G	R	P	T	R	Q	D	R	62
51	Ailuropoda melanoleuca	Giant Panda	36	K	S	L	C	Q	K	Q	L	L	V	L	L	S	K	V	R	L	C	G	G	R	P	A	R	P	A	R	62
52	Grammomys surdaster	African Tree Rat	36	K	S	L	C	Q	K	Q	L	L	I	L	L	S	K	V	R	L	C	G	G	R	P	T	R	Q	D	R	62
53	Ursus arctos horribilis	Grizzly Bear	36	K	S	L	C	Q	K	Q	L	L	V	L	L	S	K	V	R	L	C	G	G	R	P	A	R	P	A	R	62
54	Nannospalax galili	Blind Mole-rat	36	K	S	L	C	Q	K	Q	L	L	I	L	L	S	K	V	R	L	C	G	G	R	P	T	R	Q	D	R	62
55	Rattus norvegicus	Brown Rat	36	K	S	L	C	Q	K	Q	L	L	I	L	L	S	K	V	R	L	C	G	G	R	P	T	R	Q	D	R	62
56	Desmodus rotundus	Vampire Bat	36	K	S	L	C	Q	K	Q	L	L	I	L	L	S	K	V	R	L	C	G	G	R	S	G	R	P	D	R	62
57	Meriones unguiculatus	Mongolian Gerbil	36	K	S	L	C	Q	K	Q	L	L	I	L	L	S	K	V	R	L	C	G	G	R	P	T	R	Q	D	R	62
58	Mus caroli	Ryukyu Mouse	36	K	S	L	C	Q	K	Q	L	L	I	L	L	S	K	V	R	L	C	G	G	R	P	T	R	Q	D	R	62
59	Urocitellus parryii	Arctic Ground Squirrel	36	K	S	L	C	Q	K	Q	L	L	I	L	L	S	K	V	R	L	C	G	G	R	P	T	R	P	D	R	62
60	Lontra canadensis	North American River Otter	36	K	S	L	C	Q	K	Q	L	L	I	L	L	S	K	V	R	L	C	G	G	R	P	S	R	P	D	R	62

Table S6

(*Related to Fig. 2)*

Comparison of FGF12A CaMBD Sequences From 56 Species

CaMBD Sequence Table (Completely Conserved Positions Highlighted in Yellow, Positions Conserved in ≥ 90% of Species Highlighted in Green)

	Species	Common Name	Start Residue	38	39	40	41	42	43	44	45	46	47	48	49	50	51	52	53	54	55	56	57	58	59	60	61	62	63	64	End Residue
				R	S	L	C	E	R	H				V	F	S	K	V	R	F	C				K			V	R	R	
1	Homo sapiens	Human	38	R	S	L	C	E	R	H	V	L	G	V	F	S	K	V	R	F	C	S	G	R	K	R	P	V	R	R	64
2	Danio rerio	Zebrafish	37	R	S	L	C	E	R	H	F	L	G	V	F	S	K	V	R	F	C	S	G	K	K	R	P	V	R	R	63
3	Apteryx rowi	Kiwi	38	R	S	L	C	E	R	H	V	L	G	V	F	S	K	V	R	F	C	S	G	R	K	R	P	V	R	R	64
4	Castor canadensis	North American Beaver	38	R	S	L	C	E	R	H	V	L	G	V	F	S	K	V	R	F	C	S	G	R	K	R	P	V	R	R	64
5	Cavia porcellus	Guinea Pig	38	R	S	L	C	E	R	H	V	L	G	V	F	S	K	V	R	F	C	S	G	R	K	R	P	V	R	R	64
6	Cricetulus griseus	Chinese Hamster	38	R	S	L	C	E	R	H	V	L	G	V	F	S	K	V	R	F	C	S	G	R	K	R	P	V	R	R	64
7	Cyprinus carpio	Common Carp	38	R	S	L	C	E	R	H	F	L	G	V	F	S	K	V	R	F	C	S	G	K	K	I	P	V	R	R	64
8	Fukomys damarensis	Damaraland Mole-Rat	38	R	S	L	C	E	R	H	V	L	G	V	F	S	K	V	R	F	C	S	G	R	K	R	P	V	R	R	64
9	Gallus gallus	Red Junglefowl	38	R	S	L	C	E	R	H	V	L	G	V	F	S	K	V	R	F	C	S	G	R	K	R	P	V	R	R	64
10	Lynx pardinus	Iberian Lynx	38	R	S	L	C	E	R	H	V	L	G	V	F	S	K	V	R	F	C	S	G	R	K	R	P	V	R	R	64
11	Macaca mulatta	Rhesus Macaque	38	R	S	L	C	E	R	H	V	L	G	V	F	S	K	V	R	F	C	S	G	R	K	R	P	V	R	R	64
12	Mirounga leonina	Southern Elephant Seal	38	R	S	L	C	E	R	H	V	L	G	V	F	S	K	V	R	F	C	S	G	R	K	R	P	V	R	R	64
13	Myotis davidii	Vesper Bat	38	R	S	L	C	E	R	H	V	L	G	V	F	S	K	V	R	F	C	S	G	R	K	R	P	V	R	R	64
14	Myotis lucifugus	Little Brown Bat	38	R	S	L	C	E	R	H	V	L	G	V	F	S	K	V	R	F	C	S	G	R	K	R	P	V	R	R	64
15	Rattus norvegicus	Brown Rat	38	R	S	L	C	E	R	H	V	L	G	V	F	S	K	V	R	F	C	S	G	R	K	R	P	V	R	R	64
16	Spheniscus humboldti	Humboldt Penguin	38	R	S	L	C	E	R	H	V	L	G	V	F	S	K	V	R	F	C	S	G	R	K	R	P	V	R	R	64
17	Thamnophis elegans	Western Terrestrial Garter Snake	38	R	S	L	C	E	R	H	V	L	G	V	F	S	K	V	R	F	C	S	G	R	K	R	H	V	R	R	64
18	Todus mexicanus	Puetro Rican Tody	38	R	S	L	C	E	R	H	V	L	G	V	F	S	K	V	R	F	C	S	G	R	K	R	P	V	R	R	64
19	Mus musculus	House Mouse	38	R	S	L	C	E	R	H	V	L	G	V	F	S	K	V	R	F	C	S	G	R	K	R	P	V	R	R	64
20	Arvicanthis niloticus	African grass rat	38	R	S	L	C	E	R	H	V	L	G	V	F	S	K	V	R	F	C	S	G	R	K	R	P	V	R	R	64
21	Phocoena sinus	Porpoise	38	R	S	L	C	E	R	H	V	L	G	V	F	S	K	V	R	F	C	S	G	R	K	R	P	V	R	R	64
22	Camelus dromedarius	Arabian Camel	38	R	S	L	C	E	R	H	V	L	G	V	F	S	K	V	R	F	C	S	G	R	K	R	P	V	R	R	64
23	Nannospalax galili	Mole-rat	38	R	S	L	C	E	R	H	V	L	G	V	F	S	K	V	R	F	C	S	G	R	K	R	P	V	R	R	64
24	Camelus ferus	Bactrian Camel	38	R	S	L	C	E	R	H	V	L	G	V	F	S	K	V	R	F	C	S	G	R	K	R	P	V	R	R	64
25	Rhinolophus ferrumequinum	Horseshoe Bat	38	R	S	L	C	E	R	H	V	L	G	V	F	S	K	V	R	F	C	S	G	R	K	R	P	V	R	R	64
26	Trichechus manatus latirostris	Florida Manatee	38	R	S	L	C	E	R	H	V	L	G	V	F	S	K	V	R	F	C	S	G	R	K	R	P	V	R	R	64
27	Lonchura striata domestica	Society Finch	38	R	S	L	C	E	R	H	V	L	G	V	F	S	K	V	R	F	C	S	G	R	K	R	P	V	R	R	64
28	Tupaia chinensis	Tree Shrew	38	R	S	L	C	E	R	H	V	L	G	V	F	S	K	V	R	F	C	S	G	R	K	R	P	V	R	R	64
29	Dasypus novemcinctus	Nine-Banded Armadillo	38	R	S	L	C	E	R	H	V	L	G	V	F	S	K	V	R	F	C	S	G	R	K	R	P	V	R	R	64
30	Ornithorhynchus anatinus	Platypus	38	R	S	L	C	E	R	H	V	L	G	V	F	S	K	V	R	F	C	S	G	R	K	R	P	V	R	R	64
31	Camarhynchus parvulus	Small Tree Finch	38	R	S	L	C	E	R	H	V	L	G	V	F	S	K	V	R	F	C	S	G	R	K	R	P	V	R	R	64
32	Dromaius novaehollandiae	Emu	38	R	S	L	C	E	R	H	V	L	G	V	F	S	K	V	R	F	C	S	G	R	K	R	P	V	R	R	64
33	Microcaecilia unicolor	Microcaecilia unicolor	38	R	S	L	C	E	R	H	V	L	G	V	F	S	K	V	R	F	C	S	G	R	K	R	P	V	R	R	64
34	Podarcis muralis	Wall Lizard	38	R	S	L	C	E	R	H	V	L	G	V	F	S	K	V	R	F	C	S	G	R	K	R	H	V	R	R	64
35	Geotrypetes seraphini	Gaboon Caecilian	38	R	S	L	C	E	R	H	V	L	G	V	F	S	K	V	R	F	C	N	G	R	K	K	P	V	R	R	64
36	Chrysemys picta bellii	Western Painted Turtle	38	R	S	L	C	E	R	H	V	L	G	V	F	S	K	V	R	F	C	S	G	R	K	R	P	V	R	R	64
37	Terrapene carolina triunguis	Three-Toed Box Turtle	38	R	S	L	C	E	R	H	V	L	G	V	F	S	K	V	R	F	C	S	G	R	K	R	P	V	R	R	64
38	Notechis scutatus	Tiger Snake	38	R	S	L	C	E	R	H	V	L	G	V	F	S	K	V	R	F	C	S	G	R	K	R	H	V	R	R	64
39	Xenopus tropicalis	Western Clawed Frog	38	R	S	L	C	E	R	H	V	L	G	V	F	S	K	V	R	F	C	S	G	R	K	R	P	V	R	R	64
40	Gopherus evgoodei	Goode's Thronscrub Tortoise	38	R	S	L	C	E	R	H	V	L	G	V	F	S	K	V	R	F	C	S	G	R	K	R	P	V	R	R	64
41	Chelonoidis abingdonii	Pinta Island Tortoise	38	R	S	L	C	E	R	H	V	L	G	V	F	S	K	V	R	F	C	S	G	R	K	R	P	V	R	R	64
42	Labeo rohita	Rohu	37	R	S	L	C	E	R	H	F	L	G	V	F	S	K	V	R	F	C	S	G	K	K	R	P	V	R	R	63
43	Denticeps clupeoides	Denticle Herring	38	R	S	L	C	E	R	H	I	F	G	V	F	S	K	V	R	F	C	S	G	K	K	R	P	V	R	R	64
44	Chanos chanos	Milkfish	37	R	S	L	C	E	R	H	F	L	G	V	F	S	K	V	R	F	C	S	G	K	K	R	P	V	R	R	63
45	Epinephelus lanceolatus	Giant Grouper	37	R	S	L	C	E	R	H	F	L	G	V	F	S	K	V	R	F	C	S	G	K	K	R	P	V	R	R	63
46	Paramormyrops kingsleyae	Paramormyrops kingsleyae	37	R	S	L	C	E	R	H	F	L	G	V	F	S	K	V	R	F	C	S	G	K	K	R	P	V	R	R	63
47	Betta splendens	Siamese Fighting Fish	37	R	S	L	C	E	R	H	F	L	G	V	F	S	K	V	R	F	C	S	G	K	K	R	P	V	R	R	63
48	Anabas testudineus	Climbing Perch	38	R	S	L	C	E	R	H	I	F	S	V	F	S	K	V	R	F	C	S	G	K	K	R	P	V	R	R	64
49	Notolabrus celidotus	Spotty	37	R	S	L	C	E	R	H	F	L	G	V	F	S	K	V	R	F	C	S	G	K	K	R	P	V	R	R	63
50	Oryzias melastigma	Marine Medaka	37	R	S	L	C	E	R	H	F	L	G	V	F	S	K	V	R	F	C	S	G	K	K	R	P	V	R	R	63
51	Oryzias latipes	Japanese Rice Fish	37	R	S	L	C	E	R	H	F	L	G	V	F	S	K	V	R	F	C	S	G	K	K	R	P	V	R	R	63
52	Fundulus heteroclitus	Mummichog	37	R	S	L	C	E	R	H	F	L	G	V	F	S	K	V	R	F	C	S	G	K	K	R	P	V	R	R	63
53	Boleophthalmus pectinirostris	Mudskipper	37	R	S	L	C	E	R	H	F	L	G	V	F	S	K	V	R	F	C	S	G	K	K	R	P	V	R	R	63
54	Labrus bergylta	Ballan Wrasse	37	R	S	L	C	E	R	H	F	L	G	V	F	S	K	V	R	F	C	S	G	K	K	R	P	V	R	R	63
55	Oreochromis niloticus	Nile Tilapia	37	R	S	L	C	E	R	H	F	L	G	V	F	S	K	V	R	F	C	S	G	K	K	R	P	V	R	R	63
56	Hippoglossus hippoglossus	Atlantic Halibut	37	R	S	L	C	E	R	H	F	L	G	V	F	S	K	V	R	F	C	S	G	K	K	R	P	V	R	R	63

Table S7
(*Related to Fig. 2*)
Comparison of FGF13A CaMBD Sequences From 53 Species

CaMBD Sequence Table (Completely Conserved Positions Highlighted in Yellow, Positions Conserved in ≥ 90% of Species Highlighted in Green)

	Species	Common Name	Start Residue	35	36	37	38	39	40	41	42	43	44	45	46	47	48	49	50	51	52	53	54	55	56	57	58	59	60	End Residue
				K					K	N	K	L		V	F	S	R	V		L	F		S	K	K					
1	Homo sapiens	Human	35	K	T	S	C	D	K	N	K	L	N	V	F	S	R	V	K	L	F	G	S	K	K	R	R	R	R	60
2	Myotis davidii	David's Myotis	35	K	T	S	C	D	K	N	K	L	N	V	F	S	R	V	K	L	F	G	S	K	K	R	R	R	R	60
3	Meriones unguiculatus	Mongolian Gerbil	35	K	T	S	C	D	K	N	K	L	N	V	F	S	R	V	K	L	F	G	S	K	K	R	R	R	R	60
4	Myotis brandtii	Brandt's Bat	35	K	T	S	C	D	K	N	K	L	N	V	F	S	R	V	K	L	F	G	S	K	K	R	R	R	R	60
5	Tupaia chinensis	Treeshrew	35	K	T	S	C	D	K	N	K	L	N	V	F	S	R	V	K	L	F	G	S	K	K	R	R	R	R	60
6	Camelus dromedarius	Dromedary	35	K	T	S	C	D	K	N	K	L	N	V	F	S	R	V	K	L	F	G	S	K	K	R	R	R	R	60
7	Vicugna pacos	Alpaca	35	K	T	S	C	D	K	N	K	L	N	V	F	S	R	V	K	L	F	G	S	K	K	R	R	R	R	60
8	Rhinolophus ferrumequinum	Greater Horseshoe Bat	35	K	T	S	C	D	K	N	K	L	N	V	F	S	R	V	K	L	F	G	S	K	K	R	R	R	R	60
9	Bos taurus	Cattle	35	K	T	S	C	D	K	N	K	L	N	V	F	S	R	V	K	L	F	G	S	K	K	R	R	R	R	60
10	Suricata suricatta	Meerkat	35	K	T	S	C	D	K	N	K	L	N	V	F	S	R	V	K	L	F	G	S	K	K	R	R	R	R	60
11	Loxodonta africana	African Bush Elephant	35	K	T	S	C	D	K	N	K	L	N	V	F	S	R	V	K	L	F	G	S	K	K	R	R	R	R	60
12	Cavia porcellus	Guinea Pig	35	K	T	S	C	D	K	N	K	L	N	V	F	S	R	V	K	L	F	G	S	K	K	R	R	R	R	60
13	Pongo abelii	Sumatran Orangutan	35	K	T	S	C	D	K	N	K	L	N	V	F	S	R	V	K	L	F	G	S	K	K	R	R	R	R	60
14	Sus scrofa	Wild Boar	35	K	T	S	C	D	K	N	K	L	N	V	F	S	R	V	K	L	F	G	S	K	K	R	R	R	R	60
15	Otolemur garnettii	Northern Greater Galago	35	K	T	S	C	D	K	N	K	L	N	V	F	S	R	V	K	L	F	G	S	K	K	R	R	R	R	60
16	Urocitellus parryii	Arctic Ground Squirrel	35	K	T	S	C	D	K	N	K	L	N	V	F	S	R	V	K	L	F	G	S	K	K	R	R	R	R	60
17	Mesocricetus auratus	Golden Hamster	35	K	T	S	C	D	K	N	K	L	N	V	F	S	R	V	K	L	F	G	S	K	K	R	R	R	R	60
18	Macaca fascicularis	Crab-eating Macaque	35	K	T	S	C	D	K	N	K	L	N	V	F	S	R	V	K	L	F	G	S	K	K	R	R	R	R	60
19	Heterocephalus glaber	Naked Mole-rat	35	K	T	S	C	D	K	N	K	L	N	V	F	S	R	V	K	L	F	G	S	K	K	R	R	R	R	60
20	Mus musculu	House Mouse	35	K	T	S	C	D	K	N	K	L	N	V	F	S	R	V	K	L	F	G	S	K	K	R	R	R	R	60
21	Canis lupus familiaris	Dog	35	K	T	S	C	D	K	N	K	L	N	V	F	S	R	V	K	L	F	G	S	K	K	R	R	R	R	60
22	Rattus rattus	Black Rat	35	K	T	S	C	D	K	N	K	L	N	V	F	S	R	V	K	L	F	G	S	K	K	A	Q	K	G	60
23	Limosa lapponica baueri	Bar-tailed Godwit	35	K	G	S	C	D	K	N	K	L	N	V	F	S	R	V	K	L	F	G	S	K	K	R	R	R	R	60
24	Nicator chloris	Western Nicator	35	K	G	S	C	D	K	N	K	L	N	V	F	S	R	V	K	L	F	G	S	K	K	R	R	R	R	60
25	Malurus elegans	Red-winged Fairywren	35	K	G	S	C	D	K	N	K	L	N	V	F	S	R	V	K	L	F	G	S	K	K	R	R	R	R	60
26	Origma solitaria	Rockwarbler	35	K	G	S	C	D	K	N	K	L	N	V	F	S	R	V	K	L	F	G	S	K	K	R	R	R	R	60
27	Acrocephalus arundinaceus	Great Reed Warbler	35	K	G	S	C	D	K	N	K	L	N	V	F	S	R	V	K	L	F	G	S	K	K	R	R	R	R	60
28	Mionectes macconnelli	McConnell's Flycatcher	35	K	G	S	C	D	K	N	K	L	N	V	F	S	R	V	K	L	F	G	S	K	K	R	R	R	R	60
29	Drymodes brunneopygia	Southern Scrub Robin	35	K	G	S	C	D	K	N	K	L	N	V	F	S	R	V	K	L	F	G	S	K	K	R	R	R	R	60
30	Todus mexicanus	Puerto Rican Tody	35	K	G	S	C	D	K	N	K	L	N	V	F	S	R	V	K	L	F	G	S	K	K	R	R	R	R	60
31	Hirundo rustica	Barn Swallow	35	K	G	S	C	D	K	N	K	L	N	V	F	S	R	V	R	L	F	G	S	K	K	R	R	R	R	60
32	Pedionomus torquatus	Plains-wanderer	35	K	G	N	C	D	K	N	K	L	N	V	F	S	R	V	K	L	F	G	S	K	K	R	R	R	R	60
33	Alligator sinensis	Chinese Alligator	35	K	G	S	C	D	K	N	K	L	N	V	F	S	R	V	K	L	F	G	S	K	K	R	R	R	R	60
34	Erithacus rubecula	European Robin	35	K	G	S	C	D	K	N	K	L	N	V	F	S	R	V	K	L	F	G	S	K	K	R	R	R	R	60
35	Phascolarctos cinereus	Koala	35	K	G	S	C	D	K	N	K	L	N	V	F	S	R	V	K	L	F	G	S	K	K	R	R	R	R	60
36	Alaudala cheleensis	Asian Short-Toed Lark	35	K	G	S	C	D	K	N	K	L	N	V	F	S	R	V	K	L	F	G	S	K	K	R	R	R	R	60
37	Chloropsis hardwickii	Orange-bellied Leafbird	35	K	G	S	C	D	K	N	K	L	N	V	F	S	R	V	K	L	F	G	S	K	K	R	R	R	R	60
38	Turnix velox	Little Buttonquail	35	K	G	S	C	D	K	N	K	L	N	V	F	S	R	V	K	L	F	G	S	K	K	R	R	R	R	60
39	Taeniopygia guttata	Zebra Finch	35	K	G	S	C	D	K	N	K	L	N	V	F	S	R	V	K	L	F	G	S	K	K	R	R	R	R	60
40	Catharus ustulatus	Swainson's Thrush	35	K	G	S	C	D	K	N	K	L	N	V	F	S	R	V	K	L	F	G	S	K	K	R	R	R	R	60
41	Serinus canaria	Atlantic Canary	35	K	G	S	C	D	K	N	K	L	N	V	F	S	R	V	K	L	F	G	S	K	K	R	R	R	R	60
42	Gallus gallus	Red Junglefowl	35	K	G	S	C	D	K	N	K	L	N	V	F	S	R	V	K	L	F	G	S	K	K	R	R	R	R	60
43	Ornithorhynchus anatinus	Platypus	35	K	G	S	C	D	K	N	K	L	N	V	F	S	R	V	K	L	F	G	S	K	K	R	R	R	R	60
44	Cercotrichas coryphoeus	Karoo Scrub Robin	35	K	G	S	C	D	K	N	K	L	N	V	F	S	R	V	K	L	F	G	S	K	K	R	R	R	R	60
45	Turdus rufiventris	Rufous-bellied thrush	35	K	G	N	C	D	K	N	K	L	N	V	F	S	R	V	K	L	F	G	S	K	K	R	R	R	R	60
46	Xenopus tropicalis	Western Clawed Frog	35	K	G	N	C	E	K	N	K	L	N	V	F	S	R	V	K	L	F	G	S	K	K	R	R	R	R	60
47	Rhinatrema bivittatum	Rhinatrema bivittatum	35	K	G	N	C	E	K	N	K	L	S	V	F	S	R	V	K	L	F	G	S	K	K	R	R	R	R	60
48	Chelonia mydas	Green Sea Turtle	35	K	G	S	C	E	K	N	K	L	N	V	F	S	R	V	K	L	F	S	S	K	K	R	R	R	R	60
49	Geotrypetes seraphini	Gaboon Caecilian	35	K	G	N	S	E	K	N	K	L	S	V	F	S	R	V	K	L	F	G	S	K	K	R	R	R	R	60
50	Gopherus evgoodei	Desert Tortoise	35	K	G	S	C	E	K	N	K	L	N	V	F	S	R	V	K	L	F	S	S	K	K	R	R	R	R	60
51	Chrysemys picta bellii	Western Painted Turtle	35	K	G	S	C	E	K	N	K	L	N	V	F	S	R	V	K	L	F	S	S	K	K	R	R	R	R	60
52	Papio anubis	Olive Baboon	35	K	T	S	C	D	K	N	K	L	N	V	F	S	R	V	K	L	F	G	S	K	K	R	P	R	R	60
53	Macaca mulatta	Rhesus Macaque	35	K	T	S	C	D	K	N	K	L	N	V	F	S	R	V	K	L	F	G	S	K	K	R	P	R	R	60

Table S8

(Related to Fig. 2)

Comparison of FGF14A CaMBD Sequences From 48 Species

CaMBD Sequence Table (Completely Conserved Positions Highlighted in Yellow, Positions Conserved in ≥ 90% of Species Highlighted in Green)

					G	L	C	N		N	L	V	D	I	F		K	V	R	I	F	G			K	R	R	L	R	R	
	Species	Common Name	Start Residue	37	38	39	40	41	42	43	44	45	46	47	48	49	50	51	52	53	54	55	56	57	58	59	60	61	62	63	End Residue
1	Calypte anna	Anna's Hummingbird	37	R	G	L	C	N	G	N	L	V	D	I	F	S	K	V	R	I	F	G	L	K	K	R	R	L	R	R	63
2	Scleropages formosus	Asian Arowana	37	K	G	L	C	N	G	N	L	V	D	I	F	S	K	V	R	I	F	G	L	R	K	R	R	L	R	R	63
3	Monopterus albus	Asian Swamp Eel	37	K	G	L	C	N	G	N	L	V	D	I	F	S	K	V	R	I	F	G	L	R	K	R	R	L	R	R	63
4	Alectura lathami	Australian Brushturkey	37	R	G	L	C	N	G	N	L	V	D	I	F	S	K	V	R	I	F	G	L	K	K	R	R	L	R	R	63
5	Limosa lapponica baueri	Bar-tailed Godwit	37	R	G	L	C	N	G	N	L	V	D	I	F	S	K	V	R	I	F	G	L	K	K	R	R	L	R	R	63
6	Pteropus alecto	Black Flying Fox	37	R	G	L	C	N	G	N	L	V	D	I	F	S	K	V	R	I	F	G	L	K	K	R	R	L	R	R	63
7	Rattus rattus	Black Rat	37	R	G	L	C	N	G	N	L	V	D	I	F	S	K	V	R	I	F	G	L	K	K	R	R	L	R	R	63
8	Felis catus	Cat	37	R	G	L	C	N	G	N	L	V	D	I	F	S	K	V	R	I	F	G	L	K	K	R	R	L	R	R	63
9	Cricetulus griseus	Chinese Hamster	37	R	G	L	C	N	G	N	L	V	D	I	F	S	K	V	R	I	F	G	L	K	K	R	R	L	R	R	63
10	Desmodus rotundus	Common Vampire Bat	37	R	G	L	C	N	S	N	L	V	D	I	F	S	K	V	R	I	F	G	L	K	K	R	R	L	R	R	63
11	Cottoperca gobio	Cottoperca	37	K	G	L	C	N	G	N	L	V	D	I	F	S	K	V	R	I	F	G	L	R	K	R	R	L	R	R	63
12	Cynoglossus semilaevis	Cynoglossus semilaevis	37	K	G	L	C	N	G	N	L	V	D	I	F	S	K	V	R	I	F	G	L	R	K	R	R	L	R	R	63
13	Denticeps clupeoides	Denticle Herring	37	K	G	L	C	N	G	N	L	V	D	I	F	S	K	V	R	I	F	G	L	R	K	R	R	L	R	R	63
14	Canis lupus dingo	Dingo	37	R	G	L	C	N	G	N	L	V	D	I	F	S	K	V	R	I	F	G	L	K	K	R	R	L	R	R	63
15	Camelus dromedarius	Dromedary	37	R	G	L	C	N	G	N	L	V	D	I	F	S	K	V	R	I	F	G	L	K	K	R	R	L	R	R	63
16	Electrophorus electricus	Electric Eel	37	K	G	L	C	N	G	N	L	V	D	I	F	S	K	V	R	I	F	G	L	R	K	R	R	L	R	R	63
17	Anguilla anguilla	European Eel	37	K	G	L	C	N	G	N	L	V	D	I	F	S	K	V	R	I	F	G	L	R	K	R	R	L	R	R	63
18	Geotrypetes seraphini	Gaboon Caecilian	37	R	G	L	C	N	G	N	L	V	D	I	F	S	K	V	R	I	F	G	I	R	K	R	R	L	R	R	63
19	Aquila chrysaetos chrysaetos	Golden Eagle	37	R	G	L	C	N	G	N	L	V	D	I	F	S	K	V	R	I	F	G	L	K	K	R	R	L	R	R	63
20	Microcebus murinus	Gray Mouse Lemur	37	R	G	L	C	N	G	N	L	V	D	I	F	S	K	V	R	I	F	G	L	K	K	R	R	L	R	R	63
21	Mus musculus	House Mouse	37	R	G	L	C	N	G	N	L	V	D	I	F	S	K	V	R	I	F	G	L	K	K	R	R	L	R	R	63
22	Homo sapiens	Human	37	R	G	L	C	N	G	N	L	V	D	I	F	S	K	V	R	I	F	G	L	K	K	R	R	L	R	R	63
23	Clarias magur	Indian Catfish	37	K	G	L	C	N	G	N	L	V	D	I	F	A	K	V	R	I	F	G	L	R	K	R	R	L	R	R	63
24	Oryzias latipes	Japanese Rice Fish	37	K	G	L	C	N	G	N	L	V	D	I	F	S	K	V	R	I	F	G	L	R	K	R	R	L	R	R	63
25	Trichechus manatus latirostris	Manatee	37	R	G	L	C	N	G	N	L	V	D	I	F	S	K	V	R	I	F	G	L	K	K	R	R	L	R	R	63
26	Microcaecilia unicolor	Microcaecilia unicolor	37	R	G	L	C	N	G	N	L	V	D	I	F	S	K	V	R	I	F	G	I	R	K	R	R	L	R	R	63
27	Chanos chanos	Milkfish	37	K	G	L	C	N	G	N	L	V	D	I	F	S	K	V	R	I	F	G	L	R	K	R	R	L	R	R	63
28	Fundulus heteroclitus	Mummichog	37	K	G	L	C	N	G	N	L	V	D	I	F	S	K	V	R	I	F	G	L	R	K	R	R	L	R	R	63
29	Dasypus novemcinctus	Nine-banded Armadillo	37	R	G	L	C	N	G	N	L	V	D	I	F	S	K	V	R	I	F	G	L	K	K	R	R	L	R	R	63
30	Paramormyrops kingsleyae	Paramaormyrops	37	K	G	L	C	N	G	N	L	V	D	I	F	S	K	V	R	I	F	G	L	R	K	R	R	L	R	R	63
31	Chelonoidis abingdonii	Pinta Island Tortoise	37	R	G	L	C	N	G	N	L	V	D	I	F	S	K	V	R	I	F	G	L	K	K	R	R	L	R	R	63
32	Microtus ochrogaster	Prairie Vole	37	R	G	L	C	N	G	N	L	V	D	I	F	S	K	V	R	I	F	G	L	K	K	R	R	L	R	R	63
33	Takifugu flavidus	Pufferfish	37	K	G	L	C	N	G	N	L	V	D	I	F	S	K	V	R	I	F	G	L	R	K	R	R	L	R	R	63
34	Gallus gallus	Red Junglefowl	37	R	G	L	C	N	G	N	L	V	D	I	F	S	K	V	R	I	F	G	L	K	K	R	R	L	R	R	63
35	Erpetoichthys calabaricus	Reedfish	37	R	G	L	C	N	G	N	L	V	D	I	F	S	K	V	R	I	F	G	L	R	K	R	R	L	R	R	63
36	Xiphophorus maculatus	Souther Platyfish	37	K	G	L	C	N	G	N	L	V	D	I	F	S	K	V	R	I	F	G	L	R	K	R	R	L	R	R	63
37	Mirounga leonina	Southern Elephant Seal	37	R	G	L	C	N	G	N	L	V	D	I	F	S	K	V	R	I	F	G	L	K	K	R	R	L	R	R	63
38	Nannospalax galili	Spalax	37	R	G	L	C	N	G	N	L	V	D	I	F	S	K	V	R	I	F	G	L	K	K	R	R	L	R	R	63
39	Mustela erminea	Stoat	37	R	G	L	C	N	G	N	L	V	D	I	F	S	K	V	R	I	F	G	L	K	K	R	R	L	R	R	63
40	Thalassophryne amazonica	Toadfish	37	K	G	L	C	N	G	N	L	V	D	I	F	S	K	V	R	I	F	G	L	R	K	R	R	L	R	R	63
41	Amazona aestiva	Turquoise-fronted Amazon	37	R	G	L	C	N	G	N	L	V	D	I	F	S	K	V	R	I	F	G	L	K	K	R	R	L	R	R	63
42	Rhinatrema bivittatum	Two-tailed Caecilian	37	R	G	L	C	N	G	N	L	V	D	I	F	S	K	V	R	I	F	G	L	R	K	R	R	L	R	R	63
43	Bubalus bubalis	Water Buffalo	37	R	G	L	C	N	G	N	L	V	D	I	F	S	K	V	R	I	F	G	L	K	K	R	R	L	R	R	63
44	Xenopus tropicalis	Western Clawed Frog	37	R	G	L	C	N	G	N	L	V	D	I	F	S	K	V	R	I	F	G	L	R	K	R	R	L	R	R	63
45	Chrysemys picta bellii	Western Painted Turtle	37	R	G	L	C	N	G	N	L	V	D	I	F	S	K	V	R	I	F	G	L	K	K	R	R	L	R	R	63
46	Zonotrichia albicollis	White-throated Sparrow	37	R	G	L	C	N	G	N	L	V	D	I	F	S	K	V	R	I	F	G	L	K	K	R	R	L	R	R	63
47	Sus scrofa	Wild Boar	37	R	G	L	C	N	G	N	L	V	D	I	F	S	K	V	R	I	F	G	L	K	K	R	R	L	R	R	63
48	Mastacembelus armatus	Zig-zag Eel	37	K	G	L	C	N	G	N	L	V	D	I	F	S	K	V	R	I	F	G	L	R	K	R	R	L	R	R	63
